# Innovative mouse models for the tumor suppressor activity of Protocadherin-10 isoforms

**DOI:** 10.1186/s12885-022-09381-y

**Published:** 2022-04-25

**Authors:** Irene Kleinberger, Ellen Sanders, Katrien Staes, Marleen Van Troys, Shinji Hirano, Tino Hochepied, Kelly Lemeire, Liesbet Martens, Christophe Ampe, Frans van Roy

**Affiliations:** 1 Department of Biomedical Molecular Biology, Ghent University, Technologiepark-Zwijnaarde 71, 9052 Ghent, Belgium; 2 VIB-UGent Center for Inflammation Research (IRC), VIB, 9052 Ghent, Belgium; 3 Department of Biomolecular Medicine, Faculty of Medicine and Health Sciences, Ghent University, 9052 Ghent, Belgium; 4 Department of Cell Biology, Kansai Medical University, Hirakata City, Osaka, 573-1010 Japan; 5 Cancer Research Institute Ghent (CRIG), 9052 Ghent, Belgium

**Keywords:** Protocadherin-10 isoforms, Tumor suppression, Conditional gene knockout, GFAP-Cre, Mouse medulloblastoma model, Mouse auricular tumors, Somatic stem cells, Tumor-derived cells, Allografts, RNA-Seq

## Abstract

**Background:**

Nonclustered mouse protocadherin genes *(Pcdh)* encode proteins with a typical single ectodomain and a cytoplasmic domain with conserved motifs completely different from those of classic cadherins. Alternative splice isoforms differ in the size of these cytoplasmic domains. In view of the compelling evidence for gene silencing of protocadherins in human tumors, we started investigations on *Pcdh* functions in mouse cancer models.

**Methods:**

For *Pcdh10*, we generated two mouse lines: one with floxed exon 1, leading to complete Pcdh10 ablation upon Cre action, and one with floxed exons 2 and 3, leading to ablation of only the long isoforms of Pcdh10. In a mouse medulloblastoma model, we used GFAP-Cre action to locally ablate *Pcdh10* in combination with *Trp53* and *Rb1* ablation. From auricular tumors, that also arose, we obtained tumor-derived cell lines, which were analyzed for malignancy in vitro and in vivo. By lentiviral transduction, we re-expressed Pcdh10 cDNAs. RNA-Seq analyses were performed on these cell families.

**Results:**

Surprisingly, not only medulloblastomas were generated in our model but also tumors of tagged auricles (pinnae). For both tumor types, ablation of either all or only long isoforms of Pcdh10 aggravated the disease. We argued that the perichondrial stem cell compartment is at the origin of the pinnal tumors. Immunohistochemical analysis of these tumors revealed different subtypes. We obtained several pinnal-tumor derived (PTD) cell lines and analyzed these for anchorage-independent growth, invasion into collagen matrices, tumorigenicity in athymic mice. Re-expression of either the short or a long isoform of Pcdh10 in two PTD lines counteracted malignancy in all assays. RNA-Seq analyses of these two PTD lines and their respective Pcdh10-rescued cell lines allowed to identify many interesting differentially expressed genes, which were largely different in the two cell families.

**Conclusions:**

A new mouse model was generated allowing for the first time to examine the remarkable tumor suppression activity of protocadherin-10 in vivo. Despite lacking several conserved motifs, the short isoform of Pcdh10 was fully active as tumor suppressor. Our model contributes to scrutinizing the complex molecular mechanisms of tumor initiation and progression upon *PCDH10* silencing in many human cancers.

**Supplementary Information:**

The online version contains supplementary material available at 10.1186/s12885-022-09381-y.

## Background

**Fig. 1:**
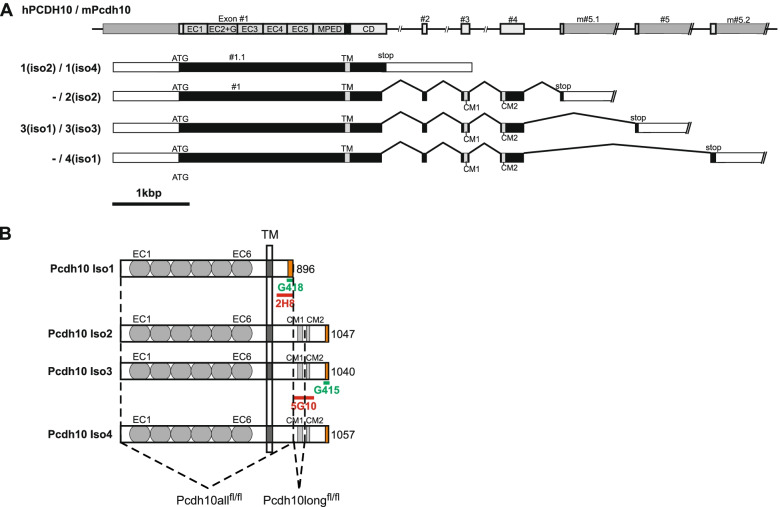
Gene structure, transcripts and protein structure of human PCDH10 and mouse Pcdh10 isoforms. **A** Isoform numbers before slash signs indicate human transcripts, isoform numbers after slash signs indicate mouse transcripts. Isoform 2 and 4 have not yet been identified in humans. Variant annotations between parentheses refer to nomenclature from literature, but lack overall consistency in the δ-PCDH family. Gray boxes in the gene represent exons (drawn to scale), the intervening lines represent intronic sequences (introns not to scale). Black boxes in the transcripts represent coding sequences, white boxes represent 5′ or 3′ untranslated regions. CD, sequence in exon 1 encoding the membrane-proximal part of the cytoplasmic domain; CM1, and CM2, conserved motifs; EC, sequences encoding the extracellular cadherin repeats; MPED, sequence encoding the membrane-proximal extracellular domain; TM, sequence encoding the transmembrane domain. **B** Schematic representation of mouse Pcdh10 proteins. All proteins are drawn to the same scale and aligned at their transmembrane domain (TM). Their total size is indicated on the right (number of amino acid residues). Differences in the amino acid sequences between the four isoforms are highlighted in orange. Colored horizontal lines indicate the approximate locations of the epitopes of the isoform-specific monoclonal (2H8 and 5G10, red) and polyclonal (G418 and G415, green) antibodies. Dashed lines indicate the regions that were floxed in Pcdh10all^fl/fl^ and Pcdh10long^fl/fl^ mice, respectively. Upon Cre-mediated deletion the predicted consequences are the removal of exon 1 in Pcdh10all^fl/fl^ mice, leading to a complete KO of all Pcdh10 isoforms, and the removal of exons 2 and 3 in Pcdh10long^fl/fl^ mice, leading to a frameshift in exon 4 and 5, so that only the short isoform is expected to be expressed. See **A** for abbreviations

Protocadherin-10 (PCDH10), originally called OL-protocadherin [1], belongs to the delta-protocadherin (δ-Pcdh) family [2], which is part of the important cadherin superfamily of cell-cell adhesion molecules, and is encoded by the non-clustered *PCDH10* gene [3]. The ectodomains of δ-protocadherins form preferentially homophilic adhesive *trans* dimers but lack cis-dimerization [4]. All δ-Pcdhs are expressed as multiple isoforms differing in their cytoplasmic domains. Human PCDH10 is expressed as one short isoform and one long isoform (Fig. 1A). Mouse Pcdh10 is expressed as one short isoform 1 (iso1) and three long isoforms (iso2 to iso4) (Fig. 1). Interestingly, the longer isoforms of δ-Pcdhs share several evolutionary conserved motifs in their cytoplasmic tails [2, 5], suggesting that putative common interaction partners might be important players in some basic biological functions of these still largely enigmatic proteins. Among these interacting proteins is for instance Nck-associated protein 1 (Nap1), a subunit of the WAVE regulatory complex (WRC) [6], which binds to the conserved peptide motif WIRS in the cytoplasmic domain of protocadherin-10 [5]. The PCDH10-WRC interaction was shown to promote a kind of uncoordinated migration of astrocytoma cells in contact with each other, but not in case of solitary cells, probably by focal redistribution of N-cadherin [6].

In recent years, direct and indirect evidence has accumulated that δ-Pcdhs are important during tumorigenesis, either as tumor-suppressor genes or as oncogenes [7–10]. It has been reported that human *PCDH10*, located at chromosome 4q28.3, is frequently inactivated in various human cancers suggesting that PCDH10 acts as tumor suppressor in these malignancies. A focal homozygous deletion of *PCDH10* was seen in a medulloblastoma patient [11], and 53 out of 171 colorectal cancer patients showed allelic deletion of *PCDH10* [12]. Moreover, *PCDH10* was found to be frequently silenced epigenetically by promoter hypermethylation in numerous human malignancies. Since the original report of *PCDH10* promoter methylation in breast cancer [13], this phenomenon has been detected in medulloblastomas, nasopharyngeal and esophageal carcinomas, gastric cancer, colorectal cancer, hepatocellular carcinoma, pancreatic cancer, breast cancer, cervical cancer, testicular cancer, prostate cancer, bladder cancer, non-small cell lung cancer and multiple haematologic malignancies [8–10]. *PCDH10* methylation does not occur in normal tissues.

Importantly, tumor-associated *PCDH10* silencing may be quite useful for early detection of occurrence and/or progression of multiple human cancers [10]. For instance, increased *PCDH10* promoter methylation was associated significantly with poorer survival of gastric cancer patients, and identified to be an independent prognostic indicator of this cancer type [14, 15]. The frequent genetic deletion of *PCDH10* in colorectal cancers was significantly associated with tumor progression and distant metastasis and found to be an independent predictor of poor survival [12]. Low expression of PCDH10 mRNA in hepatocellular carcinoma specimens was associated with a worse overall survival and this was an independent prognostic indicator [16]. Also for very lethal pancreatic ductal adenocarcinoma *PCDH10* promoter methylation significantly associated with worse progression-free survival although not overall survival [17]. Hormone-receptor positive breast cancer patients with hypermethylation of the *PCDH10* promoter showed significantly poorer disease outcome than patients with unmethylated *PCDH10* [18, 19]. The methylation status of *PCDH10* starts at very early stages of cervical cancer and is highly associated with the severity of the disease [20–22]. Its analysis in cervical scrapings is superior to the human papillomavirus (HPV) test. Increased *PCDH10* promoter methylation was also significantly associated with increased malignancy (invasion, metastasis, recurrence) of prostate cancers, and an independent prognostic biomarker of worse recurrence-free survival of patients after radical prostatectomy [23]. Likewise, *PCDH10* promoter methylation was significantly associated with tumor recurrence and shortened survival of patients with bladder transitional cell carcinoma [24]. In these patients, both *PCDH10* promoter methylation and downregulated PCDH10 protein levels were independent predictors of decreased overall survival [24, 25]. Patients with curatively resected pathological stage I non-small-cell lung cancer patients showed significantly worse survival (recurrence-free, overall or disease-specific) in case of *PCDH10* promoter methylation [26]. In diffuse large B-cell lymphoma patients, treated with the drug combination R-CHOP, promoter methylation of *PCDH10* was an independent prognostic indicator of worse overall survival and worse progress-free survival [27]. Moreover, silencing of *PCDH10* contributes to chemotherapy resistance of leukemia and lymphomas [28, 29] and might therefore serve as an indicator of drug resistance.

Ectopic expression of the short isoform 1 of mouse Pcdh10 in mouse L and Neuro2A cells has been used by Hirano et al. [1] to demonstrate the rather weak but homophilic cell-cell adhesion activity of this newly described protocadherin. Nakao et al. [6] introduced the long isoform 3 of mouse Pcdh10 into U252 human astrocytoma cells and observed a peculiar effect on migration by cells in mutual contact. Regarding ectopic re-expression of human PCDH10, such experiments have been reported for a multitude of different human cancer cell lines, but as far as we could find out, this was done exclusively for the long isoform of PCDH10 [11, 12, 14, 30–45]. Interesting recent experiments included knockdown of PCDH10 by shRNA in a T-cell lymphoma cell line [44], and conditional induction of PCDH10 in a non-small cell lung cancer cell line and a colorectal cancer cell line [38, 45]. Frequent observations upon PCDH10 re-expression in these many reports were: reduced in vitro cell proliferation, reduced in vitro colony formation, reduced in vitro anchorage-independent growth, reduced monolayer wound healing, reduced in vitro invasion into Matrigel matrices, reduced angiogenesis, reduced telomerase activity, increased in vitro apoptosis and reversal of epithelial-mesenchymal transition. Some exceptions were also reported, including unchanged in vitro cell proliferation [11, 38], no increase in apoptosis [31, 38], or no effect on monolayer wound healing [35]. Interestingly, some studies reported on slower growth of subcutaneous xenografts in immunodeficient mice [12, 14, 31, 35], and decreased liver colonisation after splenic injection of colorectal cancer cell lines [12].

Several signalling pathways were found to be modified by PCDH10 expression or re-expression: increased pro-apoptotic NFκB signalling in multiple myeloma [36], decreased β-catenin/Wnt signalling in endometroid endometrial cancer cell lines, multiple myeloma, lymphoma and colorectal carcinoma cells [35, 37, 42, 45], and inhibition of the PI3K/Akt signalling pathway in hepatocellular and colorectal carcinoma cells [43, 45]. In breast and gastrointestinal cancer cell lines, oncogenic long non-coding RNAs HOTAIR and MALAT1 were found to antagonize *PCDH10* by inducing the methylation of its promoter [40, 46–48]. On the other hand, *PCDH10* was reported to be a direct transcriptional target of wild-type but not oncogenic p53 in various cancer cell lines [38].

An animal model for the tumor suppressor activity of PCDH10 has not been reported. A mouse with *Pcdh10*-null alleles has been generated previously by replacing the first long exon, transcribed in all four transcript types, by a lacZ-neo selection cassette [49]. Brain development was studied in this interesting mouse, but homozygous mutant mice showed early lethality, possibly due to failure of striatal axon outgrowth [49]. On the other hand, heterozygous mice of this mutant strain were successfully used as model for neural disorders [50–52]. Indeed, *PCDH10* is an autism spectrum disorder gene, and a region (PIR, proteasome interacting region), shared by the C-termini of both short and long isoforms of Pcdh10, is instrumental in excitatory synapse elimination by linking ubiquinated PSD-95 to the proteasome [53].

In summary, inactivation of *PCDH10* is widespread in a large variety of human cancers. Re-expression experiments have consolidated the importance of PCDH10 expression in tumor suppression. However, the activity of long versus short isoforms has not been compared. Animal cancer models with *Pcdh10* inactivation have not been reported. A *Pcdh10*-null mouse is available but cannot be used for tumorigenicity studies. Therefore, we have generated and extensively studied exclusive mouse models in which either all isoforms or only long isoforms of Pcdh10 can be conditionally ablated. In the present study, we have used these engineered mice in a study of medulloblastoma generation. In addition to this tumor type, we observed and analyzed generation of peculiar tumors in the auricles (pinnae).

## Methods

### Animals

Mice were bred and housed in individually ventilated cages in the specific-pathogen-free animal facility of the IRC department. All experiments on mice were conducted according to institutional, national, and European animal regulations and guidelines (See Ethics Approval in Declaration Section). In this report, use of the ^−/−^ notation means that the animals are GFAP-Cre positive, and that the knockout status was confirmed for the floxed genes indicated. The progenitor p53^fl/fl^ mice and Rb1^fl/fl^ mice were kindly provided by Dr. A. Berns (Netherlands Cancer Institute, Amsterdam, The Netherlands) [54]. GFAP-Cre mice, expressing Cre recombinase under the control of the *GFAP* promoter [55], were obtained from Dr. Jody Haigh. Nestin-Cre mice [56] were obtained from the Jackson Laboratory (Jax #002858). The Rosa26^Tg/+^ mouse was generated in house [57]. R26R reporter mice [58] were obtained from the Jackson Laboratory (Jax #003474).

### Conditional knockout of either all or long isoforms of the *Pcdh10* allele

**Fig. 2:**
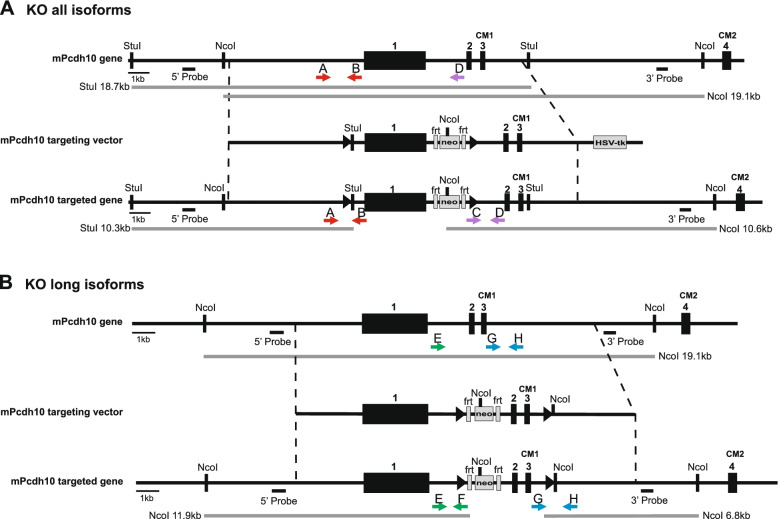
Generation of recombinant mice with floxed *Pcdh10* genes. **A** Targeting strategy to generate Pcdh10all^fl/fl^ mice. loxP sites (black arrowheads) were inserted downstream and upstream of exon 1. A neo-resistance cassette (neo) flanked by FRT sites was inserted downstream of exon 1 for selection of correctly targeted embryonic stem (ES) cells. An HSV-tk cassette was cloned into the targeting vector for negative selection of ES cells with random integration of the targeting vector. **B** Targeting strategy to generate Pcdh10long^fl/fl^ mice. loxP sites were inserted upstream of exon 2 and downstream of exon 3, respectively. A neo cassette flanked by FRT sites was inserted upstream of exon 2 for selection of correctly targeted ES cells. **A and B** 5′ and 3′ probes and restriction enzyme sites for Southern blot analysis of ES cells are indicated and expected fragments are shown in grey (Results in Additional file 4: Fig. S3A). Colored arrows indicate the position of genotyping primers used to check the 5′ (red or green arrows) and 3′ (purple and blue arrows) recombined regions (Additional file 4: Fig. S3B). Black rectangles, exons 1–4; CM1 and CM2, conserved motifs

As shown in Fig. 2A, we constructed a *Pcdh10* gene targeting vector comprising two loxP sites sandwiching exon 1, a positive selection marker flanked by FRT sequences and inserted into intron 1, and a negative selection marker following the genomic fragment. The detailed procedure for cloning the targeting vector by recombineering [59] is described in Additional file 1. To target only the long isoforms of Pcdh10, we constructed a *Pcdh10* gene targeting vector comprising two loxP sites sandwiching exons 2 and 3, and a neo-resistance cassette flanked by FRT sequences and inserted into intron 1, downstream of the first loxP site (Fig. 2B). The detailed procedure for cloning the targeting vector by recombineering is described in Additional file 2. For homologous recombination, the targeting vector was linearized and electroporated in G4 embryonic stem (ES) cells [60], with genetic background (129S6xC57BL/6 N)F1. The generation and subsequent selection of recombinant ES cell lines were performed by the Transgenic mouse core facility (TMCF) of the IRC department (VIB, Ghent University). About 20 μg of plasmid DNA was linearized overnight at 37 °C, run on an agarose gel, and the correct band was isolated using the Qiagen gel extraction kit (Qiagen Benelux, Antwerp, Belgium). ES cells were grown on feeders, trypsinized and electroporated (250 V, 500 μF) with 20 μg of linearized plasmid DNA. Positive selection was started the day after electroporation using G418 at a concentration of 180 μg/ml. Negative selection was started 3 days after electroporation using ganciclovir at a concentration of 2 μM. After 7–8 days of selection, individual ES cell clones were picked and expanded to 96-well plates for freezing and to 24-well plates for genomic DNA preparation. For Southern blot analysis, genomic DNA was isolated from ES cells by standard procedures. Correctly targeted ES cell clones were identified by Southern blot analysis using both 5′ and 3′ probes, cloned by genomic PCR (Fig. 2; Additional file 3: Table S3). Diploid aggregation with morulas of Swiss females, transfer of blastocysts to pseudo-pregnant females and selection of chimeric mice were performed by the TMCF of IRC. Germline transmission of the targeted genes was confirmed by genomic PCR analysis (Additional files 4 and 5: Fig. S3 and Table S4).

### Quantitative RT-PCR (qRT-PCR)

 Total RNA was isolated from mouse tissues using the RNeasy plus kit (Qiagen) according to the manufacturer’s protocol. cDNA was prepared using the iScript cDNA synthesis kit according to the manufacturer’s instructions (Bio-Rad). Quantitative RT-PCR mixes contained 14.7 ng template cDNA, LightCycler 480 SYBR Green I Mastermix (Roche) and 300 nM forward and reverse primers (Additional file 6: Table S5). Reactions were performed on a LightCycler 480 (Roche) using the following protocol: 95 °C for 5 min, 45 cycles at 95 °C for 10 s, 60 °C for 30 s, and 72 °C for 1 s. For normalization, the expression of nine mouse housekeeping genes was analyzed in several mouse tissues (brain, liver, kidney). As determined by geNorm [61], the mouse genes *Gapdh*, *Hprt*, *Rpl13a*, *Sdha* and *Ubc* showed a good expression stability in the three tissues tested and were used for normalization of all mouse tissues. Isoform-specific primer sets to amplify either all, only the short or only the long Pcdh10 isoforms were selected based on both specificity (as determined by melting curve analysis) and efficiency (as determined by a standard curve). For the expression analysis of other mouse Pcdh mRNAs, primers amplifying all isoforms were used.

### Protein expression analysis

Primary antibodies used for western blotting, Immunohistochemistry (IHC) and Immunofluorescence (IF) are listed in Additional files 7 and 8 (Tables S6 and S7). Western blotting was by standard procedures. Brains were crunched in liquid nitrogen and tissue powder was lysed in 1x Laemmli buffer. After sonication, protein concentration was determined with the DC protein assay according to the manufacturer’s instructions (Bio-Rad). 100 μg of total protein lysate was loaded on a 7% one-dimensional SDS-PAGE gel and the separated proteins were transferred to polyvinylidene fluoride (PVDF) membranes (Millipore). After blocking with 5% non-fat dry milk in Tris buffered saline containing 0.1% Tween-20, membranes were incubated with primary antibodies overnight at 4 °C. After several washing steps in Tris buffered saline, membranes were incubated for 1 h with secondary horseradish peroxidase (HRP)-conjugated antibodies. Detection was performed using the enhanced chemiluminescence (ECL) detection system (Amersham GE healthcare). Membranes were exposed to a Phosphor Image screen for appropriate exposure times and scanned with a Molecular Imager (Bio-Rad). Stripping of membranes before reprobing with different antibodies was by Restore™ PLUS Western blot stripping buffer (Thermo Scientific).

To unmask antigens on paraffin sections, sections were rehydrated and treated with citrate buffer in a Retriever apparatus (PickCell Laboratories, Amsterdam, The Netherlands). The sections were incubated with primary antibodies at 4 °C overnight, followed by incubation with secondary antibodies conjugated with biotin (Agilent, Santa Clara, CA, USA). Avidin-biotin complexes were made (Vector Laboratories, Burlingame, CA, USA), and the signal was detected with diaminobenzidine (Agilent). The NFATc1 and vimentin signals were amplified by using a Tyramide Signal Amplification kit (Akoya Biosciences, Marlborough, MA, USA). For IF, slides were labeled with goat anti-rabbit or anti-mouse IgG (H + L) secondary antibody DyLight488 (dilution 1:1000; Thermo Fisher Scientific, Merelbeke, Belgium). Counterstaining was performed with Hoechst and all slides were mounted with 1% propyl gallate in glycerol before analysis with a Leica SP5 confocal scanning microscope.

### Histology and LacZ staining

Histology was on paraffin sections stained with hematoxylin and eosin following standard procedures. For whole-mount staining of β-galactosidase (LacZ) activity, tissues were fixed for 2 h in 4% paraformaldehyde at room temperature, washed 3 times for 30 min with permeabilization buffer (0.01% sodium deoxycholate, 0.02% Nonidet P-40, 10 mM MgCl_2_, 2.5 mM EGTA, 0.01% BSA in 200 mM phosphate buffer, pH 7.4), and stained overnight on a shaker in the dark. Staining was with X-gal (1 mg/ml), 5 mM potassium ferricyanide, 5 mM potassium ferrocyanide in permeabilization buffer. After washing, specimens were fixed for 3 h in 4% paraformaldehyde, followed by 70% ethanol and mounting in paraffin. Detection of LacZ activity in cell cultures was likewise except for fixation for 5 min in 0.8% paraformaldehyde, 0.2% glutaraldehyde in permeabilization buffer, and staining at 37 °C. Mounting was in Aquatex (Merck).

### Tumor-derivation of cell lines

Pinnal-tumor derived (PTD) cell cultures were obtained by cutting aseptically prewashed pinnal tumors into small pieces in PBS with gentamycin (250 μg/ml). Uniform cell suspensions were obtained by treatment at room temperature with dissociation buffer (DMEM, 10% fetal calf serum, 250 μg/ml gentamycin, 0.5% glucose, 0.125 U/ml Dispase II (Sigma-Aldrich), 220 U/ml crude Collagenase (Sigma-Aldrich), followed by gentle centrifugation, filtration through cell strainers of 70 μm and 40 μm, and seeding on tissue-culture plastic substrates. The genotypes of the original tumor-bearing mice and the specific treatments are listed in Table 1. Ablation of floxed genes in the PTD cells was confirmed by genomic PCR. More specifically, the PTD7 cell line was derived from a pinnal tumor in a female mouse with GFAP-Cre^tg/+^;Pcdh10^−/−^;p53^−/−^;Rb^−/−^ genotype, killed at 21.5 weeks after birth. The original cell population was subcloned by limiting dilution and colony picking. The PTD25 cell line was a cell population derived from a pinnal tumor in a male mouse with GFAP-Cre^tg/+^;Pcdh10^−/−^;p53^−/−^;Rb^−/−^ genotype, killed at 17 weeks after birth.

**Table 1:** Origin and characteristics of pinnal-tumor derived (PTD) cell lines and rescued derivatives thereof

Mouse tag number	Genotype of original mouse				PTD cell line	Origin	Enrichment	Colonies in soft agar	In vitro invasion	Growth rate of s.c. allograft
	GFAP-Cre	Pcdh10 all	p53	Rb1						
9034	tg/+	**fl/fl**	**fl/fl**	**fl/fl**	PTD4	Pinnal tumor, dissociated overnight	pool	++	+++	quick
~					**PTD7**	~	subclone	++	+++	quick
~					PTD8	~	subclone	++	+++	intermediate
8822	tg/+	**fl/fl**	**fl/fl**	+/+	PTD11	Pinnal tumor, 4 h dissociation	subclone	none	+	slow
~					PTD12	~	subclone	none	+	very slow
~					PTD12AD4	PTD12 allograft	pool	n.d.	n.d.	quick
9135	tg/+	**fl/fl**	**fl/fl**	+/+	PTD17	Pinnal tumor, 1 h dissociation	subclone	none	n.d.	intermediate
~					PTD19	Pinnal tumor, 4 h dissociation	subclone	none	n.d.	intermediate
1729	tg/+	**fl/fl**	**fl/fl**	**fl/fl**	PTD24	Middle part of pinnal tumor, 1 h dissociation	pool	++	n.d.	very quick
~					**PTD25**	Outer part of pinnal tumor, 1 h dissociation	pool	+++	+++	very quick
9988	tg/+	**fl/fl**	**fl/fl**	**fl/+**	PTD26	Pinnal tumor, 2 h dissociation	pool	n.d.	++	intermediate
~					PTD27	Pinnal tumor, 4 h dissociation	pool	n.d.	+	intermediate
**Pcdh10 Rescued Derivatives**										
9034	tg/+	**fl/fl**	**fl/fl**	**fl/fl**	PTD7_RS	PTD7, rescued with Pcdh10 short isoform 1	FACS sorted	none	-(+)	reduced rate
~					PTD7_RL	PTD7, rescued with Pcdh10 long isoform 4	FACS sorted	none	-(+)	reduced rate
1729	tg/+	**fl/fl**	**fl/fl**	**fl/fl**	PTD25_RS	PTD25, rescued with Pcdh10 short isoform 1	FACS sorted	none	–	no tumor
~					PTD25_RL	PTD25, rescued with Pcdh10 long isoform 4	FACS sorted	none	–	reduced rate

### Soft agar colony formation

Anchorage-independent growth in soft agar was measured as described before [62].

### In vitro invasion / migration

Multicellular spheroidswere generated using cells of the PTD and derivative cell populations and embedded in a 3D-collagen Type I hydrogel (1 mg/ml) in 12-well plates as described [63]. Phase contrast images of the embedded spheroids were taken using an Olympus Cell M system at the indicated time points for up to 5 days. Quantification of the sphere perimeters was performed in FIJI* (*https://fiji.sc/*).*

### Allograft formation

Dissociated single-cell suspensions were mixed on ice with an equal volume of Matrigel (Corning, Life Sciences). An inoculum of 200 μl containing 10^3^ to 5 × 10^6^ cells was injected subcutaneously (s.c) into the flank of 3-week old athymic mice (strain Hsd:Athymic Nude-Foxn1^nu^; Envigo, Indianapolis, USA). Four to five animals were injected per cell line and concentration. The human cell lines HOS and MNNG-HOS [62] served as negative and positive controls, respectively. Starting from d 5 after injection, tumor dimensions were measured regularly with callipers, and volumes calculated using the formula: (π/6) x L x W x H.

### Lentiviral rescue of Pcdh10 expression

To obtain derivative cell lines of PTD7 and PTD25, rescued for the ablation of Pcdh10 by the introduction and expression of cDNA encoding either long isoform 4 of mouse Pcdh10 (designated _RL) or short isoform 1 of mouse Pcdh10 (designated _RS), the parental cell lines were transduced with recombinant lentiviruses. To this end, we constructed plasmids pLenti6(bla)-mPcdh10short (no stop)-HA (encoding short isoform 1 of mouse Pcdh10) and pLenti6(bla)-mPcdh10-long (no stop)-HA (encoding long isoform 4 of mouse Pcdh10), both available at the BCCM/GeneCorner Plasmid Collection (Ghent University, Ghent, Belgium) as plasmids LMBP12686 and LMBP12687, respectively. HEK293T cells (1.2 × 10^6^) were seeded in a 25-cm^2^ flask and treated with 25 μM chloroquine for 5 min. The cells were then co-transfected with each of the abovementioned lentiviral vectors (3 μg), together with pCMV D8.9 packaging plasmid (3 μg) and pMD.G envelope plasmid (1.5 μg), using the calcium phosphate method. After 8 h the cells were washed, and viral supernatant was harvested after 48 h. PTD7 and PTD25 cells were transduced by spinoculation (12-well plate, 3200 rpm, 90 min, 32 °C) with the filtered (45 μm) viral stocks, followed by blasticidin selection (7 μg/ml).

The transduced cell populations were enriched for viable Pcdh10 expressing cells by FACS (VIB Flow Core Facility). For this experiment, single-cell suspension were obtained by non-enzymatic cell dissociation solution (Sigma-Aldrich), containing 1% BSA and 20% fetal calf serum. The primary antibody used was a rabbit polyclonal antibody recognizing epitope AA 1–290 of human and mouse Pcdh10 (Invitrogen Cat. No. PA5–31043). This epitope is exposed extracellularly. The second antibody was polyclonal Alexa Fluor 488 goat anti-rabbit antibody (Sigma-Aldrich). Dead cells were excluded on the basis of DAPI staining. Expression of Pcdh10 cDNA in the rescued cell lines was confirmed by immunofluorescent microscopy.

### RNA-Seq analysis

All cell lines to be analyzed by RNA-Seq were grown to confluence in 75-cm^2^ tissue culture flasks before RNA extraction. For each cell line three to four biological replicates were processed. Total RNA was extracted with TRIZOL reagent (Life Technologies), followed by on column extraction with the Aurum Total RNA mini kit (Life Science Research – Bio-Rad). Concentrations were measured by the NanoDrop technology (Thermo Fisher Scientific) and adjusted to 300 ng/μl. RNA quality was assessed by the Bioanalyzer RNA 6000 Nano assay (Agilent, Santa Clara, CA, USA) and found to be highly intact for all samples (RNA Integrity Number of 9.9 to 10). RNA-Seq library preparation was performed by the VIB Nucleomics Core facility (Leuven, Belgium), using the Illumina TruSeq stranded mRNA prep kit (Illumina Inc., San Diego, CA, USA). Samples were sequenced by the Illumina NextSeq 500 High 75 Sequencing System, yielding single-end 75-bp reads. The preprocessing of the RNA sequencing data was done by Trimmomatic v0.35. The adapters were cut off, and reads were trimmed when the quality dropped below 20. Reads with a length < 35 were discarded. All samples passed quality control based on the results of FastQC v0.11.4. Reads were mapped to the mouse reference genome (mm10) via Tophat2 v2.1.0, sorted using samtools v0.1.18 and counted via HTSeqCount v0.6.1p1. Samples were subsequently analyzed using R/Bioconductor, and the DESeq2 procedure was used to normalize the data and to calculate the differentially expressed (DE) genes. The datasets generated during this study have been deposited in the Gene Expression Omnibus public database under accession number GEO: GSE198236.

### Ingenuity pathway analysis (IPA)

Datasets of DE genes were assembled as follows (Additional files 9, 10, 11: Tables S8-S10). For the PTD7 cell family, genes were considered differentially expressed when the Benjamini-Hochberg adjusted *p*-value (padj) was < 0.01, and when the log2FoldChange was either < − 1 or > 1. For the PTD25 cell family and for the comparison PTD25 versus PTD7, genes were considered differentially expressed when padj was < 0.01, and when the log2FoldChange was either < − 2 or > 2. DE genes, that were either shared by Pcdh10 rescued cell lines, or that were unique to a particular cell line, were manually selected from the abovementioned datasets. The datasets were uploaded in the QIAGEN IPA program, using the values of updated gene ID, baseMean (mean of normalized counts for all samples of each comparison), log2FoldChange, p_adj_ (Benjamini-Hochberg adjusted Wald test p-value). Core analyses were run on the various dataset files, on the basis of the Expression Log Ratios, using the Ingenuity Knowledge Base (Genes only) as reference set. Direct and indirect relationships were considered, and all available data sources were used except for COSMIC and ClinVar. No cutoff values were applied. The number of networks was restricted to 25, each comprising a maximum of 35 DE genes. The subcellular layout was chosen for both graphical summaries and separate network displays.

## Results

### Conditional knockout of Pcdh10 isoforms

We have generated two conditional knockout mouse models, deleting either all or only the long isoforms of Pcdh10 (Pcdh10all^fl/fl^ and Pcdh10long^fl/fl^, respectively) (Fig. 2). For mouse Pcdh10 four transcripts have been identified (Fig. 1A). All four contain genetic information of exon 1, encoding the extracellular domain of Pcdh10, its transmembrane domain and a 138-AA part of the cytoplasmic domain. The short isoform is entirely encoded by an extended version of exon 1, yielding a 896-AA protein with a cytoplasmic tail lacking the conserved motifs CM1 and CM2, which are encoded by, respectively, exon 3 and 4. The three long isoforms in the mouse (1040-, 1047- and 1057-AA) are generated by splicing of exon 1 to exon 2, followed by exons 3 and 4, and by, respectively, the alternatively used exons 5, 5.1 or 5.2. In the mouse, this results in the translation of 4 proteins, differing only at their carboxyterminal end (Fig. 1B). In Pcdh10all^fl/fl^ mice the long exon 1 was floxed (Fig. 2A) and Cre recombinase activity in such mice will therefore ablate all 4 isoforms of Pcdh10. In contrast, in Pcdh10long^fl/fl^ mice exons 2 and 3 are floxed (Fig. 2B), and Cre-mediated deletion in these mice will result in a frameshift in exons 4 and 5, so that only the shortest isoform 1 is expected to be expressed.

**Fig. 3:**
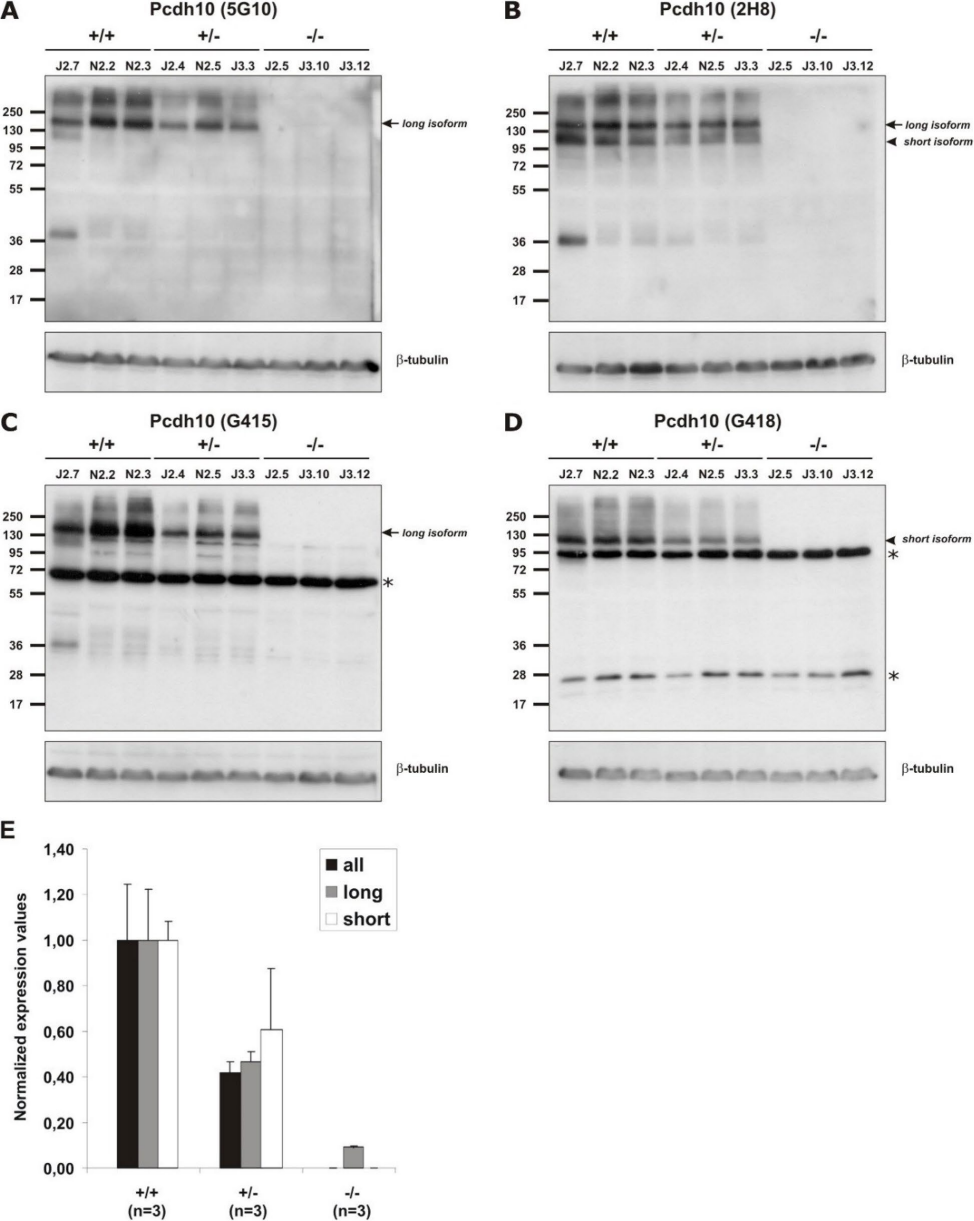
Pcdh10 expression levels in Pcdh10all^−/−^ mice. **A-D** Western blot analysis of brain lysates from wild-type (+/+), heterozygous (+/−) and homozygous (−/−) mutant Pcdh10all mice. See Additional file 8: Table S7 for antibody information. Loading control: β-tubulin. The corresponding original, uncropped Western blots are shown in Additional files 26 to 28. **A** Monoclonal antibody 5G10 detects only long isoforms. **B** Monoclonal antibody 2H8 detects all isoforms. **C** Polyclonal antibody G415 detects only long isoforms. **D** Polyclonal antibody G418 detects only the short isoform. Arrows point to the band corresponding to the long isoforms, arrowheads point to the band corresponding to the short isoform. Asterisks indicate aspecific bands recognized by the polyclonal antibodies. **E** qRT-PCR analysis of brains from wild-type, heterozygous and homozygous mutant Pcdh10all mice. Expression levels of all (black bars), long (grey bars) and short (white bars) isoforms were determined in 3 mouse brains of each genotype and data are represented as Mean + SD. Values for wild-type brains were set to 1. See Additional file 6: Table S5 for primer information

**Fig. 4:**
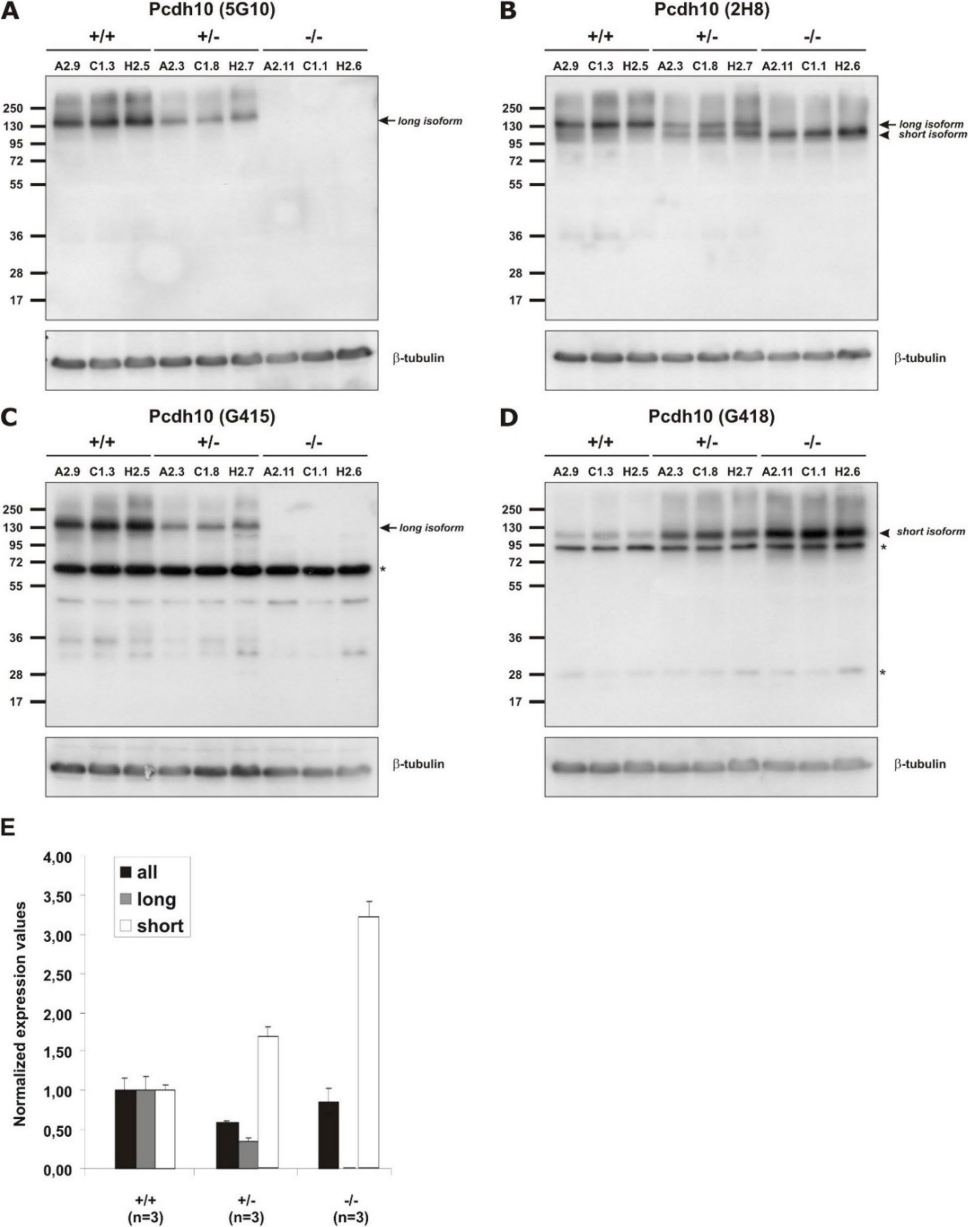
Pcdh10 expression levels in Pcdh10long^−/−^ mice. **A-D** Western blot analysis of brain lysates from wild-type (+/+), heterozygous (+/−) and homozygous (−/−) mutant Pcdh10long mice. See Additional file 8: Table S7 for antibody information. Loading control: β-tubulin. The corresponding original, uncropped Western blots are shown in Additional files 29 to 32. **A** Monoclonal antibody 5G10 detects only long isoforms. **B** Monoclonal antibody 2H8 detects all isoforms. **C** Polyclonal antibody G415 detects only long isoforms. **D** Polyclonal antibody G418 detects only the short isoform. Arrows point to the band corresponding to the long isoforms, arrowheads point to the band corresponding to the short isoform. Asterisks indicate aspecific bands for the polyclonal antibodies. **E** qRT-PCR analysis of brains from wild-type, heterozygous and homozygous mutant Pcdh10long mice. Expression levels of all (black bars), long (grey bars) and short (white bars) isoforms were determined in 3 mouse brains of each genotype and data are represented as Mean + SD. Values for wild-type brains were set to 1. See Additional file 6: Table S5 for primer information

The correct genotype of these animals was checked at the genomic (Additional file 4: Fig. S3), transcriptional and protein levels. Both qRT-PCR and western blot analyses, using either broad-specificity or isoform-specific antibodies, confirmed the complete loss of all Pcdh10 isoforms in the brains of Pcdh10all-null mice (Fig. 3). These knock-out (KO) mice were obtained by crossing Pcdh10all^fl/fl^ with a transgenic mouse line showing ubiquitous expression of Cre recombinase in all adult tissues (Nestin-Cre) [56]. We extended the qRT-PCR analysis to several related δ-Pcdhs, and this revealed upregulated transcript levels for several of them, pointing at a compensatory mechanism (Additional file 12: Fig. S4A). The situation was different in the brains of Pcdh10long-null mice, which were obtained by crossing Pcdh10long^fl/fl^ mice with the Nestin-Cre mouse. The qRT-PCR analysis did not show upregulation of other δ-Pcdhs (Additional file 12: Fig. S4B), while both qRT-PCR and the western blot analyses revealed that progressive loss of long isoforms was compensated by transcriptional upregulation of the short isoform (Fig. 4).

Uemura et al. [49] reported severe defects in axon pathways in the ventral telencephalon of Pcdh10 null mice, smaller size at birth and death within several weeks after birth. Surprisingly, our homozygous Pcdh10 KO mice, affecting either all or only the long isoforms of Pcdh10, did not show any prominent phenotype or early lethality (Additional file 13: Fig. S5). Birth rate of heterozygous and homozygous mutant animals was in accordance with Mendelian ratios. Body weight of wild-type, heterozygous and homozygous KO animals was recorded for 8 weeks after birth and showed no significant differences between genotypes (Additional file 14: Fig. S6). Signs of neurological defects, early lethality or decreased fertility were not observed in our mutant animals.

### GFAP-Cre mediated knockout of Pcdh10 isoforms aggravates medulloblastoma and auricular tumor formation in transgenic mouse models

**Fig. 5:**
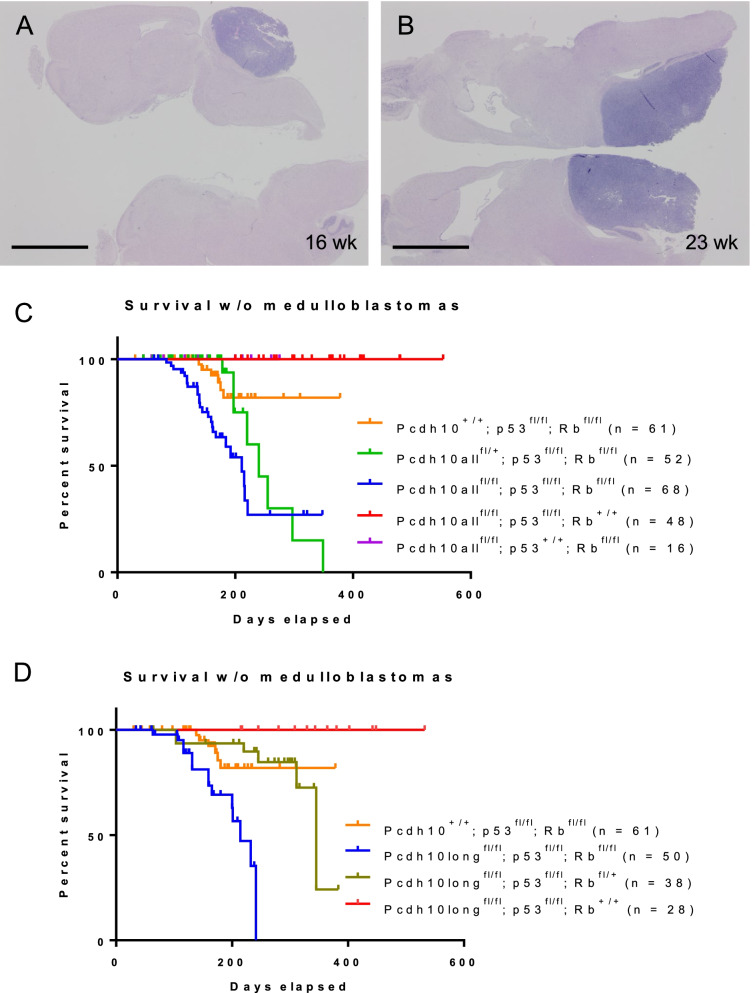
Development of medulloblastoma in GFAP-Cre^tg/+^ transgenic mice. **A,B** Sagittal sections through the brain of GFAP-Cre^tg/+^;Pcdh10all^fl/fl^;p53^fl/fl^;Rb^fl/fl^ mice at, respectively, 16 and 23 weeks after birth. The normal cerebellum is progressively replaced by medulloblastoma. Scale bars: 5 mm. **C,D** Evolution of medulloblastoma-free mice with the genotypes indicated. The number of mice evaluated is indicated between brackets. Pathological evaluation was initially on the basis of typical behavioral abnormalities (tremor, uncoordinated movements, balance distortions), but confirmed for each case by dissection and histology. See Additional file 15: Table S11 for statistical analysis

As PCDH10 expression is frequently repressed in human medulloblastoma [11], which is originating in the external granular layer of the developing cerebellum, we checked the effect of Pcdh10 ablation in an established mouse model for medulloblastoma [54]. In this model, floxed *Trp53* and *Rb1* alleles are recombined by a Cre recombinase under control of the *GFAP* (glial fibrillary acidic protein) promoter. We have modified this genotype by crossing in either the Pcdh10all^fl^ or the Pcdh10long^fl^ alleles. Kaplan-Meyer curves of medulloblastoma-free survival revealed: i. strict dependence of medulloblastoma formation on both Rb ablation and p53 ablation (Fig. 5), in concordance with published data [54]; ii. shorter tumor lag period and increased fraction of animals affected in case of heterozygous and in particular homozygous ablation of all Pcdh10 isoforms (Fig. 5C). All curve differences were statistically significant (Additional file 15: Table S11). Also in case of ablation of only long Pcdh10 isoforms, a statistically significant decrease in tumor-free survival was observed (Fig. 5D).

**Fig. 6:**
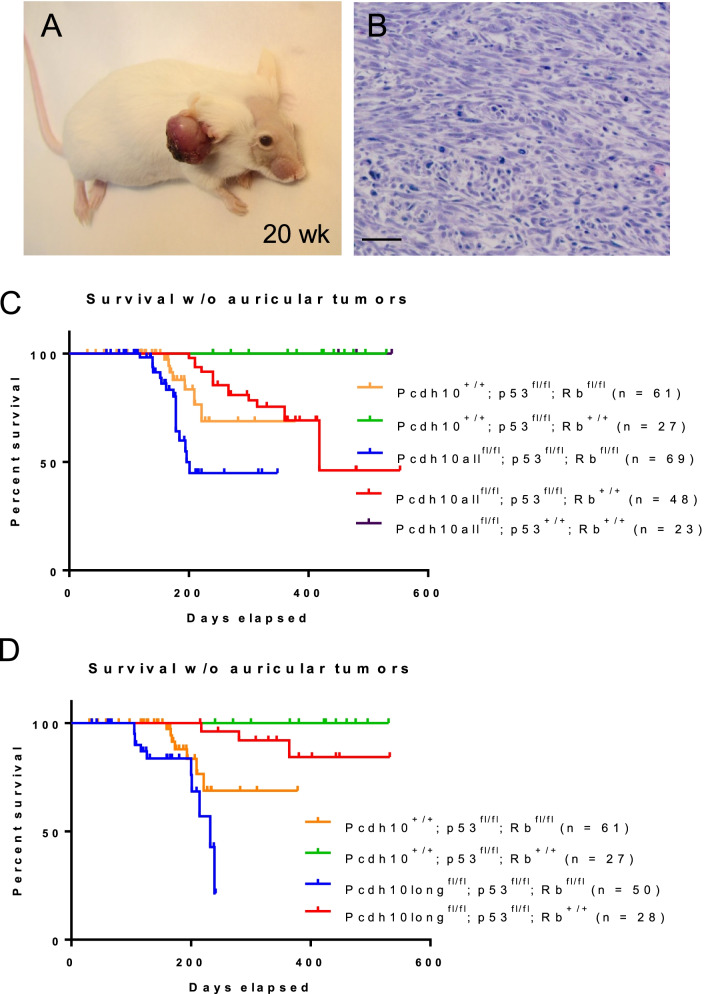
Development of auricular/pinnal tumors in GFAP-Cre^tg/+^ transgenic mice. **A** Auricular tumor in a GFAP-Cre^tg/+^;Pcdh10all^fl/fl^;p53^fl/fl^;Rb^fl/fl^ mouse at 20 weeks after birth. These pinnal tumors arise only unilaterally in the pinna marked with a monel tag. **B** Typical histology of a pinnal tumor. Scale bar: 50 μm. **C,D** Evolution of auricular-tumor free mice with the genotypes indicated. The number of mice evaluated is indicated between brackets. See Additional file 15: Table S11 for statistical analysis

Unexpectedly, auricular (or pinnal) tumor formation was observed in these same animals (Fig. 6). The occurrence of these tumors was dependent on several factors: i. in all cases, auricular tumor formation occurred only unilaterally, namely exclusively in the ear with monel number tag, indicating that chronic irritation or inflammation is a requisite for this type of tumorigenesis (Fig. 6A); ii. in case of WT Pcdh10 (Pcdh10^+/+^), tumor formation was dependent on GFAP-Cre driven Rb ablation; iii. in case of Pcdh10all ablation (Fig. 6C), tumor formation was significantly more efficient than in case of WT Pcdh10; iv. contrary to medulloblastoma formation, auricular tumor formation did occur in a WT Rb background upon ablation of both Pcdh10 (all isoforms) and p53 (Fig. 6C); v. similar findings were done for GFAP-Cre driven Pcdh10long ablation (Fig. 6D), although in these mice differences between curves did not reach statistical significance (Additional file 15: Table S11). Histopathological examination of these lesions suggested a fibrosarcomatous tumor type in most cases (Fig. 6B).

### GFAP-Cre induced auricular tumors show stem cell features in vivo

**Fig. 7:**
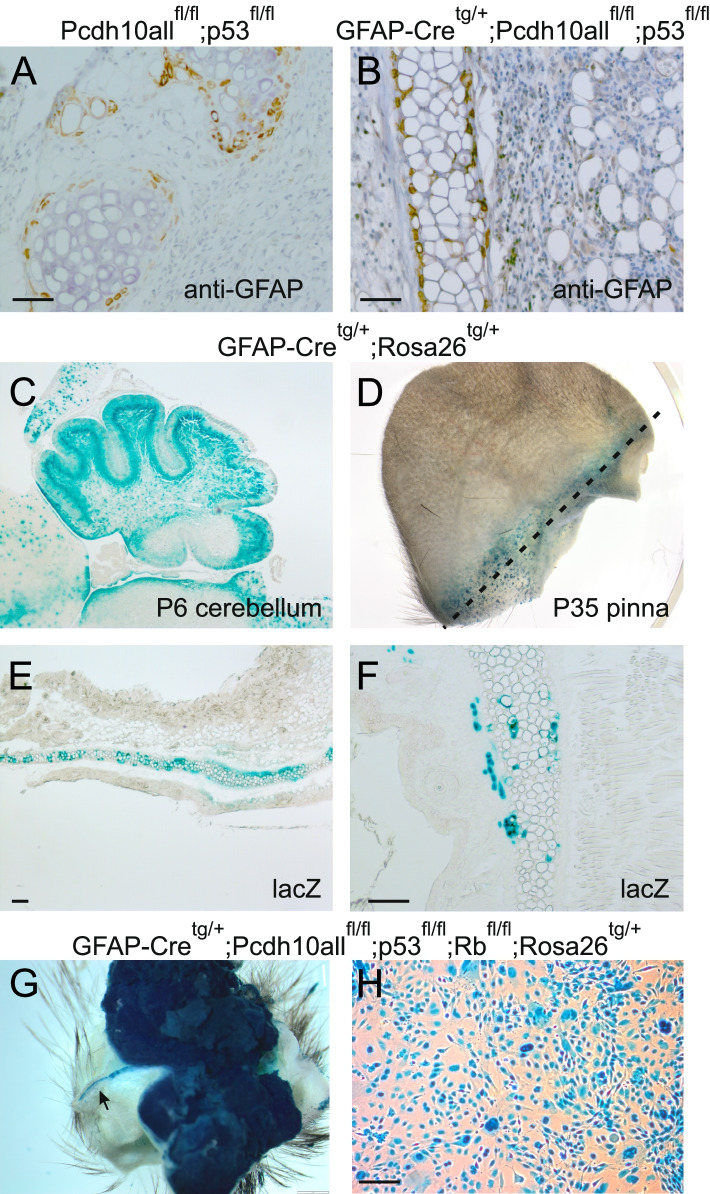
Detection of GFAP protein and GFAP-Cre activity in mouse tissues and tumors. Genotypes of mice analyzed are given on top of the corresponding panels. **A,B** Immunostaining of pinnal tissues with anti-GFAP antibody. In normal pinnae (**A**), only perichondrial stem cells (PSC) are positive. In case of pinnal tumors (**B**), also tumor cells surrounding the elastic cartilage may be positive, but this is often not the case. Scale bars: 50 μm. **C,D** GFAP-Cre activity in GFAP-Cre^tg/+^;Rosa26^tg/+^ mice, as detected by LacZ staining. **C** Positive staining of the granular layer of the cerebellum (young P6 mouse). Sagittal section obtained after whole-mount LacZ staining of the brain. **D** Positive whole-mount staining at the basis of the pinna (P35 mouse). The broken line indicates the section plane, yielding sections shown in **E** and **F**. Scale bars: 100 μm. LacZ-positive cells were seen at the location of PSC. **G** Strong LacZ activity in a pinnal tumor demonstrates previous GFAP-Cre activity. The arrow points at positivity in ‘normal’ elastic cartilage. **H** Strong LacZ activity in a cell line derived from a tumor, similar to this depicted in **G**. Scale bar: 50 μm

Considering that medulloblastomas are known to arise in cerebellar stem cells [54, 64], we wondered about the cells of origin of the auricular tumors. We checked the location of GFAP-Cre activity in two ways: i. immunolocalization of GFAP protein; ii. lacZ activation in a Cre reporter mouse. The latter mouse was obtained by crossing GFAP-Cre mice with the R26R reporter mouse, which carries a loxP-STOP-loxP-*lacZ* cassette targeted into the ubiquitously expressed *ROSA26* locus [58]. Consistent results were obtained: GFAP was specifically detected in the perichondrium of the elastic cartilage of auricles, but neither in the mature chondrium nor in other tissues of the auricles (Fig. 7A,B). This particular perichondrium has been reported to harbour presumptive somatic stem cells, perichondrial stem cells (PSC) [65], and to be derived from the 1st branchial arch [66]. In agreement with this, prominent GFAP-Cre driven LacZ activity was seen in the perichondrium of adult GFAP-Cre^tg/+^;Rosa26^tg/+^ mice, but only rarely in mature chondrocytes and not at all in other tissues of the auricles (Fig. 7D-F). As expected, brain structures and in particular the cerebellum were positive for lacZ in this GFAP-Cre reporter mouse (Fig. 7C).

Unfortunately, we were unable to identify reliable anti-Pcdh10 antibodies, suitable for immunohistochemical analysis in the mouse. However, the causality between auricular tumor formation and GFAP-Cre mediated Pcdh10 ablation was further strengthened in the following ways: i. both auricular and cerebellar tumors in mice with GFAP-Cre^tg/+^;Pcdh10^fl/fl^;p53^fl/fl^ genotypes showed consistent Pcdh10 ablation (either all or only long isoforms) when analyzed by genomic PCR; ii. in an R26R reporter mouse with GFAP-Cre^tg/+^;Pcdh10all^fl/fl^;p53^fl/fl^;Rb^fl/fl^ genotype both the site of pinnal elastic cartilage and the whole pinnal tumor were lacZ-positive (Fig. 7G); two cell lines, which were obtained from such pinnal tumors, expressed lacZ homogeneously in vitro (Fig. 7H), but not Pcdh10; iii. in line with this, several cell lines were derived from pinnal tumors, and these completely lacked expression of Pcdh10, while some other protocadherins were nonetheless expressed (see below).

**Fig. 8:**
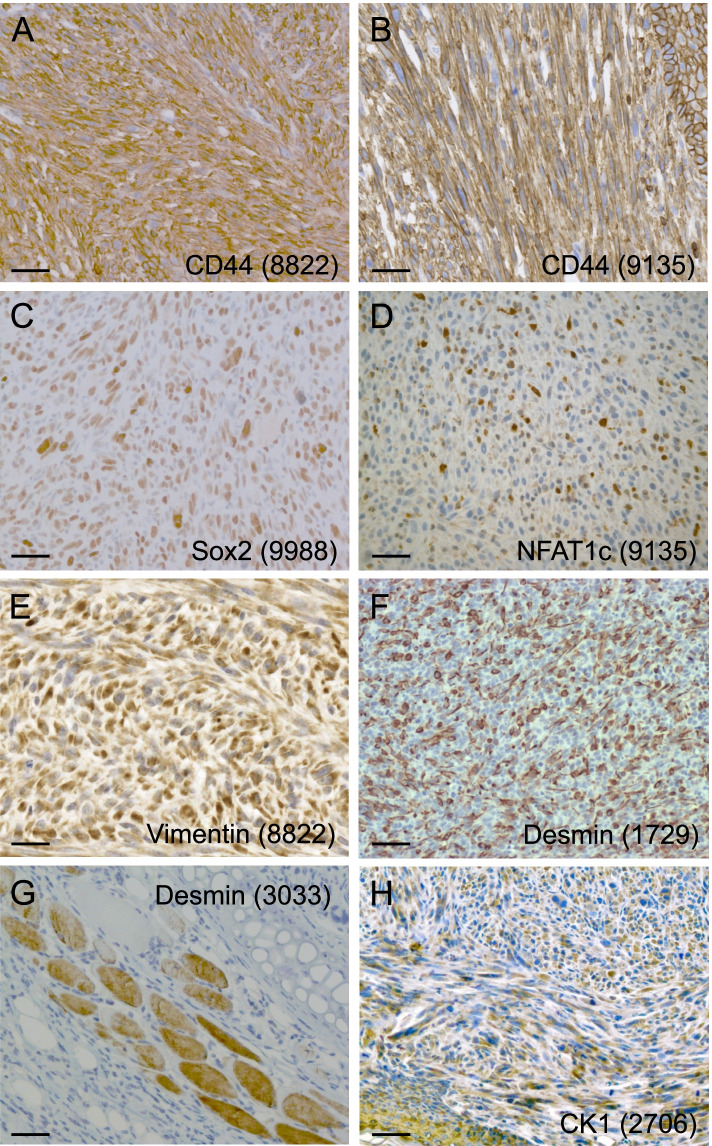
Immunohistochemical detection of stem cell markers, inflammation marker, and intermediate filaments in representative pinnal tumors. **A, B** cytoplasmic and cell surface CD44 staining (Upper right corner in **B**: normal epidermis with cell contact staining). **C** Nuclear Sox2 staining. **D** Nuclear NFAT1c staining. **E** Cytoplasmic vimentin staining. **F** Strong cytoplasmic desmin staining in one pinnal tumor. **G** Desmin staining in normal skeletal muscle of the pinna. **H** Cytoplasmic cytokeratin-1 staining (normal epidermis in the lower left corner). The genotype of mice in (**A,B,D,E,G,H**) is GFAP-Cre^tg/+^;Pcdh10all^fl/fl^;p53^fl/fl^;Rb^+/+^. The mouse in (**C**) has genotype GFAP-Cre^tg/+^; Pcdh10all^fl/fl^;p53^fl/fl^;Rb^fl/+^.The mouse in (**F**) has genotype GFAP-Cre^tg/+^;Pcdh10all^fl/fl^;p53^fl/fl^; Rb^fl/fl^. Mouse ear tag numbers are given between brackets. All scale bars: 50 μm

Although we cannot exclude that also other cell types besides PSC contribute to auricular tumor formation, we sought further evidence for a stem cell origin by analyzing these peculiar auricular tumors in more detail. We performed immunohistochemistry for several markers. Specificity of the antibodies for their respective antigens in the specimens under study was carefully validated as exemplified in Additional file 16: Fig. S7. CD44 has been reported to be expressed in the PSC of the pinna [65], and found by us to be generally expressed also in the pinnal tumors (Fig. 8A,B; Additional file 16: Fig. S7G and H). Sox2, which is a more universal stem cell marker, was not detected in the PSC but several pinnal tumors showed moderate to frequent expression of nuclear Sox2 (Fig. 8C; Additional file 16: Fig. S7F). Interestingly, NFATc1, a general marker of inflammation, was detected in numerous nuclei of several pinnal tumors (Fig. 8D; Additional file 16: Fig. S7C). In line with a fibrosarcoma histology, several pinnal tumors were positive for vimentin (Fig. 8E), but this staining was often heterogeneous and about half of the tumors was essentially negative for vimentin (Additional file 16: Fig. S7D). In one tumor (mouse #1729), we detected consistent desmin expression by the tumor cells (Fig. 8F), while other tumor-bearing pinnae showed specific desmin expression in normal skeletal muscle only (Fig. 8G). Also surprisingly, many tumors stained positively for cytokeratins (illustrated for CK1 in Fig. 8H). This prompted us to check also typical epithelial junctional proteins: E-cadherin and the associated beta-catenin and p120ctn (Additional file 17: Fig. S8; Additional file 16: Fig. S7E). E-cadherin was often weakly expressed, but mainly atypically in the cytoplasm. Beta-catenin was detected in many tumor cells, but generally cytoplasmic or nuclear, in line with activation of the Wnt signalling pathway. Also p120ctn was often strongly expressed in the cytoplasm, sometimes even in the nuclei. Expression of beta-catenin at cell-cell contacts was seen in the tumor with desmin positivity (mouse #1729). In view of these findings, we speculate that PSCs of the pinna have been subject to inflammation-triggered tumorigenesis. Tumors have apparently undergone progression along several ways of incomplete differentiation resulting in diverse features of fibrosarcoma, sarcomatoid (spindle-cell) carcinoma and even rhabdosarcoma.

### GFAP-Cre induced auricular tumors show stem cell features ex vivo

**Fig. 9:**
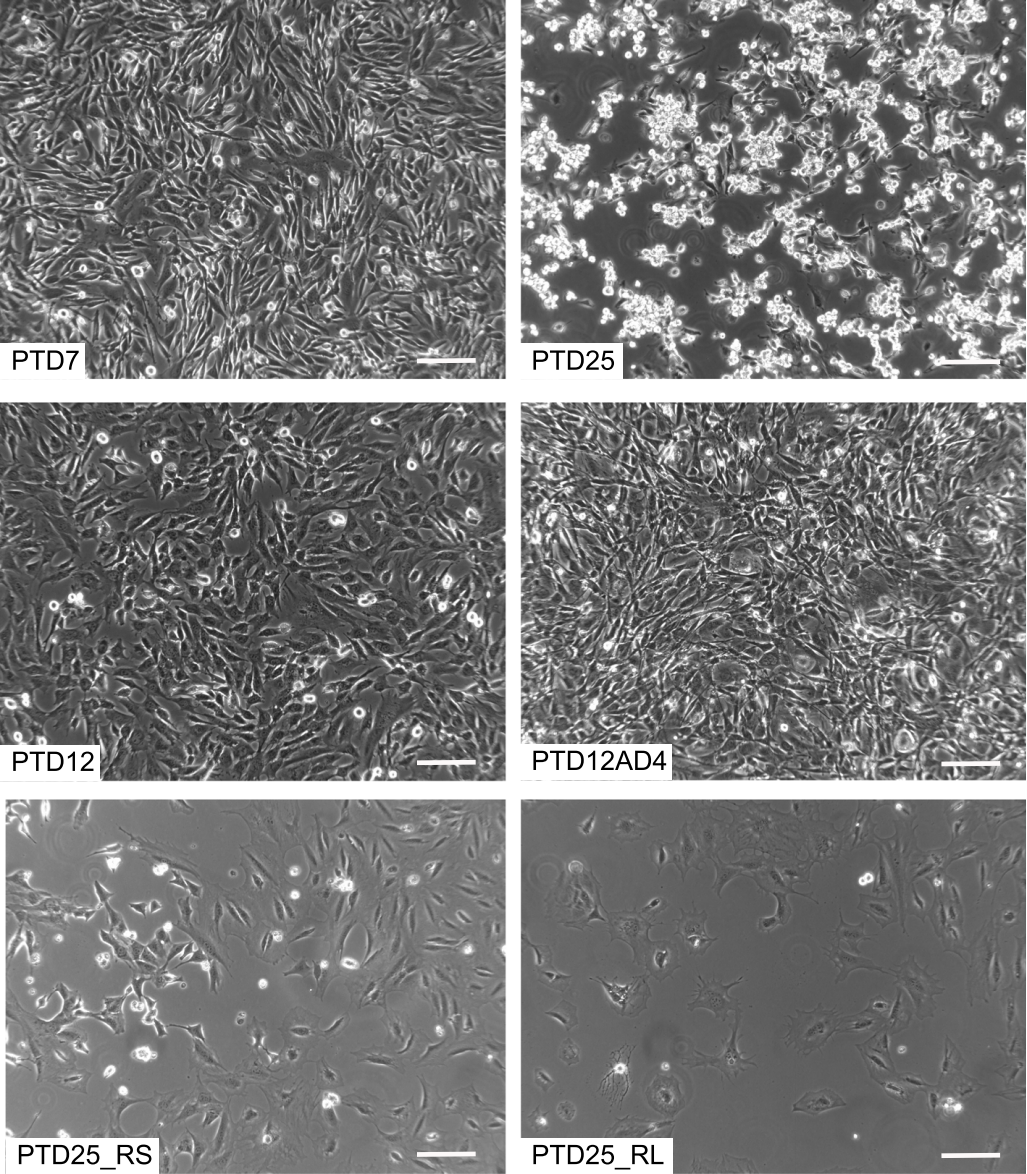
Phase contrast microscopy of pinnal-tumor derived cell lines (PTDs) and derivatives. Genotypes of the original pinnal-tumor bearing mice (Table 1) were: GFAP-Cre^tg/+^;Pcdh10all^fl/fl^;p53^fl/fl^;Rb^fl/fl^ (for PTD7 and PTD25) and GFAP-Cre^tg/+^;Pcdh10all^fl/fl^;p53^fl/fl^;Rb^+/+^ (for PTD12). PTD12AD4 is a cell line derived from an allograft generated by s.c. injection of 2.5 × 10^6^ cells PTD12 (see text and Additional file 19: Fig. S9). PTD25_RS and PTD25_RL are transduced and FACS-sorted derivative populations of PTD25, rescued for Pcdh10 ablation by cDNA expression of, respectively, Pcdh10_short isoform 1 and Pcdh10_long isoform-4 (see text, Table 1 and Additional file 18: Table S12). Scale bars: 50 μm

**Fig. 10:**
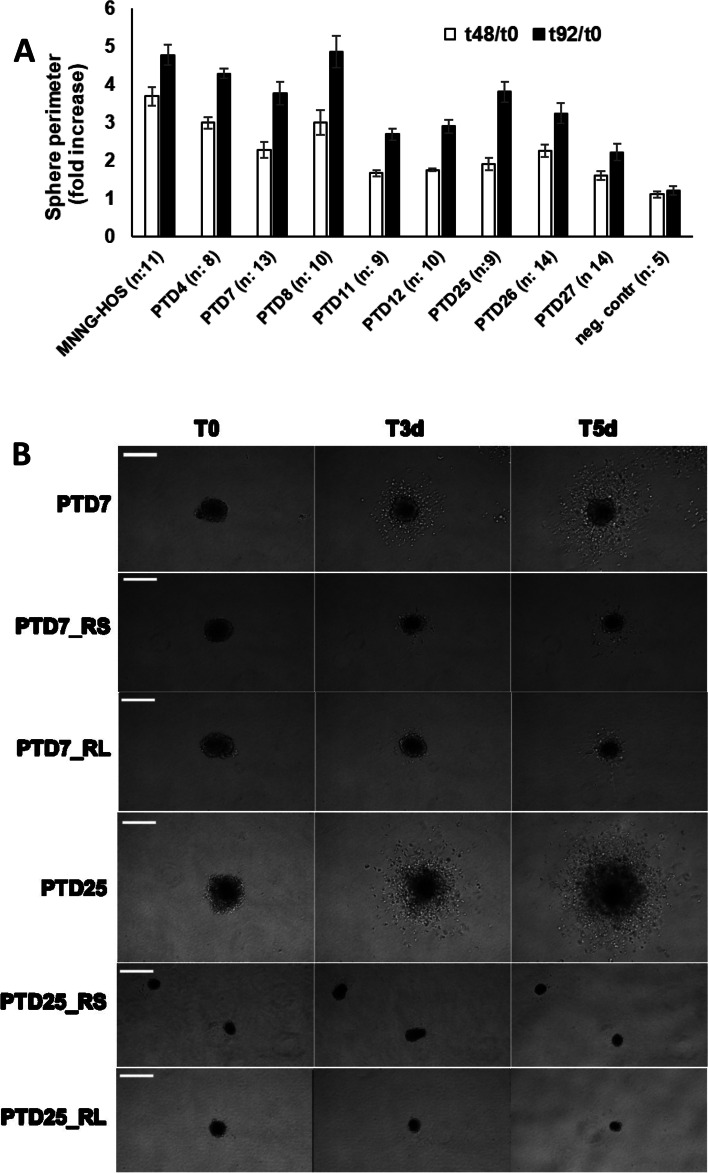
Invasive growth in 3D collagen matrices by spheroids generated from PTD cell lines. **A** Quantitation of representative experiments is shown. For details on PTDs see Table 1. Genotypes of the original pinnal-tumor bearing mice were: GFAP-Cre^tg/+^;Pcdh10all^fl/fl^;p53^fl/fl^;Rb^fl/fl^ (for PTD4, 7, 8, 25), GFAP-Cre^tg/+^;Pcdh10all^fl/fl^;p53^fl/fl^;Rb^fl/+^ (for PTD26, 27) and GFAP-Cre^tg/+^;Pcdh10all^fl/fl^;p53^fl/fl^;Rb^+/+^ (for PTD11, 12). Sphere perimeters at 48 and 92 h were normalized to the sphere perimeter at t0 (fold increase). MNNG-HOS is a human cancer cell line, which served as a positive control [62]; The negative control is a non-invasive human breast cancer cell line T47D (sphere diameter fold increase ~ 1). For each cell line, the number of spheroids observed is given between brackets, and the values plotted are the means + SEM. **B** Representative data for three time points are shown, up to 5 days after seeding (T5d). PTD7 and PTD25 are the original PTD cell lines obtained from mice with GFAP-Cre^tg/+^;Pcdh10all^fl/fl^;p53^fl/fl^;Rb^fl/fl^ genotype. PTD7_RS and PTD25_RS are transduced and FACS-sorted derivative cell populations of, respectively, PTD7 and PTD25, rescued for Pcdh10 ablation by cDNA expression of the Pcdh10_short isoform 1 (see text, Table 1 and Additional file 18: Table S12). PTD7_RL and PTD25_RL are similar derivative cell populations, but rescued by cDNA expression of the Pcdh10_long isoform-4. Note the strongly reduced invasion or even lack of invasion upon Pcdh10 rescue. Scale bars, 150 μm

**Fig. 11:**
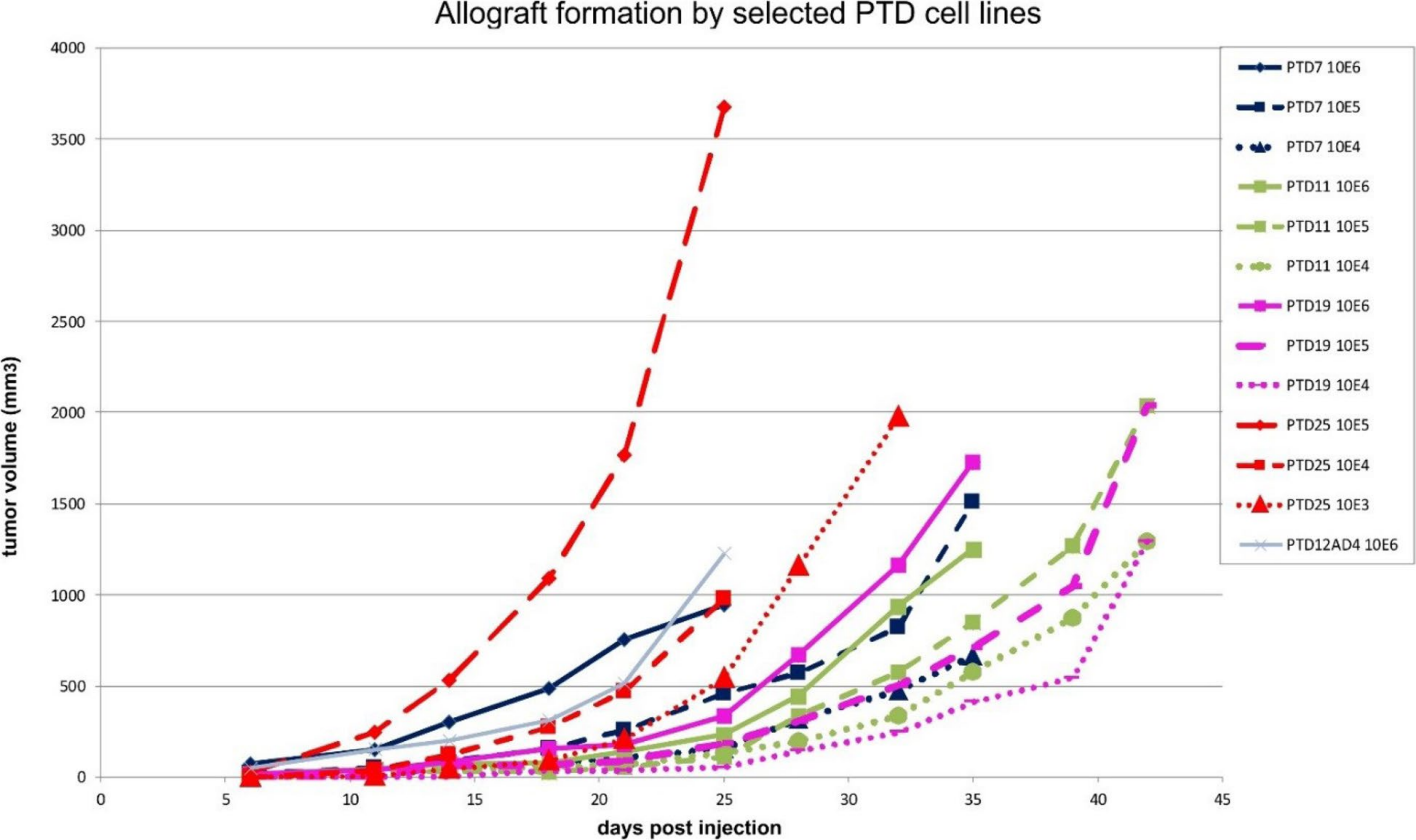
Allograft formation by decreasing amounts of various PTD cell lines. Athymic nude mice were s.c. injected. The number of inoculated cells varied from 10^6^ to 10^3^ as indicated for the PTD lines analyzed. PTD12AD4 is a cell line derived from an allograft generated by s.c. injection of 2.5 × 10^6^ cells PTD12 (see text and Additional file 19: Fig. S9). The cells were mixed with Matrigel before injection. Tumor volumes are the average for 5 injected mice. See Additional file 18: Table S12 for quantitation

To further our insight in the characteristics of the pinnal tumors, we explanted several of them into culture. These pinnal-tumor derived (PTD) cell lines were generally composed of elongated cells, which proliferated rapidly in vitro. Morphological heterogeneity in the cultures was reduced by subcloning. At the end, we performed analyses on 11 PTD lines, derived from 5 original tumors and fully checked for correct genotype by PCR (Table 1; Additional file 18: Table S12). We analyzed these cell lines for morphology, for anchorage-independent growth in soft agar, for invasive growth in extracellular matrix, and for subcutaneous (s.c.) allograft formation in athymic animals. Morphology was generally elongated and fibroblastic, except for PTD25 cells, which were highly refractile and much less adherent (Fig. 9). Colony formation in soft agar was clearly dependent on Rb ablation. All PTD cell lines displayed strong invasive capacity in a 3D-collagen matrix in vitro. This was based on the observation of single cells escaping from multicellular spheroids and showing invasive growth (Fig. 10). S.c. allografts were formed by each of the PTDs, although with diverse growth rates and lag periods (Additional file 19: Fig. S9). PTDs from mice with wild-type Rb (PTD11, 12, 17, 19) were slower performers. Those with confirmed ablation of Pcdh10all, p53 and Rb (PTD4, 7, 8, 24, 25) were remarkably efficient allograft formers. The increased aggressiveness in function of Rb ablation was also apparent from experimental metastasis assays. Injections of 2 × 10^5^ viable cells into the tail veins of athymic mice yielded fast growing lung colonies for PTD7 and PTD25, but none for either PTD11 or PTD19 cells (Additional file 20: Table S13 and Fig. S10). However, when a PTD12 allograft with wild-type Rb and showing a very long lag period was put in culture, yielding PTD12AD4, the latter formed allografts much more efficiently (Fig. 11 and Additional file 18: Table S12). Moreover, we repeated the allograft experiment for four PTD lines using decreasing numbers of inoculated cells (Fig. 11; Additional file 18: Table S12), and found that even inocula as small as 10^4^ or 10^3^ cells readily formed tumors in case of PTD25. PTD 25 was derived from the pinnal tumor in mouse #1729, and shows desmin positivity like the original tumor (see Fig. 8F and Additional file 21: Fig. S11). These features are in line with the presence of cancer stem cells in the PTD populations [67].

### Restoration of Pcdh10 expression in two PTD cell lines strikingly reduces malignancy

**Fig. 12:**
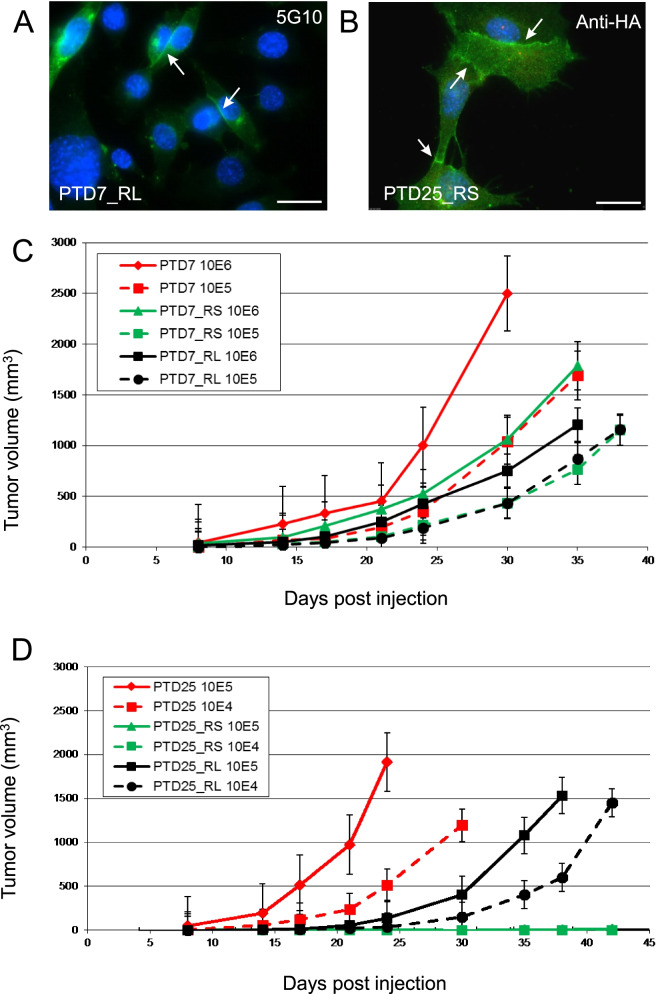
Restored expression of Pcdh10 isoforms reduces malignancy of pinnal-tumor derived cell lines PTD7 and PTD25. **A** Immunofluorescent detection of ectopic expression of Pcdh10 (long isoform 4) at cell-cell contacts (arrows) in transduced and FACS sorted PTD7_RL cultures, using MAb 5G10 as primary antibody specific for long Pcdh10 isoforms (Additional file 8: Table S7). Scale bar: 20 μm. **B** Immunofluorescent detection of ectopic expression of HA-tagged Pcdh10_short isoform 1 at cell-cell contacts (arrows) in transduced and FACS sorted PTD25_RS cultures, using anti-HA as primary antibody. Scale bar: 20 μm. **C,D** Allograft formation after s.c. injection of single-cell suspensions. Tumor volumes are the average + SD for 4 to 5 injected mice. See Additional files 18 and 22: Tables S12 and S14 for quantitation and statistical analysis. **C** Inoculation of PTD7 cells and their Pcdh10 rescued derivatives. PTD7_RS expresses ectopic Pcdh10_short isoform 1; PTD7_RL expresses the long isoform 4. The number of inoculated cells was 10^6^ and 10^5^ as indicated. **D** Inoculation of PTD25 cells and their Pcdh10 rescued derivatives. PTD25_RS expresses ectopic Pcdh10_short isoform 1; PTD25_RL expresses the long isoform 4. The number of inoculated cells was 10^5^ and 10^4^ as indicated

PTD7 and PTD25 cell lines were transduced with lentiviruses expressing either the short isoform 1 of mouse Pcdh10, or the long isoform 4 of mouse Pcdh10, both C-terminally HA-tagged. Transduced cells were selected by blasticidin and further enriched by FACS using an antibody recognizing the extracellularly exposed amino-terminal end of Pcdh10. The selected cell pools _RS (rescued with short Pcdh10 isoform) and _RL (rescued with long isoform) showed fair expression of HA-tagged Pcdh10 isoform-1 or − 4 at cell-cell contacts (Fig. 12A,B). The Pcdh10 rescue converted the refractile PTD25 cells to flat and adherent cells, which had lost desmin expression (Fig. 9; Additional file 21: Fig. S11). In addition, in all Pcdh10 rescued derivatives, anchorage-independent growth and in vitro invasion into collagen matrices were strikingly suppressed or even lost (Table 1; Fig. 10B; Additional file 18: Table S12). Most importantly, allograft formation by the Pcdh10 rescued derivatives was obviously retarded or even abolished (Fig. 12C,D; Table 1; Additional files 18 and 22: Tables S12 and S14).

**Fig. 13:**
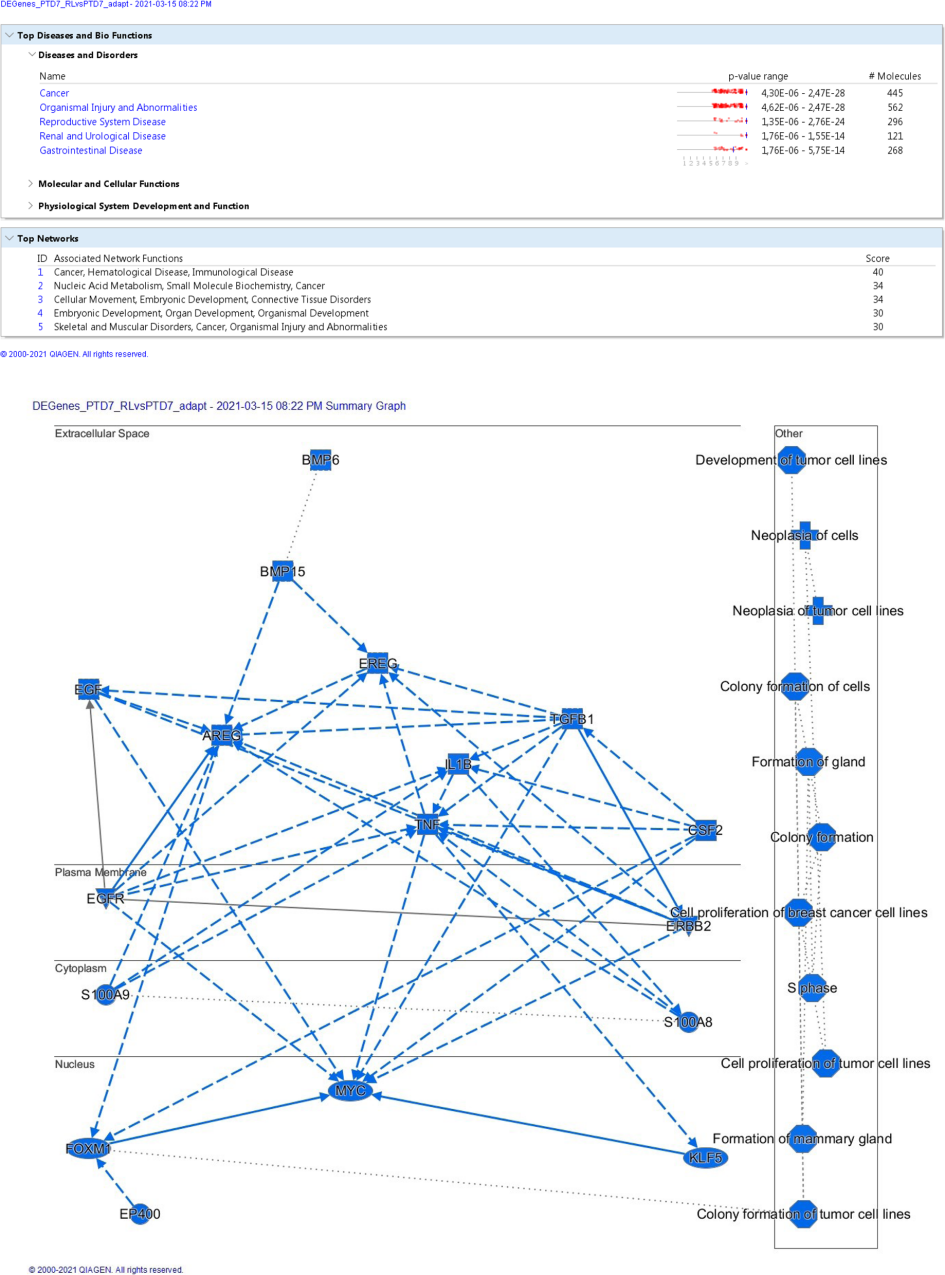
Summary of differentially expressed (DE) genes in Pcdh10-rescued PTD7_RL cells versus malignant Pcdh10-lacking PTD7 cells. The analysis was performed by the Ingenuity Pathway Analysis software (IPA, Qiagen) (see also Table 2). Details on this analysis can be found in https://qiagen.secure.force.com/KnowledgeBase/KnowledgeIPAPage and https://digitalinsights.qiagen.com/products-overview/discovery-insights-portfolio/analysis-and-visualization/qiagen-ipa/. The top network score is based on a *p*-value calculation, which calculates the likelihood that the Network Eligible Molecules that are part of a network are found therein by random chance alone. The graphical summary below provides a coherent and comprehensible synopsis of the major biological themes in the IPA core analysis performed, including upstream regulators, diseases, functions, and pathways, and illustrates how those concepts relate to one another. The algorithm takes into account the magnitude of the differential expression of the corresponding gene in the dataset when deciding which regulators to include. In the left part, major DE genes are indicated in their subcellular compartment, with connecting lines indicating direct (solid lines), indirect (broken lines) or inferred (dotted lines) relationships. In the right part, molecular and cellular functions (octagons) influenced by Pcdh10 re-expression, as well as associated diseases (crosses) are listed. In an IPA core analysis, the z-score of a regulating molecule predicts the activation state of this upstream regulator, using the molecular expression patterns of the molecules downstream of an upstream regulator, being either increased for an activating gene or decreased for an inhibiting gene. Items with a positive z-score (activated) are colored orange (none in this summary), and those with negative z-score (inhibited) are colored blue. Network shapes, used here or in Additional file 24: Figs. S12-S17, include rectangles for cytokines, inverted triangles for kinases, horizontal ovals for transcriptional regulators, vertical rectangles for G-protein coupled receptors, and diamonds for non-kinase enzymes

Given the exclusive nature of this collection of cell lines, we performed an RNA-Seq experiment (Additional files 9-11: Tables S8-S10). First, this analysis confirmed the ablated nature of Pcdh10, p53 and Rb in the PTD7 and PTD25 cell lines, and the Pcdh10 rescue in _RS and _RL derivatives (Additional file 23: Table S15). Genes differentially expressed (DE) between PTD7 or PTD25 and their respective _RL and _RS derivatives were studied by the Ingenuity Pathway Analysis (IPA, Qiagen) (Table 2; Fig. 13; Additional file 24: Figs. S12-S16). In all rescued derivatives, Pcdh10 was the most upregulated molecule. Contrary to what the lists of top-10 upregulated or downregulated DE genes in Table 2 might suggest, there is a considerable agreement between the upregulated or downregulated DE genes in PTD7_RS and PTD7_RL cells (versus PTD7) as well as in PTD25_RS and PTD25_RL cells (versus PDT25) (considering either the highest log2FoldChange or the lowest log2FoldChange values in Additional files 9 and 10: Tables S8 and S9; sheets with both ‘Shared’ and ‘(Sort DE)’ in their names). For instance, 69 out of 75 downregulated genes in PTD7_RS with log2FoldChange values lower than − 2 were shared with those in PTD7_RL, and 156 of 160 upregulated genes in PTD25_RS with log2FoldChange values higher than + 4 were shared with those in PTD25_RL. It was striking that in all comparisons about 50% of the DE genes was associated at high significance with cancer-related pathways (Table 2). However, also DE genes in parental PTD25 compared to parental PTD7 showed such high association with cancer, likely reflecting the higher tumorigenicity of PTD25 (Table 1; Fig. 11; Additional files 19 and 24f: Figs. S9 and S17). The occurrence of the keyword ‘cancer’ in the associated top gene networks and the occurrence of cancer-related terms in the graphical summaries of the IPA analyses (Table 2; Fig. 13; Additional file 24: Figs. S12-S16) added significance to the DE genes observed in these rescued derivatives with lower malignancy. About half of the DE genes of PTD7_RS were shared by the PTD7_RL cells, whereas more than 80% of DE genes of PTD25_RL were shared by PTD25_RS cells (Table 2). These shared DE genes were almost always differential in the same direction (up- or downregulated) (Additional files 9 and 10: Tables S8, S9). DE genes in PTD7_RL were more often associated with cell death networks than DE genes in PTD7_RS (Table 2). However, there were almost no DE genes shared between any rescued PTD7 population and any rescued PTD25 cell population, reflecting also the large difference between gene activity in, respectively, parental PTD7 and PTD25 (Table 2, Additional files 23 and 24f: Table S15 and Fig. S17). The myogenic differentiation in PTD25 is striking, as well as its inhibition in both rescued derivatives PTD25_RS and PTD25_RL.

**Table 2:** IPA^a^ analyses of DE genes in RNA-Seq data for PTD7, PTD25 and their Pcdh10-rescued derivatives

Analysis type^b^	DE genes		DE genes		DE genes		DE genes		DE genes	
Cell line #1 vs.	PTD7_RS vs.		PTD7_RL vs.		PTD25_RS vs.		PTD25_RL vs.		PTD25 vs.	
Cell line #2	PTD7		PTD7		PTD25		PTD25		PTD7	
# Analysis-ready molecules	399		909		1378		1228		1275	
# up	113		430		719		659		887	
# down	286		479		659		569		388	
Top associated disease #1	Cancer		Cancer		Cancer		Cancer		Cancer	
p-value range	2,8E-04- > 1.1E-18		4.3E-06- > 2.5E-28		1.0E-09- > 1.2E-51		9.6E-11- > 5.3E-53		5.3E-09- > 3.9E-40	
# molecules involved	209 (52%)		445 (49%)		705 (51%)		648 (53%)		650 (51%)	
Keyword in top networks^c^	Cancer	Cell death	Cancer	Cell death	Cancer	Cell death	Cancer	Cell death	Cancer	Cell death
# Hits in # top networks	7 of 20	2 of 20	5 of 25	5 of 25	3 of 25	1 of 25	3 of 25	2 of 25	1 of 25	2 of 25
Score of relevant networks	41- > 15	31;23	40- > 23	30- > 15	32- > 26	23	33- > 21	26;24	25	25;22
Range of molecules involved	28- > 14	23;19	32- > 23	27- > 18	31- > 28	26	31- > 24	27;26	27	27;25
Top 10 upregulated DE genes /	**Pcdh10**	**5.73**	**Pcdh10**	**6.10**	**Pcdh10**	**8.92**	**Pcdh10**	**10.14**	**H19**	**15.16**
log2FoldChange	Ces1g	4.06	Cbr2	3.68	Plin4	7.10	Ccl9	7.19	Des	13.52
	Edn1	3.34	Ces1g	3.66	Megf6	6.83	Plin4	7.12	Myog	13.50
	Cbr2	2.38	Ifitm1	3.66	Ndnf	6.75	Slc1a3	7.01	Igfbp5	13.45
	Ctsw	2.32	Slc40a1	3.13	Cdhr1	6.71	C1s	6.92	Actc1	13.00
	Dynap	2.13	Klhl30	3.04	Chst1	6.65	Serpina3	6.82	Cdh15	12.54
	Klhl30	2.01	Prelp	3.03	Col8a2	6.58	Megf6	6.77	Myod1	12.45
	Tnnt2	1.97	Chst1	2.97	Postn	6.56	Vdr	6.62	Igf2	12.18
	Il33	1.94	Aldh3a1	2.96	C1s	6.34	Pvalb	6.61	Myl1	12.14
	Il7	1.92	Kif21b	2.91	Sp8	6.34	Ppl	6.46	Myh3	11.72
Top 10 downregulated DE genes /	Cyba	−4.97	Cyba	−5.05	Pnma8b	−10.45	Krt8	−10.36	Crabp1	-11.64
log2FoldChange	Ptgis	−4.87	Armcx2	−4.91	Wnt7b	−10.40	Thbs4	−9.73	Il1rl1	-10.76
	Armcx2	−4.49	Tcf24	−4.83	Thbs4	−10.37	Nefl	−9.48	Rpl39l	-10.25
	Armcx5	−4.46	Ptgis	−4.81	Bex1	−10.29	Col13a1	−9.43	Xist	-10.03
	Sdk1	−4.31	Tuba8	−4.79	Gal	−10.21	Naip1	−9.17	Dlk1	-9.59
	Nid1	−4.27	Sprr1a	−4.67	Nefm	−9.98	Heyl	−9.14	Slit3	-9.52
	Angptl4	−4.11	Angptl4	−4.50	Crhr1	−9.96	Nefm	−9.12	Morc1	-9.00
	Bmp2	−4.06	Dapk1	−4.48	Neffl	−9.84	Grik3	−9.04	Prxl2ad	-8.96
	Tcf24	−3.93	Armcx3	−4.47	Peg3	−9.71	Wnt7b	−9.02	Barx1	-8.95
	Lonrf3	−3.62	Bmp2	−4.46	Actn2	−9.69	Tmem30b	−8.48	Irx1	-8.54
**Analysis type**^d^	**Shared DE genes**				**Shared DE genes**					
Cell lines #1 vs.	(PTD7_RS and _RL) vs.		% of DE genes of		(PTD25_RS and _RL) vs.		% of DE genes of			
Cell line #2	PTD7		PTD7_RS	PTD7_RL	PTD25		PTD25_RS	PTD25_RL		
# Analysis-ready molecules	233		58	26	1014		74	83		
# up	63		56	15	520		72	79		
# down	170		60	35	494		75	87		
Keyword in top networks^c^	Cancer	Cell death			Cancer	Cell death				
# Hits in # top networks	3 of 14	3 of 14			2 of 25	1 of 25				
Score of relevant networks	36- > 20	22- > 14			36;36	24				
Range of molecules involved	23- > 15	16- > 12			31;31	25				
**Analysis type**^e^	**Unique DE genes**		**Unique DE genes**		**Unique DE genes**		**Unique DE genes**			
Cell line #1 vs.	PTD7_RS (NOT _RL) vs.		PTD7_RL (NOT _RS) vs.		PTD25_RS (NOT_RL) vs.		PTD25_RL (NOT _RS) vs.			
Cell line #2	PTD7		PTD7		PTD25		PTD25			
# Analysis-ready molecules	164		675		361		207			
# up	50		366		198		132			
# down	114		309		163		75			
Keyword in top networks^c^	Cancer	Cell death	Cancer	Cell death	Cancer	Cell death	Cancer	Cell death		
# Hits in # top networks	4 of 8	0 of 8	5 of 25	5 of 20	3 of 19	1 of 19	2 of 11	1 of 11		
Score of relevant networks	51- > 20	n.a.	28- > 13	47- > 21	36- > 15	14	23;19	13		
Range of molecules involved	27- > 14	n.a.	24- > 15	33- > 20	25- > 14	13	16;14	11		

^a^The analysis was performed by the Ingenuity Pathway Analysis software (IPA, Qiagen). See also legend to Fig. 13.

^b^Differentially expressed (DE) genes were determined as described in Materials and Methods. See Additional files 9-11: Tables S8-S10 for detailed data

^c^We consider top networks as those with a score higher than 10 (i.e. there is less than a 1 in 10E10 chance that the Network Eligible Molecules found in that network appeared there just by chance)

^d^DE genes detected in both _RS and _RL derivatives compared to their respective parental cell line. See Additional files 9 and 10: Tables S8 and S9 for detailed data

^e^DE genes detected in one rescued derivative (either _RS or RL) and not in the other derivative as compared to their respective parental cell line. See Additional files 9 and 10: Tables S8 and S9 for detailed data

## Discussion

In former studies, the inactivation of *PCDH10* has been demonstrated in many different human tumor types, and was often found to be important for tumor progression and poor survival. In addition to numerous studies on the cell biological consequences of PCDH10 re-expression or silencing in tumor cell lines, this is the first report of a proven tumor suppressor effect of Pcdh10 in an in vivo model. Moreover, we have compared ablation of all isoforms versus ablation of only long isoforms of mouse Pcdh10. As a full-knockout of *Pcdh10* was reported to be early lethal [49], we opted for the generation of floxed alleles of Pcdh10, so that any variation of spatiotemporal ablation should be possible. Surprisingly, when we used a total-deleter Cre [56] to ablate *Pcdh10* in all tissues, we were unable to observe either early lethality or growth retardation. Nonetheless, our analyses at genomic, RNA and protein levels confirmed this total ablation. One difference with the reported *Pcdh10*-null mouse is the extent of the deletion. Due to the location of the 5’loxP site in our targeting construct for conditional deletion of all isoforms of *Pcdh10*, we ablated also exon #1 of the lncRNA (long noncoding RNA) gene 2610316D01Rik, as this is overlapping with the upstream region of the *Pcdh10* gene. We are not aware of any role of 2610316D01Rik in either mouse development and survival, or tumorigenicity. Moreover, our finding that in a GFAP-Cre^tg/+^;p53^fl/fl^;Rb^fl/fl^ mouse model, the conditional ablation of all Pcdh10 isoforms (including the first exon of 2610316D01Rik) resulted in similar enhanced tumorigenicity, i.e. increased formation of medulloblastoma and pinnal tumors, as the ablation of only long *Pcdh10* isoforms indicates strongly that the involvement of 2610316D01Rik was minor or even nihil. Alternatively, differences between the phenotypes of the present *Pcdh10* KO mouse and the previously reported KO mouse may be ascribed to the presence of an active neo^r^ gene in the genome of the latter KO mouse [49]. This possibility is currently under investigation by one of the authors (S.H.). Interestingly, our mice with ubiquitous deletion of either all isoforms of Pch10 or specifically the long isoforms of Pcdh10, did not develop detectable tumors or other obvious pathologies during an observation period of up to 16 months.

In our hands, the generation of medulloblastoma in the GFAP-Cre^tg/+^;p53^fl/fl^;Rb^fl/fl^ model was strictly dependent on conditional ablation of both *Trp53* and *Rb1* tumor suppressor genes, fully in line with the original report of this model [54]. The aggravating effect of *Pcdh10* ablation in this model confirms a tumor promoting role for *Pcdh10* inactivation, fully in line with the frequent observations of *PCDH10* silencing in human cancers, including medulloblastoma [11]. The surprising finding of pinnal tumors in this genetic mouse model, provided the ear is inflamed by monel number tagging, adds relevance to this mouse model. Indeed, inflammation is a well-known tumor promoting condition [68]. The complicating factor that both medulloblastoma and pinnal tumors arise in the same model, can be addressed by maintaining *Rb1* wild-type. Under those conditions, medulloblastoma do not appear, while generation of pinnal tumors is dependent on floxed alleles of both *Trp53* and *Pcdh10*, in combination with GFAP-Cre. Moreover, these pinnal tumors arise with long latency period, alike the human situation, and are readily accessible for measurements, biopsy, explantation, amputation, and local therapies such as irradiation and thermal therapy.

Self-renewing and multipotent postnatal neural stem cells have been proposed to be the progenitor cells of the medulloblastomas appearing in the GFAP-Cre^tg/+^;p53^fl/fl^;Rb^fl/fl^ model [64]. The following lines of evidence strengthen our opinion that also the pinnal tumors in our model arise from postnatal somatic stem cells, i.e. the cartilage stem cells of the mouse auricular perichondrium [65]. Only those cells in the pinna showed detectable GFAP-positivity and should express GFAP-Cre at that location. They are CD44-positive [65], and indeed the pinnal tumors generally expressed this marker. Also, the pinnal tumors followed diverse lines of partial differentiation. Moreover, the tumor cell populations contained self-renewing cells, which could be readily explanted and showed intrinsic tumorigenicity in allografting experiments, even upon extensive dilution. Upon inoculation of allograft-derived cultures, cells with high tumorigenicity were clearly enriched. These criteria are in accordance with those of cancer stem cells [67, 68].

We compared the PTD7 and PTD25 cell lines, before and after rescuing with either the short or the long isoform of mouse Pcdh10, in several assays, and performed also RNA-Seq experiments. Anchorage-independent growth, invasion into collagen matrices and s.c. allograft formation were inhibited in both PTD cell lines by re-expressing either isoform of Pcdh10. It is the first time that these two types of Pcdh10 isoforms have been functionally compared. Because the short isoform of Pcdh10 lacks several conserved sequences (CM1, CM2, WIRS) [2, 5, 6] it indicates that these regions are not essential for tumor suppression. On the other hand, the short isoform still comprises the iconic ectodomain of this protein family, involved in homophilic trans interactions [4], and also a cytoplasmic domain containing the proteasome interacting region (PIR), which is evolutionarily highly conserved among Pcdh10 proteins [3, 53]. In view of the size of this PIR (100 AA), it is likely that even more, still to be identified, molecular interactions occur through this membrane-proximal cytoplasmic domain.

Our RNA-Seq experiment, summarized in Table 2, allowed us to scrutinize the possible signalling functions of Pcdh10 and the poorly studied similarities or dissimilarities between short and long isoforms of Pcdh10. Although the consequences of Pcdh10 re-expression in biological assays were very similar in rescued PTD7 and PTD25 cell lines, it was surprising how different the RNA-Seq results were between these two families of cell lines. The number and identities of differentially expressed (DE) genes were quite similar for PTD25_RS and -RL cell lines, rescued with, respectively the short and the long isoform of Pcdh10 (compare for instance the IPA analyses in Additional files 24b, 24c and 24e: Figures S13, S14 and S16). This was less the case for PTD7_RS and -RL cell lines although they still shared approximately 50% of their DE genes (Fig. 13, Additional files 24a and 24d: Figures S12 and S15). Remarkably, the PTD7 cell lines re-expressing either long or short Pcdh10 isoforms hardly shared DE genes with rescued PTD25 cell lines. This situation might be related to the extent of Pcdh10 re-expression (increasing from a log2 Fold Change value of 5.73 to 10.14; see Table 2), but more likely it reflects the different molecular nature of the parental PTD cell lines (column at the far right in Table 2; Additional file 24f: Fig. S17). The differentiation towards a muscle-like gene expression pattern of PTD25 cells is obvious, as well as its prominent inhibition in both PTD25_RS and PTD25_RL derivatives (Additional files 10, 23, 24e and 24f: Tables S9 and S15, Figs. S16 and S17). Despite these disparate differential gene expression patterns in the parental cell lines, we believe that our RNA-Seq data are reliable and informative. From this experiment, we can conclude that Pcdh10 re-expression triggers completely different transcriptional changes in PTD7 versus PTD25, and that very few genes showed Pcdh10 isoform-specific changes seen in both PTD7_RL and PTD25_RL cells. Thus, we conclude that the short isoform of Pcdh10 is the major driver in the suppression of malignancy, observed by us in the PTD7 and PTD25 cell lines.

**Fig. 14:**
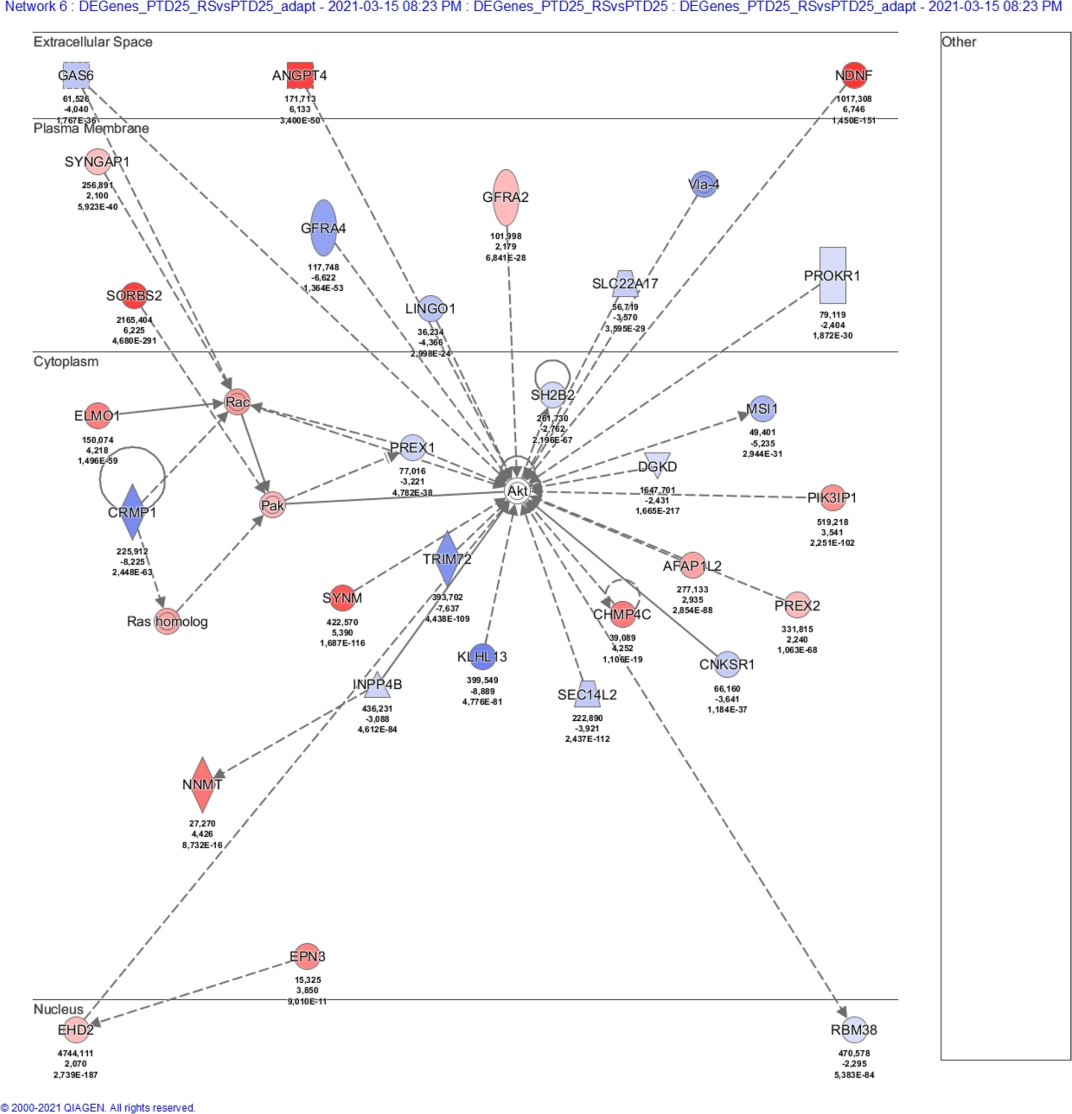
IPA-generated ‘Cell signalling’ network of DE genes in Pcdh10-rescued PTD25_RS versus Pcdh10-lacking PTD25 cells. The analysis was performed by the Ingenuity Pathway Analysis software (IPA, Qiagen) (see also Table 2 and legends to Fig. 13 and Additional file 24b: Fig. S13). The network graph depicts how a selection of DE genes relates to one another and to some key molecules. The genes are shown in their respective subcellular compartment, with connecting lines indicating direct (solid lines) or indirect (broken lines) relationships. DE genes with a positive z-score (activated) are colored orange, and those with a negative z-score (inhibited) are colored blue. Underneath each of the DE genes is given: baseMean value (mean of normalized counts for all PTD25 and PTD25_RS samples), log2FoldChange (condition PTD25_RS versus PTD25), p_adj_ (Benjamin-Hochberg adjusted Wald test p-value). Network shapes used here include rectangles with broken lines for growth factors, trapezia for transporters, vertical ovals for transmembrane receptors, vertical rectangles for G-protein coupled receptors, inverted triangles for kinases, triangles for phosphatases, vertical diamonds for non-kinase enzymes, circles for other molecules

**Fig. 15:**
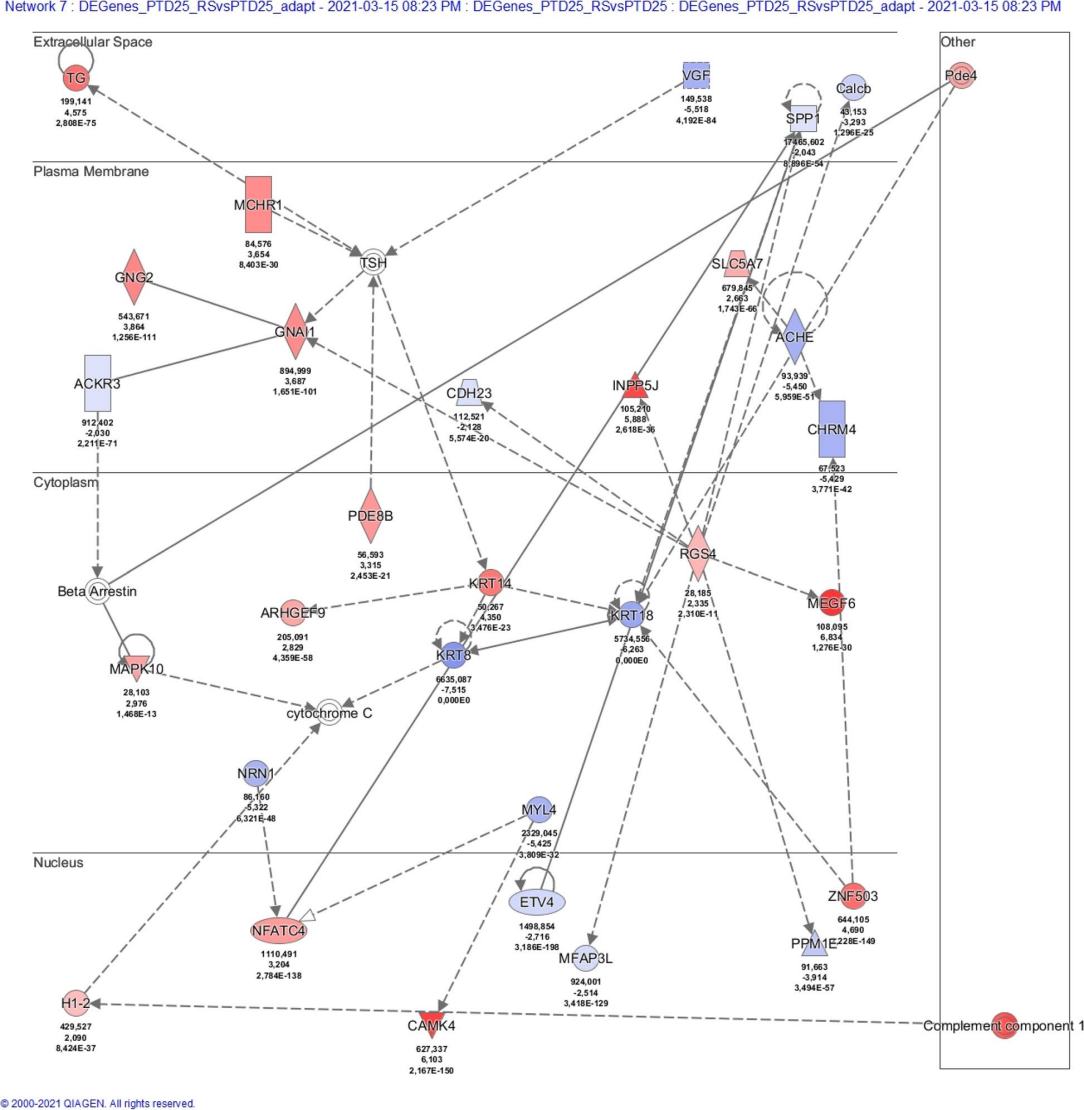
IPA-generated ‘Cell behavior’ network of DE genes in Pcdh10-rescued PTD25_RS versus Pcdh10-lacking PTD25 cells. The analysis was performed by the Ingenuity Pathway Analysis software (IPA, Qiagen) (see also Table 2 and legends to Fig. 13 and Additional file 24b: Fig. S13). The network graph depicts how a selection of DE genes relates to one another and to some key molecules. See legend to Fig. 14 for more details. Network shapes used here include rectangles with solid rim for cytokines, rectangles with broken lines for growth factors, trapezia for transporters, vertical rectangles for G-protein coupled receptors, inverted triangles for kinases, triangles for phosphatases, vertical diamonds for non-kinase enzymes, horizontal ovals for transcriptional regulators, circles for other molecules, double-rimmed circles for complexes or groups

We further addressed the general tendencies of the DE gene collections by IPA analyses (Table 2) and by detailed checking a selection of 134 key genes (Additional file 23: Table S15). Although an overrepresentation of cancer-relatedness is known for IPA analyses - and reflects the multitude of ‘normal’ genes possibly influenced by cancer - it is nonetheless striking how much of the identified DE genes are recognized to be significantly involved in molecular networks related to cancer or cell death (Table 2). We then compared our findings on the 134 selected genes with the scientific literature on PCDH10 interaction partners and associated signalling pathways. An interaction of PCDH10 with hBex1 was reported to be important for imatinib-induced apoptosis of Bcr/Abl + leukemic cells [28]. The gene *Bex1* was strongly suppressed in PTD25_RS and PTD25_RL cells but not influenced in the PTD7 cell family. An interaction with hTERT decreased telomerase activity in pancreatic cancer cells [39]. *Tert* transcripts were not expressed in our PTD cell families. A negative influence of PCDH10 on β-catenin and canonical Wnt signalling has been observed in several systems. In endometroid endometrial cancer cells with re-expressed PCDH10, transcription of the Wnt target gene *MALAT1* was suppressed [35]. In lymphomas, an inverse relationship between protein levels of PCDH10 and β-catenin was seen, but mRNA levels of β-catenin were not influenced by PCDH10 re-expression [44]. In multiple myeloma cells, re-expression of PCDH10 suppressed nuclear localization of β-catenin, activity of LEF/TCF, expression of the β-catenin transcriptional cofactor BCL9, and of AKT, whereas expression of GSK3β was increased [37]. We found no clear influences of Pcdh10 re-expression on transcripts related to the β-catenin/Wnt signalling pathway. Induction of apoptosis by re-expression of PCDH10 has been ascribed to inhibition of NFκB signalling in multiple myeloma [36], and to inhibition of the PI3K/AKT pathway in hepatocellular carcinoma cells [43]. An interaction between PCDH10 and PI3K p85 was detectable [43]. More recently, interaction of PCDH10 with EGFR resulted in decreased AKT signalling, increased p53-mediated apoptosis, decreased β-catenin signalling, decreased epithelial-mesenchymal transition and decreased cancer cell stemness [45]. Our findings on apoptosis-related genes (see for instance Additional file 25: Fig. S18), are not very consistent with either induction or repression of apoptosis in the Pcdh10 rescued cell lines, although transcriptional repression of pro-apoptotic *Bcl6b* is seen in all four rescued cell lines (Additional file 23: Table S15). Transcriptional changes, as analyzed by the RNA-Seq approach, form of course only one level of molecular regulation, and translational and posttranslational changes, which are of major importance in processes like Wnt signalling, NFκB signalling and apoptosis regulation are not directly measured in this way. Nonetheless, several RNA-Seq observations are worthwhile investigating further. For instance, *Pik3ip1*, encoding phosphoinositide-3-kinase interacting protein 1, is transcriptionally induced in all four Pcdh10 rescued cell lines (Additional file 23: Table S15). Pik3ip1 is a negative regulator of Pi3k and a tumor suppressor in a mouse model of hepatocellular carcinoma [69]. Pi3k is an upstream activator of Akt, and indeed, numerous Akt stimulating events can be found in the DE genes of Pcdh10 rescued cell lines (exemplified in Fig. 14). Among the most drastic downregulated molecules in Pcdh10 rescued PTD25 cell lines, but not in rescued PTD7 cell lines, are the transcripts of *Krt8* and *Krtr18* (exemplified in Fig. 15). The encoded keratin proteins K8 and K18 form heteropolymers, which sequester TRADD and attenuate tumor necrosis factor-induced cell death [70]. Such findings underline that different signalling pathways can be followed to suppress malignancy in PTD7 and PTD25 cell families by Pcdh10 re-expression.

## Conclusions

In summary, we present here exclusive data strongly supporting the concept of PCDH10 being an important tumor suppressor in human malignancies. We have shown this by generating innovative floxed *Pcdh10* alleles in mice. Surprisingly, the complete ablation of either all isoforms of Pcdh10 or only long isoforms of Pcdh10 did not generate mortality or growth retardation. Spontaneous tumors were not observed. On the other hand, the breeding of these mice bearing floxed *Pcdh10* alleles with suitable Cre-expressing mice and with available mouse cancer models will be a most valuable addition to the research on PCDH10 inhibition in cancer. We demonstrated this successfully in a medulloblastoma mouse model, and by serendipity, found that also inflammation-dependent pinnal tumors arose. The latter phenomenon forms an interesting model to pursue, for instance with respect to cancer cell stemness. The malignancy of the cell lines derived from such pinnal tumors was efficiently suppressed by both short and long isoforms of re-expressed Pcdh10. From this and RNA-Seq data of these cell families, we conclude that, at least in this cancer model, the short isoform of Pcdh10 is a major effector of tumor suppressor activity, what has not been reported before. Moreover, Pcdh10-mediated tumor suppression can apparently follow different and complex signalling pathways in various tumor cell systems. The further use of our mice with floxed *Pcdh10* alleles might become a most rewarding tool in contemporary cancer research.

## Supplementary Information

**Additional file 1. **Strategy for conditional knockout of all isoforms of the *Pcdh10* allele. Includes textual description of the strategy, Fig. S1 (Schematic representation of the recombineering strategy of the Pcdh10all targeting construct) and Table S1 (Recombineering primers used for generation of the Pcdh10all targeting construct).

**Additional file 2. **Strategy for conditional knockout of the long isoforms of the *Pcdh10* allele. Includes textual description of the strategy, Fig. S2 (Schematic representation of the recombineering strategy of the Pcdh10long targeting construct) and Table S2 (Recombineering primers used for generation of the Pcdh10long targeting construct).

**Additional file 3: Table S3**. Primers used for generation of Southern probes.

**Additional file 4: Fig. S3**. Verification of targeted ES cells and recombinant Pcdh10all^fl/fl^ and Pcdh10long^fl/fl^ mice at the genomic level.

**Additional file 5: Table S4**. Genotyping primers used.

**Additional file 6: Table S5**. Primers used for quantitative RT-PCR.

**Additional file 7: Table S6**. Primary antibodies used.

**Additional file 8: Table S7**. Protocadherin-10 specific antibodies used.

**Additional file 9: Table S8**. Differentially expressed (DE) genes in the PTD7 cell family, comprising: *sheet* PTD7_RSvsPTD7 (Comparison of genes, differentially expressed by PTD7_RS versus PTD7), *sheet*. PTD7_RLvsPTD7 (Comparison of genes, differentially expressed by PTD7_RL versus PTD7), *sheet*. Shared_PTD7_RS_AND_PTD7_RL_alph (Comparison of genes, differentially expressed by either PTD7_RS versus PTD7, or PTD7_RL versus PTD7; sorted alphabetically [ID]), *sheet* Shared_PTD7_RS(sortDE)_RL (Comparison of genes, differentially expressed by both PTD7_RS versus PTD7, and PTD7_RL versus PTD7; sorted according to value of log2FoldChange in PTD7_RS vs PTD7); *sheet* Shared_PTD7_RS_RL(sortDE) (Comparison of genes, differentially expressed by both PTD7_RS versus PTD7, and PTD7_RL versus PTD7; sorted according to value of log2FoldChange in PTD7_RL vs PTD7); *sheet* Unique_PTD7_RS_NOT_RL vs PTD7 (Genes, differentially expressed by PTD7_RS versus PTD7, and not shared by genes differentially expressed by PTD7_RL versus PTD7), and *sheet* Unique_PTD7_RL_NOT_RS vs PTD7 (Genes, differentially expressed by PTD7_RL versus PTD7, and not shared by genes differentially expressed by PTD7_RS versus PTD7).

**Additional file 10: Table S9**. Differentially expressed (DE) genes in the PTD25 cell family, comprising: *sheet* PTD25_RSvsPTD25 (Comparison of genes, differentially expressed by PTD25_RS versus PTD25), *sheet* PTD25_RLvsPTD25 (Comparison of genes, differentially expressed by PTD25_RL versus PTD25), *sheet* Shared_PTD25_RS_AND_PTD25_RL (Comparison of genes, differentially expressed by either PTD25_RS versus PTD25, or PTD25_RL versus PTD25; sorted alphabetically [ID]), *sheet* Shared_PTD25_RS(sortDE)_RL (Comparison of genes, differentially expressed by both PTD25_RS versus PTD25, and PTD25_RL versus PTD25; sorted according to value of log2FoldChange in PTD25_RS vs PTD25); *sheet* Shared_PTD25_RS_RL(sortDE) (Comparison of genes, differentially expressed by both PTD25_RS versus PTD25, and PTD25_RL versus PTD25; sorted according to value of log2FoldChange in PTD25_RL vs PTD25); *sheet* Unique_PTD25_RS_NOT_RL vs PTD25 (Genes, differentially expressed by PTD25_RS versus PTD25, and not shared by genes differentially expressed by PTD25_RL versus PTD25), and *sheet* Unique_PTD25_RL_NOT_RS vs PTD25 (Genes, differentially expressed by PTD25_RL versus PTD25, and not shared by genes differentially expressed by PTD25_RS versus PTD25).

**Additional file 11: Table S10**. Differentially expressed (DE) genes in Pcdh10-lacking PTD25 cells versus Pcdh10-lacking PTD7 cells.

**Additional file 12: Fig. S4**. mRNA expression levels of δ-Pcdhs in Pcdh10﻿all^−/−^ and Pcdh10long^−/−^ mouse brains.

**Additional file 13: Fig. S5**. Phenotypic and reproduction data for Pcdh10﻿all^−/−^ and Pcdh10long^−/−^ knockout (KO) mice.

**Additional file 14﻿: Fig. S6**. Body weight curves of Pcdh10all^−/−^ and Pcdh10long^−/−^ KO mice.

**Additional file 15: Table S11**. Statistical analysis of Kaplan Meyer tumor-free curves for GFAP-Cre experiment.

**Additional file 16: Fig. S7**. Examples of validation of antibody specifity in immunohistochemical detection of various antigens on mouse WT and tumoral tissues.

**Additional file 17: Fig. S8**. Immunohistochemical detection of E-cadherin and catenins in representative pinnal tumors.

**Additional file 18: Table S12**. Derivation and detailed analysis of pinnal-tumor derived (PTD) cell lines and rescued derivatives.

**Additional file 19: Fig. S9**. Allograft formation after s.c. injection of PTD single-cell suspensions into athymic nude mice.

**Additional file 20.** Lung colonization assay for selected PTD cell lines. Includes Table S13 and Fig. S10.

**Additional file 21: Fig. S11.** Immunofluorescent detection of desmin in PTD cell cultures and derivatives.

**Additional file 22: Table S14**. Statistical analysis of allograft growth curves for PTD7, PTD25 and their Pcdh10-rescued derivative cell lines.

**Additional file 23: Table S15**. Expression of 134 selected genes in PTD7, PTD25 and derivatives

**Additional file 24: **comprising **Figs. S12-S17**. **24a**: **Fig. S12**. Summary of differentially expressed (DE) genes in PTD7_RS (rescued by short isoform 1 of Pcdh10) versus malignant Pcdh10-lacking PTD7 cells. **24b**: **Fig. S13**. Summary of DE genes in PTD25_RS (rescued by short isoform 1 of Pcdh10) versus malignant Pcdh10-lacking PTD25 cells. **24c**: **Fig. S14**. Summary of DE genes in PTD25_RL (rescued by long isoform 4 of Pcdh10) versus malignant Pcdh10-lacking PTD25 cells. **24d: Fig. S15**. Summary of DE genes shared by both PTD7_RS and PTD7_RL in comparison with malignant Pcdh10-lacking PTD7 cells. **24e: Fig. S16**. Summary of DE genes shared by both PTD25_RS and PTD25_RL in comparison with malignant Pcdh10-lacking PTD25 cells. **24f: Fig. S17**. Summary of DE genes in malignant Pcdh10-lacking PTD25 versus malignant Pcdh10-lacking PTD7 cells.

**Additional file 25: Fig. S18**. IPA-generated ‘Cell death and survival’ network of genes differentially expressed in PTD25_RS (rescued by short isoform 1 of Pcdh10) versus malignant Pcdh10-lacking PTD25 cells.

**Additional file 26.** Original uncropped blots corresponding to Fig. 3, panels A-D (Pcdh10 expression levels in Pcdh10all mice by Western blot analysis). Antibodies used are indicated at the left. Exposure time was 1 min.

**Additional file 27.** Original uncropped blots corresponding to Fig. 3, panels A-D (Pcdh10 expression levels in Pcdh10all^−/−^ mice by Western blot analysis). Antibodies used are indicated at the left. Exposure time was 10 min.

**Additional file 28.** Original uncropped blots corresponding to Fig. 3, panels A-D (Pcdh10 expression levels in Pcdh10all^−/−^ mice by Western blot analysis). Antibody used in reprobing the blots was anti-beta-tubulin. Exposure time was 1 s.

**Additional file 29.** Original uncropped blot corresponding to Fig. 4, panels A-D (Pcdh10 expression levels in Pcdh10long^−/−^ mice by Western blot analysis). Antibodies used are indicated at the left. Exposure time was 30 s.

**Additional file 30.** Original uncropped blot corresponding to Fig. 4, panels A-D (Pcdh10 expression levels in Pcdh10long^−/−^ mice by Western blot analysis). Antibodies used are indicated at the left. Exposure time was 1 min.

**Additional file 31.** Original uncropped blot corresponding to Fig. 4, panels A-D (Pcdh10 expression levels in Pcdh10long^−/−^ mice by Western blot analysis). Antibodies used are indicated at the left. Exposure time was 5 min.

**Additional file 32.** Original uncropped blot corresponding to Fig. 4, panels A-D (Pcdh10 expression levels in Pcdh10long^−/−^ mice by Western blot analysis). Antibody used in reprobing the blots was anti-beta-tubulin. Exposure time was 1 s.

**Additional file 33.** Original blots corresponding to Additional Fig. S3 (panel A, left side): Southern blot analysis of ES cells successfully targeted with the Pcdh10all targeting construct.

**Additional file 34.** Original blots corresponding to Additional Fig. S3 (panel A, right side): Southern blot analysis of ES cells successfully targeted with the Pcdh10long targeting construct.

**Additional file 35.** Animal Facility Procedures and Licenses of the VIB-UGent Center for Inflammation Research (IRC), Ghent, Belgium.

## Data Availability

All data generated or analyzed during this study are included in this published article and its additional supplementary information files. The Pcdh10all^fl/fl^ and Pcdh10long^fl/fl^ mice are available from the Department of the corresponding author on reasonable request.

## Abbreviations

AA: Amino acid
BCCM: Belgian Coordinated Collection of Microorganisms
CM: Conserved motif
ES: Embryonal stem
FACS: Fluorescence activated cell sorting
GFAP: Glial fibrillary acidic protein
HPV: Human papillomavirus
IF: Immunofluorescence
IHC: Immunohistochemistry
IPA: Ingenuity Pathway Analysis software (Qiagen)
IRC: Center for Inflammation Research
KO: Knock-out
LacZ: β-galactosidase
lncRNA: Long noncoding RNA
Nap1: Nck-associated protein 1
Pcdh: Protocadherin
PIR: Proteasome interacting region
PTD: Pinnal-tumor derived
PTDx_RS: PTD cell line (x = 7 or 25), rescued for Pcdh10 deficiency by re-expression of short isoform 1 of mouse Pcdh10
PTDx_RL: PTD cell line (x = 7 or 25), rescued for Pcdh10 deficiency by re-expression of long isoform 4 of mouse Pcdh10
RNA-Seq: RNA sequencing
s.c.: Subcutaneously
TMCF: Transgenic mouse core facility
WASP: Wiskott–Aldrich syndrome protein
WAVE: WASP-Family verprolin homologous protein
WRC: WAVE regulatory complex
