# Supplementary material for: Innovative mouse models for the tumor suppressor activity of Protocadherin-10 isoforms
Source: BMC Cancer. 2022 Apr 25;22:451. doi: 10.1186/s12885-022-09381-y (PMC9040349; doi:10.1186/s12885-022-09381-y)
Supplement: Supplementary file 1 — Additional file 1. Strategy for conditional knockout of all isoforms of the Pcdh10 allele. Includes textual description of the strategy, Fig. S1 (Schematic representation of the recombineering strategy of the Pcdh10all targeting construct) and Table S1 (Recombineering primers used for generation of the Pcdh10all targeting construct). [file 12885_2022_9381_MOESM1_ESM.pdf]

Additional file 1 for Kleinberger, Sanders, Staes et al. (2022)

Strategy for conditional knockout of all isoforms of the *Pcdh10* allele

Cloning of the targeting vector was by recombineering on the basis of the Red/ET recombination system (1, 2). PAC clone RP21-402C3 (MRC Geneservice, UK) served as genomic start material. A fragment comprising exons 1, 2 and 3 of the *Pcdh10* gene was subcloned into the Gateway-compatible vector pStart-T2 (see below: Additional Fig. S1 and Table S1). Thereafter, a first loxP site was inserted upstream of exon 1, followed by insertion into intron 1 of a neo-resistance cassette, flanked by FRT sites and followed by the second loxP site. Finally, by Gateway recombination, the negative selection cassette HSV-tk was introduced downstream of the genomic insert. The final targeting vector (Additional Fig. S1g) was linearized with *Ahd*1 before electroporation of ES cells. All vectors used and generated were quality verified by restriction analysis and DNA sequencing.

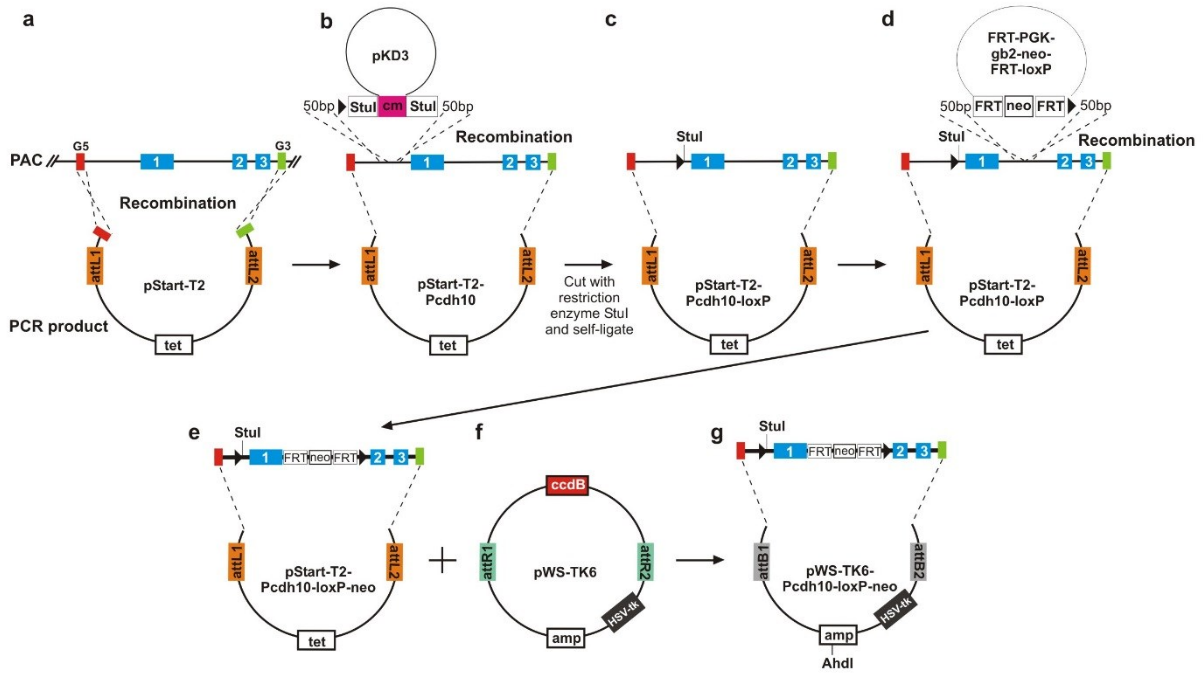

Additional Fig. S1 Schematic representation of the recombineering strategy of the Pcdh10all targeting construct. Numbered blue blocks represent mouse *Pcdh10* exons. All PCR primers used are listed in Additional Table S1. **(a)** To subclone a fragment from PAC clone RP21-402C3 into pStart-T2, two oligonucleotides were designed, which have 50 bp homology to the PAC fragment and 20 bp specific for the pStart-T2 plasmid. These oligos were used as PCR primer. **(b)** To insert the first loxP site, two oligonucleotides were designed for PCR amplification of a chloramphenicol resistance gene (cm) flanked by two unique restriction enzyme sites (*Stu*I) and a loxP site (black triangle). The unique restriction site was designed for use in Southern screening of ES cells, ensuring that the 5' loxP site was inserted. **(c)** The plasmid was cut with the restriction enzyme *Stu*I and self-ligated to remove the cm resistance gene and to obtain a single loxP site and a unique restriction enzyme site upstream of exon 1. **(d)** To insert the second loxP site and a neo-resistance cassette, two oligo-nucleotides were designed for PCR amplification of a neo-resistance cassette flanked by two FRT sites and a loxP site. **(e)** With the resulting plasmid, a Gateway recombination step is performed with the pWS-TK6 plasmid **(f)** to add a negative selection cassette (HSV-tk). **(g)** The final targeting vector contains a unique restriction enzyme site (*Ahd*I) for plasmid linearization.

Additional Table S1 Recombineering primers used for generation of the Pcdh10all targeting construct.

Genomic subcloning	forward	5’CTTTTAGTAAGTGATGTTTTGCCACCCATATTAATGTTTTGTAGTATAAA gccgcactcgagatatctagaccca-3’
	reverse	5’TATCTGAAATTTATTTTGAATTTTGAGGTATGGTCTCACTATGTTGTCCA cgactgaattggttcctttaaagc-3’
First loxP insertion	forward	5’TTTAGTAACCAAGCATTTTGTGGGTAGACATTAGATGGGTAGACGTTCA A**ataacttcgtatagcatacattatacgaagttat*AGGCCT***agcattacacgtcttgagcgattgt-3’
	reverse	5’CAATCAAAGCTAGAGGGTCAGAAGGGGAGGTGTGGGGAGGATAGACC AGG***AGGCCT***cacttaacggctgacatgggaatta-3’
FRT-neo-FRT-loxP insertion	forward	5’CAGACTATGATTAGAGTAATTTTATGGTAGACGCAACAGTTTATTAAAC Cttgtcctactcaggagagcg-3’
	reverse	5’TTGCTGGCCCAGAGAAAATGTGTCTTATTTAAAAGGAGAATGCAAAAA AAacccggtagaattcgtcgac-3’

Uppercase sequences are 50 bases homologous to flanking genomic DNA of *Pcdh10* for Red-recombination. Lowercase sequences are primers homologous to the plasmid DNA to be amplified. Bold italic uppercase sequences correspond to the *Stu*I (AGGCCT) consensus sequence. The bold lowercase sequence is the loxP site.

References

1. Wu S, Ying G, Wu Q, Capecchi MR. A protocol for constructing gene targeting vectors: generating knockout mice for the cadherin family and beyond. Nat Protoc. 2008;3:1056-76.
2. Fu J, Bian X, Hu S, Wang H, Huang F, Seibert PM, et al. Full-length RecE enhances linear-linear homologous recombination and facilitates direct cloning for bioprospecting. Nat Biotechnol. 2012;30:440-6.
