# Supplementary material for: Innovative mouse models for the tumor suppressor activity of Protocadherin-10 isoforms
Source: BMC Cancer. 2022 Apr 25;22:451. doi: 10.1186/s12885-022-09381-y (PMC9040349; doi:10.1186/s12885-022-09381-y)
Supplement: Supplementary file 2 — Additional file 2. Strategy for conditional knockout of the long isoforms of the Pcdh10 allele. Includes textual description of the strategy, Fig. S2 (Schematic representation of the recombineering strategy of the Pcdh10long targeting construct) and Table S2 (Recombineering primers used for generation of the Pcdh10long targeting construct). [file 12885_2022_9381_MOESM2_ESM.pdf]

Additional file 2 for Kleinberger, Sanders, Staes et al. (2022)

Strategy for conditional knockout of the long isoforms of the *Pcdh10* allele

PAC clone RP21-402C3 (MRC Geneservice, UK) served as genomic start material. The targeting vector was constructed by following the Red/ET recombination protocols K003 and K005 from Gene Bridges GmbH (Heidelberg, Germany). Briefly, a fragment of PAC clone RP21-402C3, comprising exons 1, 2 and 3 of the *Pcdh10* gene, was subcloned into the pBluescript II KS+ vector (see below: Additional Fig. S2 and Table S2). Then, a first loxP site was inserted downstream of exon 3, followed by insertion upstream of exon 2 of a neo-resistance cassette, flanked by FRT sites and preceded by a second loxP site. The final targeting vector (Additional Fig. S2e) was linearized with *Sca*1 before electroporation of ES cells. All vectors used and generated were quality verified by restriction analysis and DNA sequencing.

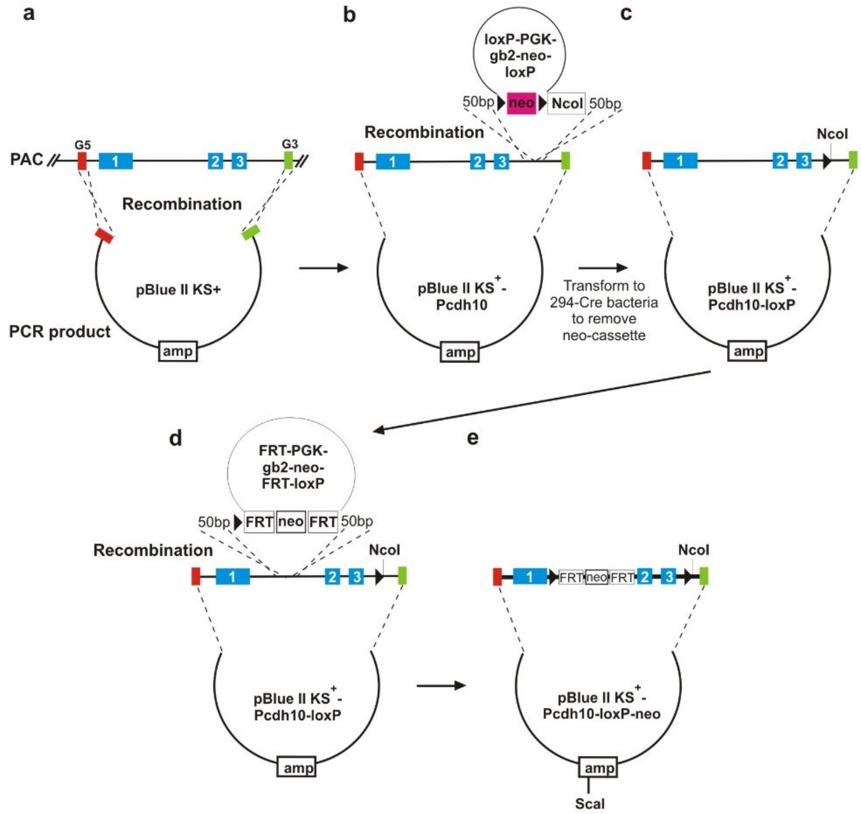

Additional Fig. S2 Schematic representation of the recombineering strategy of the Pcdh10long targeting construct. Numbered blue blocks represent mouse *Pcdh10* exons. All PCR primers used are listed in Additional Table S2. **(a)** To subclone a fragment from a PAC into pBluescript II KS+, two oligonucleotides were designed, which have 50 bases homologous to the PAC fragment while 20 bases are used as PCR primer. **(b)** To insert the first loxP site, two oligonucleotides were designed for PCR amplification of a resistance gene (neo) flanked by two loxP sites and a unique restriction enzyme site (*Nco*I). The unique restriction site is designed for use in Southern screening of ES cells, ensuring that the 3' loxP site is inserted. **(c)** The plasmid was transformed to 294-Cre bacteria to remove the neo resistance gene and to obtain a single loxP site and a unique restriction enzyme site downstream of exon 3. **(d)** To insert the second loxP site and neo-cassette, two oligonucleotides were designed for PCR amplification of a neo resistance cassette flanked by two FRT sites and a loxP site. **(e)** The final targeting vector contains a unique restriction enzyme site (*Sca*I) for plasmid linearization.

Additional Table S2 Recombineering primers used for generation of the the Pcdh10long targeting construct.

Genomic subcloning	forward	5’TGAGATTTCCCTGTAAATAATCAGTGCATCCACTGTAACACTTGAGCCGCat agacggtttttcgccctttgac-3’
	reverse	5’TTACTGATAAAACTGGTGAAATACTCAGGCCTTATTCGAGAGACCTCACTc ccagcttttgttccctttagtga-3’
First loxP insertion	forward	5’GCATACAAAGTATCACTGGAGAAGGGGATGATGTTAAAAGTCACAAATG Gtcccggcggatttgtcctactcaggagagc-3’
	reverse	5’CTATTACTAATGTTATGAAGCCCACAGACACCTGGACTGCAAACTAAAAC ***CCATGG***tctgcaaaccctatgctactccgtc-3’
loxP-FRT-neo-FRT insertion	forward	5’GGAATTGCCTACTGTTGGCTTCTTTCTTCAGCAAAGAATTTGGCTTGTCAcc cctacccggtagaattcgtcgac-3’
	reverse	5’ACCCTCCAACTTTCCACTATGTCTCTACCTTTTAAAAAGAAATTGAAATTcc cggcggatttgtcctactcaggagagc-3’

Uppercase sequences are 50 bases homologous to flanking genomic DNA of *Pcdh10* for Red-recombination. Lowercase sequences are primers homologous to the plasmid DNA to be amplified. Bold italic uppercase sequences correspond to the *Nco*I (CCATGG) consensus sequence.
