# Supplementary material for: Innovative mouse models for the tumor suppressor activity of Protocadherin-10 isoforms
Source: BMC Cancer. 2022 Apr 25;22:451. doi: 10.1186/s12885-022-09381-y (PMC9040349; doi:10.1186/s12885-022-09381-y)
Supplement: Supplementary file 3 — Additional file 3: Table S3. Primers used for generation of Southern probes. [file 12885_2022_9381_MOESM3_ESM.pdf]

Kleinberger, Sanders, Staes et al. (2022)

Additional file 3: Table S3. Primers used for generation of Southern probes

Application	Specificity	Forward (5'->3')	Reverse (5'->3')	Product size
Southern probe generation	5’ probe floxed genome Pcdh10all	TGTATGCAGCCTCCATAGTC	GTGGGAGATGATATGGCACA	392 bp
Southern probe generation	5’ probe floxed genome Pcdh10long	TTTCCATACAGCCCTTGTCT	CACTGAATCCCACCTTCTGC	571 bp
Southern probe generation	3’ probe floxed genomes Pcdh10all and Pcdh10long	GGATGCTGCCTGTATATCAT	CTCTGAGCTCTCAAAGTAGG	486 bp
