# Supplementary material for: Innovative mouse models for the tumor suppressor activity of Protocadherin-10 isoforms
Source: BMC Cancer. 2022 Apr 25;22:451. doi: 10.1186/s12885-022-09381-y (PMC9040349; doi:10.1186/s12885-022-09381-y)
Supplement: Supplementary file 4 — Additional file 4: Fig. S3. Verification of targeted ES cells and recombinant Pcdh10allfl/fl and Pcdh10longfl/fl mice at the genomic level. [file 12885_2022_9381_MOESM4_ESM.pdf]

Additional file 4 for Kleinberger, Sanders, Staes et al. (2022)

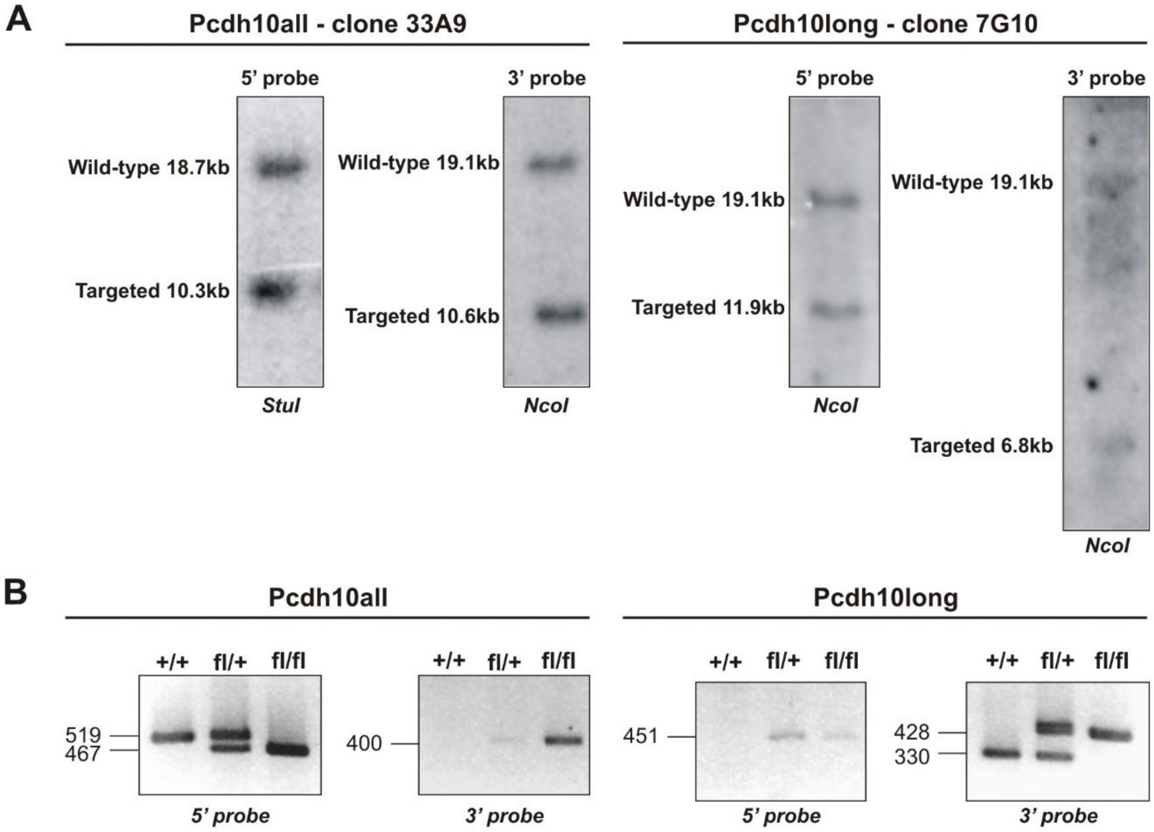

Additional Figure S3. Verification of targeted ES cells and recombinant Pcdh10all$^{fl/fl}$ and Pcdh10long$^{fl/fl}$ mice at the genomic level. See Fig. 1 for genomic location of probes and primers. See Additional files 3 and 5 (Tables S3 and S4) for primer information. **A** Southern blot analysis of ES cells successfully targeted with either the Pcdh10all or Pcdh10long targeting constructs. ES cell clone 33A9 (Pcdh10all) shows an additional band at 10.3 kb for the 5' probe and at 10.6 kb for the 3' probe. ES cell clone 7G10 (Pcdh10long) shows an additional band at 11.9 kb for the 5' probe and at 6.8 kb for the 3' probe. The corresponding original, uncropped Southern blots are shown in Additional files 33 and 34. **B** Genomic PCR verification of the genotype of wild-type (+/+), heterozygous (fl/+) and homozygous (fl/fl) floxed mice. Pcdh10all mice show a 519 bp band for the wild-type and a 467 bp band for the floxed allele at the 5' loxP site, and no band for the wild-type and a 400 bp band for the floxed allele at the 3' loxP site. Pcdh10long mice show no band for the wild-type and a 451 bp band for the floxed allele at the 5' loxP site, and a 330 bp band for the wild-type and a 428 bp band for the floxed allele at the 3' loxP site.
