# Supplementary material for: Innovative mouse models for the tumor suppressor activity of Protocadherin-10 isoforms
Source: BMC Cancer. 2022 Apr 25;22:451. doi: 10.1186/s12885-022-09381-y (PMC9040349; doi:10.1186/s12885-022-09381-y)
Supplement: Supplementary file 5 — Additional file 5: Table S4. Genotyping primers used. [file 12885_2022_9381_MOESM5_ESM.pdf]

Kleinberger, Sanders, Staes et al. (2022)

Additional file 5: Table S4. Genotyping primers used

					PCR product size (bp)		
Specificity	Label*	Forward (5'->3')	Label*	Reverse (5'->3')	WT allele	floxed	deleted
Floxed Pcdh10all allele (5’ end)	A	CATGTACCTTCTTCCCACAC	B	GGCATGTGTCAATCAAAGCT	519	467	n.a.
Floxed Pcdh10all allele (3’ end)	C	CGTATAGCATACATTATACG	D	GGTCTGTCAACTAGATAGCT	/**	400	n.a.
Ablated Pcdh10all allele	A	CATGTACCTTCTTCCCACAC	D	GGTCTGTCAACTAGATAGCT	/***	n.a.	775
Floxed Pcdh10long allele (5’ end)	E	GCGTGTACCAGTAAGCAATA	F	CGTATAGCATACATTATACG	/**	451	n.a.
Floxed Pcdh10long allele (3’ end)	G	CTTCCATTGGTCACTGTGCT	H	CCACCTTTGGCCATTAGTTA	330	428	n.a.
Ablated Pcdh10long allele	E	GCGTGTACCAGTAAGCAATA	H	CCACCTTTGGCCATTAGTTA	/***	n.a.	652
GFAP-Cre trangene	/	GCCTGCATTACCGGTCGATGCAACGA	/	GTGGCAGATGGCGCGGCAACACCATT	800	n.a.	n.a.
Floxed p53	/	CACAAAAACAGGTTAAACCCAG	/	AGCACATAGGAGGCAGAGAC	288	370	n.a.
Ablated p53	/	CACAAAAACAGGTTAAACCCAG	/	GAAGACAGAAAAGGGGAGGG	/***	n.a.	612
Floxed RB1	/	GGCGTGTGCCATCAATG	/	AACTCAAGGGAGACCTG	650	700	/

*: Label on Fig. 1

/**: No product as primer C (= primer F) is located on the LoxP site

/***: Product is too long for routine PCR test
