# Supplementary material for: Innovative mouse models for the tumor suppressor activity of Protocadherin-10 isoforms
Source: BMC Cancer. 2022 Apr 25;22:451. doi: 10.1186/s12885-022-09381-y (PMC9040349; doi:10.1186/s12885-022-09381-y)
Supplement: Supplementary file 6 — Additional file 6: Table S5. Primers used for quantitative RT-PCR. [file 12885_2022_9381_MOESM6_ESM.pdf]

Additional file 6: Table S5 Primers used for quantitative RT-PCR

Mouse gene	Forward (5’->3’)	Reverse (5’->3’)
Pcdh10all	GAGCAGGAACATGGCACTTT	AGTAAGGGGTCCGTGAGTTG
Pcdh10long	ACAGTGGTCATGGAGACAGT	TCCTCGGTGCAGTTGGAGAA
Pcdh10short	CCAAGTCAATTCTGCTCTGG	GTGCCAGAGGAACTTCATTC
Pcdh1	CTGCTCCTATTGGCTCCATCT	CACGTCTGGTAAACCGTAGTC
Pcdh7	AACGATAACACGCCCACCTT	GCCATTGCGACCAAAGTCAC
Pcdh8	CTCAGTGGCCCAGAGCAAGA	CCGGAGCAGAGAGCTGTTGA
Pcdh9	TTTGATCAACAGCCGCTTTC	CTCCGGAGTTTCCACGATAT
Pcdh11	CGGAGAACTCGGCTATAAAC	CAATGACATCAAGTCCGAAA
Pcdh17	TCAGTGCCAGAGGAGCAAGG	AGATGCGGTGCGGAGTTCTC
Pcdh18	AGCATAGGGGCTAAAATTGA	CAGCACGTCCACTTCAATGT
Pcdh19	TTGACCGAGACCTGCTGTGC	TGGGAGCATTGTCGTTCAGG
Gapdh	TGTGTCCGTCGTGGATCTGA	TTGCTGTTGAAGTCGCAGGAG
Hprt	AGTGTTGGATACAGGCCAGAC	CGTGATTCAAATCCCTGAAGT
Rpl13a	CCTGCTGCTCTCAAGGTTGTT	TGGTTGTCACTGCCTGGTACTT
Sdha	CTTGAATGAGGCTGACTGTG	ATCACATAAGCTGGTCCTGT
Ubc	AGCCCAGTGTTACCACCAAG	ACCCAAGAACAAGCACAAGG
