# Supplementary material for: Innovative mouse models for the tumor suppressor activity of Protocadherin-10 isoforms
Source: BMC Cancer. 2022 Apr 25;22:451. doi: 10.1186/s12885-022-09381-y (PMC9040349; doi:10.1186/s12885-022-09381-y)
Supplement: Supplementary file 7 — Additional file 7: Table S6. Primary antibodies used. [file 12885_2022_9381_MOESM7_ESM.pdf]

Kleinberger, Sanders, Staes et al. (2022)

Additional file 7: Table S6. Primary antibodies used

Antigen	Antibody name	Antibody type	Source	Cat. No.	Applications in this report[a]	Working dilution
Beta-catenin	mouse anti-β-catenin	Mouse MAb, clone 14	BD Transduction Laboratories, San Jose, CA, USA	610154	IHC	1/400
CD44	purified Rat anti-Mouse CD44	Rat MAb, clone IM7	BD Pharmingen, San Diego, CA, USA	550538	IHC	1/50
HA-tag	mouse anti-HA-tag	Mouse MAb, clone HA-7	Sigma-Aldrich, Merck, Darmstadt, Germany	H9658	WB; IF	1/1000
Keratin 1	Purified anti-Keratin 1	Rabbit PAb, IgG fraction, Poly19056	BioLegend, San Diego, CA, USA (previously Covance)	905601 (PRB-165P)	IHC	1/1000
Desmin	Rabbit anti-Desmin	Rabbit PAB	Abcam, Cambridge, UK	ab8592	IHC; IF	1/200
E-cadherin	mouse anti-E-cadherin	Mouse MAb, clone 36/E-cadherin	BD Transduction Laboratories, San Jose, CA, USA	610182	IHC	1/500
GFAP	rabbit anti-glial fibrillary acidic protein	Rabbit PAb, IgG fraction	Agilent-Dako, Santa Clara, CA, USA	Z0334	IHC	1/5000
NFAT1c	NFAT1c antibody	Mouse MAb, 7A6	Santa Cruz Biotechnology, Heidelberg, Germany	sc-7294	IHC	1/6000
p120 catenin	mouse anti-p120 catenin	Mouse MAb, clone 98/pp120	BD Transduction Laboratories, San Jose, CA, USA	610134	IHC	1/200
Sox2	Recombinant anti-Sox2	Rabbit MAb, EPR3131	Abcam, Cambridge, UK	ab92494	IHC	1/200
Vimentin	Anti-vimentin	Guinea pig, PAb	Fitzgerald Industries International, Acton, MA, USA	20R-VP004	IHC	1/6000
Beta-tubulin	Beta-tubulin antibody #2146	Rabbit, Pab	Cell Signaling Technology	2146S	WB	1/1000

[a] WB, Western blotting; IHC, immunohistochemistry; IF, immunofluorescence
