# Supplementary material for: Innovative mouse models for the tumor suppressor activity of Protocadherin-10 isoforms
Source: BMC Cancer. 2022 Apr 25;22:451. doi: 10.1186/s12885-022-09381-y (PMC9040349; doi:10.1186/s12885-022-09381-y)
Supplement: Supplementary file 8 — Additional file 8: Table S7. Protocadherin-10 specific antibodies used. [file 12885_2022_9381_MOESM8_ESM.pdf]

Kleinberger, Sanders, Staes et al. (2022)

Additional file 8: Table S7. Protocadherin-10 specific antibodies used

Antibody name	Antigen	Epitope AA	Antibody type	Source[a]	Isoform specificity	Applications[b]	Working dilution	Immunizing peptide (NH2->COOH)	Reference[c]
2H8	mPcdh10iso1	799-896	Rat monoclonal	S. Hirano	all isoforms	WB, IHC, IF	1/200	PAQVPVEE…SESLL	Hirano et al. 1999
5G10	mPcdh10iso3	878-1040	Rat monoclonal	S. Hirano	long isoforms only	WB, IHC, IF	1/200	NKHQRAE…RKRIC	Aoki et al. 2003
G415	h/mPCDH10	1025-1040	Rabbit polyclonal	DBMB, UGent	long isoforms only	WB	1/500	TRAPYKPPYLTRKRIC	
G418	mPcdh10	878-892	Rabbit polyclonal	DBMB, UGent	short isoform only	WB	1/500	VRLKGKKHHLSSPPS	
PA5-31043	hPCDH10	19-308	Rabbit polyclonal	Invitrogen	all isoforms	FACS	1/1000	MIVLLL….GEVVYSFS	

**mPcdh10 domains**	**AA**
TM	716-736
Mature short iso 1	19-896
Mature long iso 2	19-1047
Mature long iso 3	19-1040
Mature long iso 4	19-1057
Short-specific CD	878-896
Long-specific CD	878-1040/1057

[a] DBMB: Department of Biomedical Molecular Biology, Ghent University, Ghent 9052, Belgium

[b] WB, Western blotting; IHC, immunohistochemistry; IF, immunofluorescence; FACS, fluorescence activated cell sorting

[c] References:

Hirano S, Yan Q, Suzuki ST. Expression of a novel protocadherin, OL-protocadherin, in a subset of functional systems of the developing mouse brain. J Neurosci. 1999;19:995-1005.

Aoki E, Kimura R, Suzuki ST, Hirano S. Distribution of OL-protocadherin protein in correlation with specific neural compartments and local circuits in the postnatal mouse brain. Neuroscience. 2003;117:593-614.
