# Supplementary material for: Innovative mouse models for the tumor suppressor activity of Protocadherin-10 isoforms
Source: BMC Cancer. 2022 Apr 25;22:451. doi: 10.1186/s12885-022-09381-y (PMC9040349; doi:10.1186/s12885-022-09381-y)
Supplement: Supplementary file 12 — Additional file 12: Fig. S4. mRNA expression levels of δ-Pcdhs in Pcdh10﻿all−/− and Pcdh10long−/− mouse brains. [file 12885_2022_9381_MOESM12_ESM.pdf]

Additional file 12 for Kleinberger, Sanders, Staes et al. (2022)

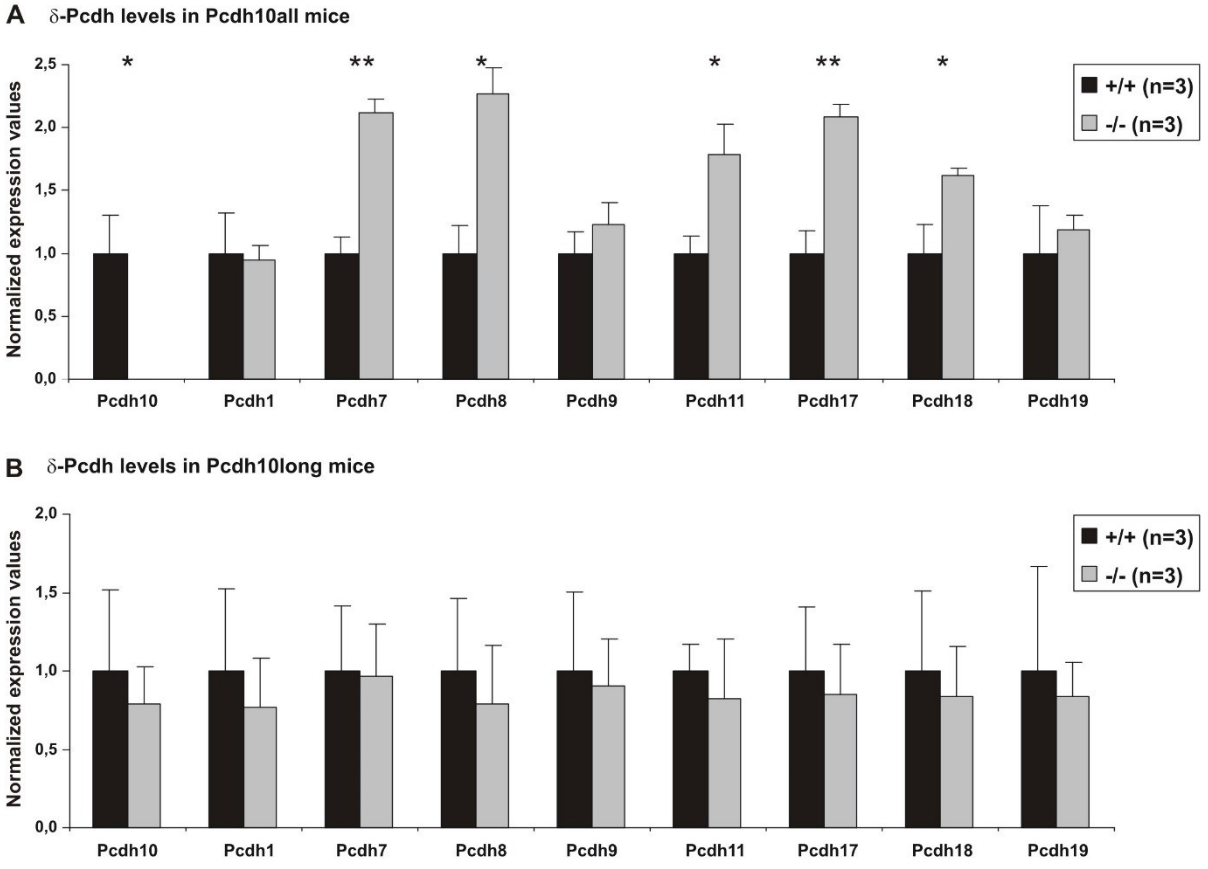

Additional Figure S4. mRNA expression levels of δ-Pcdhs in Pcdh10all$^{-/-}$ and Pcdh10long$^{-/-}$ mouse brains. **A** qRT-PCR analysis of brains from Pcdh10all$^{+/+}$ and Pcdh10all$^{-/-}$ mice using primers specific for all isoforms of various δ-Pcdh genes. Pcdh7, Pcdh8, Pcdh11, Pcdh17 and Pcdh18 showed a statistically significant upregulation in Pcdh10all$^{-/-}$ brains. **B** qRT-PCR analysis of brains from Pcdh10long$^{+/+}$ and Pcdh10long$^{-/-}$ mice using primers specific for all isoforms of various δ-Pcdh genes. None of the δ-Pcdh genes showed a differential expression in Pcdh10long$^{-/-}$ brains. Statistical analyses were performed using an unpaired t-test, * $p < 0.05$, ** $p < 0.01$. See Additional file 6 (Table S5) for primer information.
