# Supplementary material for: Innovative mouse models for the tumor suppressor activity of Protocadherin-10 isoforms
Source: BMC Cancer. 2022 Apr 25;22:451. doi: 10.1186/s12885-022-09381-y (PMC9040349; doi:10.1186/s12885-022-09381-y)
Supplement: Supplementary file 13 — Additional file 13: Fig. S5. Phenotypic and reproduction data for Pcdh10﻿all−/− and Pcdh10long−/− knockout (KO) mice. [file 12885_2022_9381_MOESM13_ESM.pdf]

Additional file 13 for Kleinberger, Sanders, Staes et al. (2022)

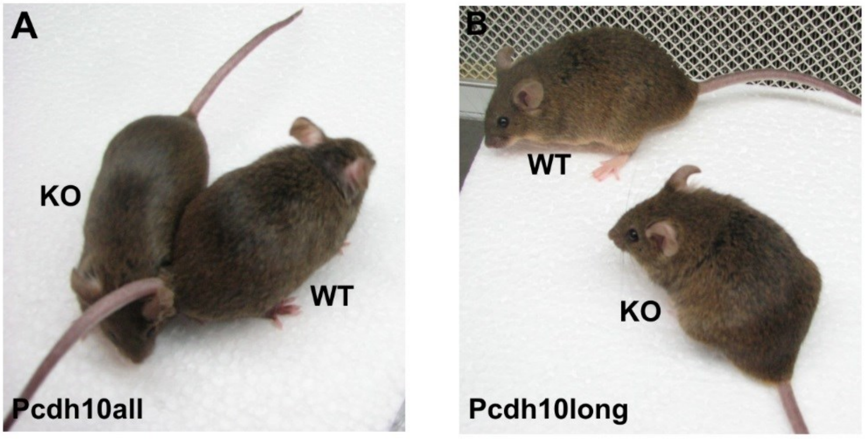

C Pcdh10all

	# males	# females	Total
+/+	24	20	44
+/-	44	57	101
-/-	16	19	35
Total	84	96	180

D Pcdh10long

	# males	# females	Total
+/+	12	16	28
+/-	31	23	54
-/-	24	19	43
Total	67	58	125

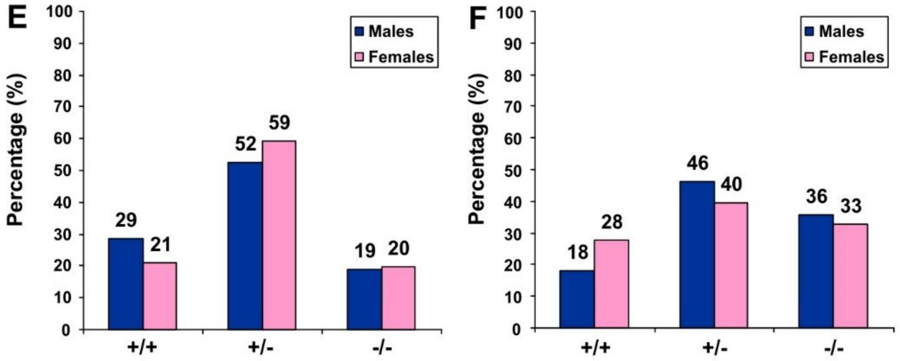

Additional Fig. S5. Phenotypic and reproduction data for Pcdh10all$^{-/-}$ and Pcdh10long$^{-/-}$ knockout (KO) mice. Both Pcdh10all$^{fl/fl}$ and Pcdh10long$^{fl/fl}$ mice were crossed with a transgenic mouse line showing ubiquitous expression of Cre recombinase in all tissues (Nestin-Cre). **A,B** General appearance of Pcdh10all$^{-/-}$ (9 month old females) and Pcdh10long$^{-/-}$ (11 month old males) mice, respectively. KO mice are indistinguishable from their WT littermates. **C,D** Number of, respectively, wild-type (+/+), heterozygous (+/-) and homozygous (-/-) Pcdh10all and Pcdh10long mice born. Separate numbers are given for male and female pups. **E,F** Graphical representation of Mendelian ratios of, respectively, Pcdh10all (**E**) and Pcdh10long (**F**) KO mice born. Separate numbers are given for male and female mice.
