# Supplementary material for: Innovative mouse models for the tumor suppressor activity of Protocadherin-10 isoforms
Source: BMC Cancer. 2022 Apr 25;22:451. doi: 10.1186/s12885-022-09381-y (PMC9040349; doi:10.1186/s12885-022-09381-y)
Supplement: Supplementary file 14 — Additional file 14﻿: Fig. S6. Body weight curves of Pcdh10all−/− and Pcdh10long−/− KO mice. [file 12885_2022_9381_MOESM14_ESM.pdf]

Additional file 14 for Kleinberger, Sanders, Staes et al. (2022)

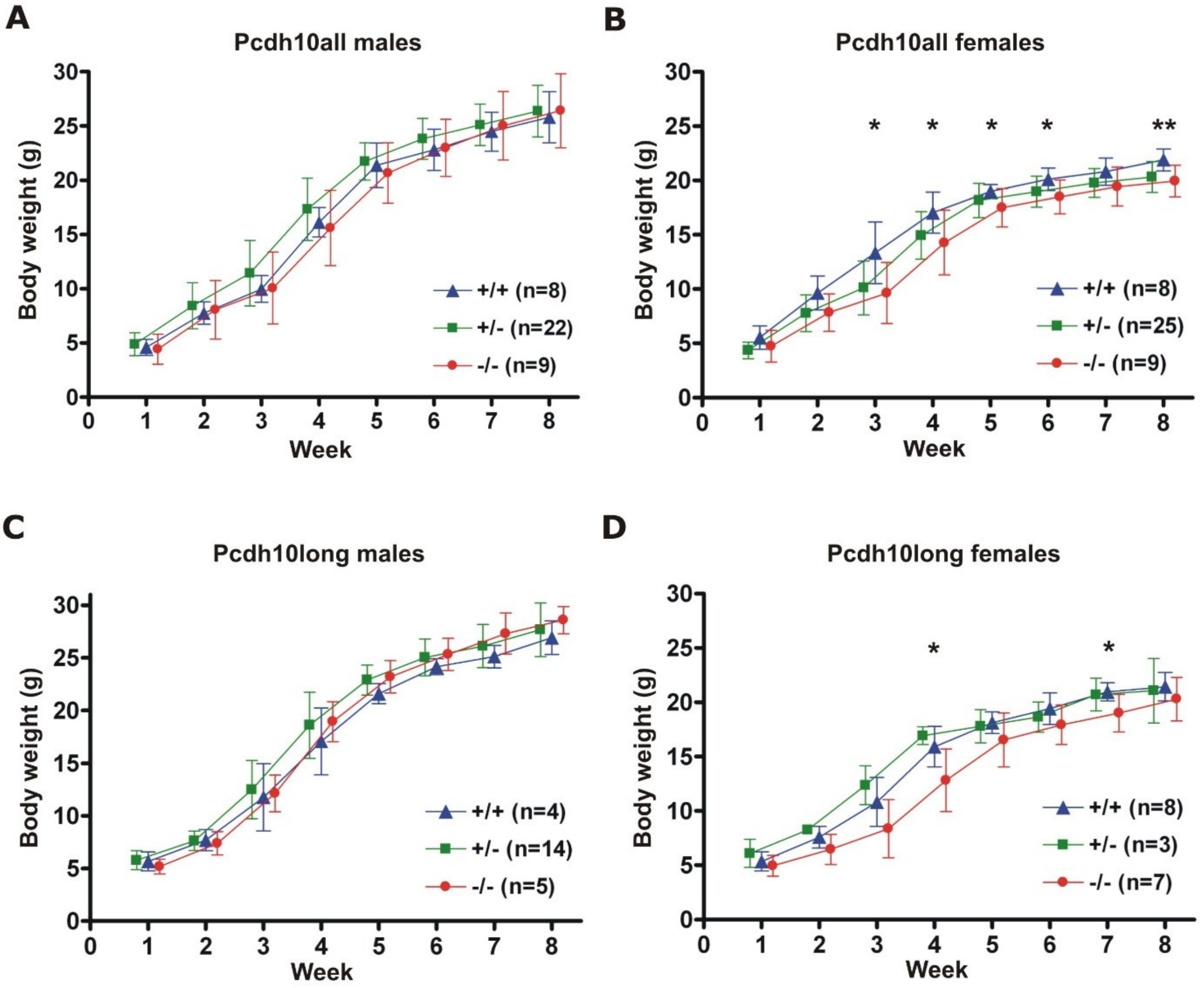

Additional Figure S6. Body weight curves of Pcdh10all$^{-/-}$ and Pcdh10long$^{-/-}$ KO mice. Body weight was recorded for 8 weeks after birth for (**A**) Pcdh10all$^{-/-}$ males, (**B**) Pcdh10all$^{-/-}$ females, (**C**) Pcdh10long$^{-/-}$ males and (**D**) Pcdh10long$^{-/-}$ females. Graphs of different genotypes are slightly shifted for easier interpretation. Statistical analysis of wild-type (+/+), heterozygous (+/-) or homozygous (-/-) KO mice over a time period of 8 weeks using one-way ANOVA did not show a statistically significant difference (Pcdh10all males, $p = 0.9778$; Pcdh10all females, $p = 0.7853$; Pcdh10long males, $p = 0.9712$; Pcdh10long females, $p = 0.7848$).
