# Supplementary material for: Innovative mouse models for the tumor suppressor activity of Protocadherin-10 isoforms
Source: BMC Cancer. 2022 Apr 25;22:451. doi: 10.1186/s12885-022-09381-y (PMC9040349; doi:10.1186/s12885-022-09381-y)
Supplement: Supplementary file 15 — Additional file 15: Table S11. Statistical analysis of Kaplan Meyer tumor-free curves for GFAP-Cre experiment. [file 12885_2022_9381_MOESM15_ESM.pdf]

Kleinberger, Sanders, Staes et al. (2022)

Additional file 15: Table S11. Statistical analysis of Kaplan Meyer tumor-free survival curves for GFAP-Cre experiments[a]

Pcdh10all expt	Genotypes				# of	Statistical significance[b]
**Tumor type**	GFAP-Cre	**Pcdh10all**	p53	Rb	**animals**	**(P-value)**
**Medulloblastoma**	tg/+	fl/fl vs +/+	fl/fl	fl/fl	68 vs 61	*** (0.0003)
	tg/+	fl/fl vs fl/+	fl/fl	fl/fl	68 vs 52	** (0.0085)
	tg/+	fl/fl	fl/fl	fl/fl vs +/+	68 vs 48	**** (<0.0001)
	tg/+	fl/fl	fl/fl vs +/+	fl/fl	68 vs 16	**** (<0.0001)
**Auricular tumors**	tg/+	fl/fl vs +/+	fl/fl	fl/fl	69 vs 61	* (0.0230)
	tg/+	fl/fl	fl/fl	fl/fl vs +/+	69 vs 48	**** (<0.0001)
	tg/+	fl/fl vs +/+	fl/fl	+/+	48 vs 27	** (0.0022)
	tg/+	fl/fl	fl/fl vs +/+	+/+	48 vs 23	** (0.0033)

Pcdh10long expt	Genotypes				# of	Statistical significance
**Tumor type**	GFAP-Cre	**Pcdh10long**	p53	Rb	**animals**	**(P-value)**
**Medulloblastoma**	tg/+	fl/fl vs +/+	fl/fl	fl/fl	50 vs 61	** (0.0026)
	tg/+	fl/fl	fl/fl	fl/fl vs +/+	50 vs 28	**** (<0.0001)
	tg/+	fl/fl	fl/fl	fl/fl vs fl/+	50 vs 38	**** (<0.0001)
	tg/+	fl/fl	fl/fl	fl/+ vs +/+	38 vs 28	*** (0.0004)
**Auricular tumors**	tg/+	fl/fl vs +/+	fl/fl	fl/fl	50 vs 61	ns (0.0536)
	tg/+	fl/fl	fl/fl	fl/fl vs +/+	50 vs 28	**** (<0.0001)
	tg/+	fl/fl vs +/+	fl/fl	+/+	28 vs 27	ns (0.0880)

[a] See figures 5 and 6 for the tumor-free survival curves

[b] Log-rank values calculated according to Mantel-Cox
