# Supplementary material for: Innovative mouse models for the tumor suppressor activity of Protocadherin-10 isoforms
Source: BMC Cancer. 2022 Apr 25;22:451. doi: 10.1186/s12885-022-09381-y (PMC9040349; doi:10.1186/s12885-022-09381-y)
Supplement: Supplementary file 16 — Additional file 16: Fig. S7. Examples of validation of antibody specifity in immunohistochemical detection of various antigens on mouse WT and tumoral tissues. [file 12885_2022_9381_MOESM16_ESM.pdf]

Additional file 16 for Kleinberger, Sanders, Staes et al. (2022)

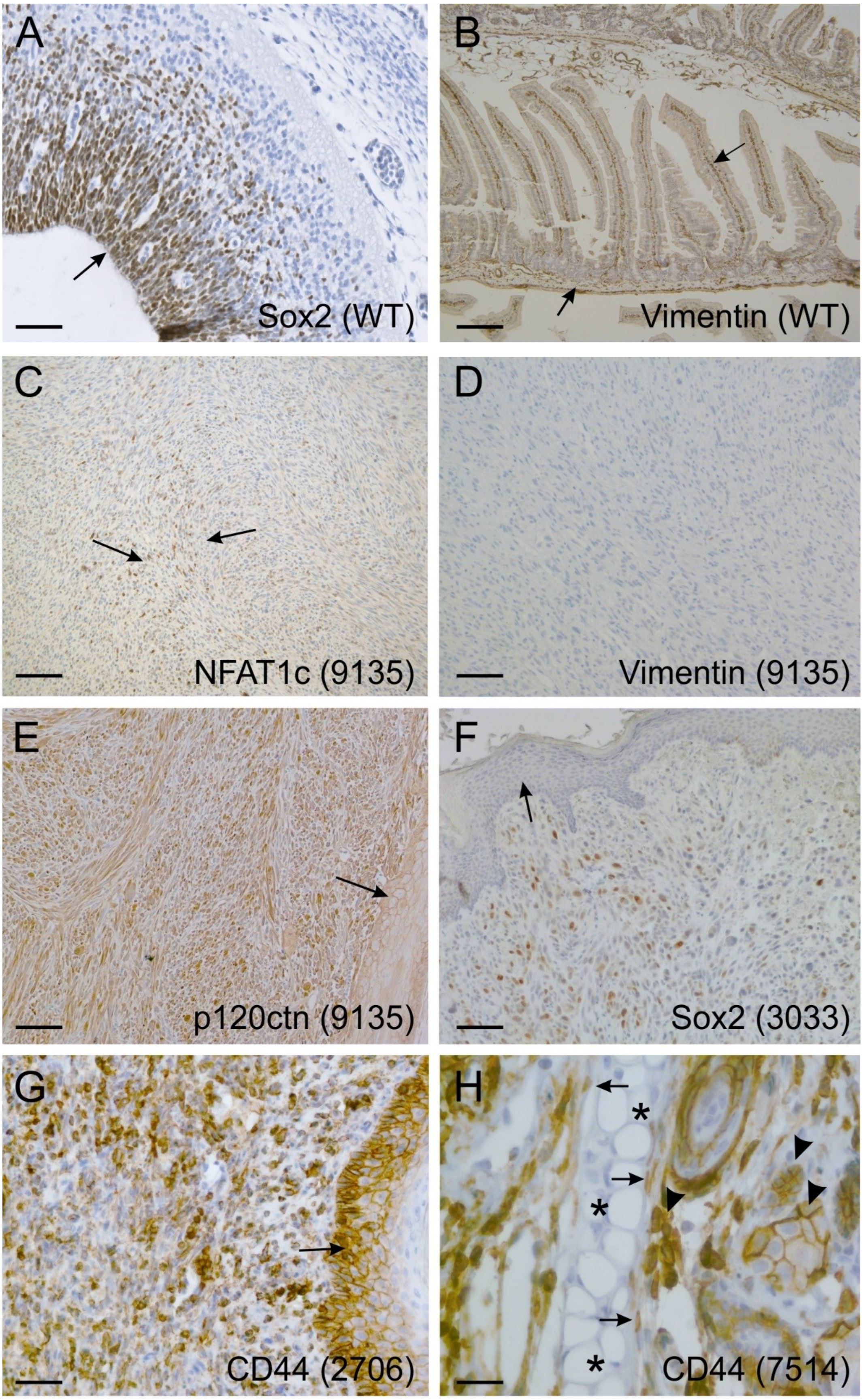

Additional Figure S7. Examples of validation of antibody specifity in immunohistochemical detection of various antigens on mouse WT and tumoral tissues. Both reliable antibody source (company, catalogue number, batch number) and working dilutions were optimized in such pilot experiments. See Additional file 7: Table S6 for final choices. Unspecific binding by secondary antibodies was checked by omitting the primary antibody (not shown). **A** Specific nuclear expression pattern of Sox2 in the ventricular zone of WT embryonic mouse brain (E13.5) (arrow). **B** Specific cytoplasmic expression pattern of vimentin in the mesenchymal cells of adult mouse intestine (arrows). **C-E** Serial sections of a pinnal tumor were stained for nuclear NFAT1c (**C**), cytoplasmic vimentin (**D**) or p120ctn staining, expressed at cell-cell borders (epidermis, arrow) or in the cytoplasm and nuclei (tumor) (**E**). The p120ctn catenin is about ubiquitously expressed and its staining serves also as check for the normal immunoreactivity of the tissue (defective in for instance necrotic or poorly processed tissues). This particular tumor shows strong NFATc (arrows in **C**) and vimentin reactivity in part of the tumor but lacks such reactivity in other parts, which were nonetheless positive for p120ctn staining. **F** Nuclear staining of Sox2 in part of the nuclei of a pinnal tumor. Other tumoral nuclei and normal tissues (epidermis; arrow) were negative. **G** Cell surface staining of CD44 in pinnal tumor cells and in the basal layers of normal epidermis (arrow). **H** Cell surface staining of CD44 in pinnal tumor cells (arrowheads) and in perichondrial stem cells (arrows), but not in cells of differentiated pinnal elastic cartilage (asterisks). The genotype of mice in (**C-H**) is GFAP-Cre$^{tg/+}$;Pcdh10all$^{fl/fl}$;p53$^{fl/fl}$;Rb$^{+/+}$. Mouse ear tag numbers are given between brackets. Scale bars: 50 μm in **A**, **F** and **G**; 200 μm in **B**; 100 μm in **C-E**, 25 μm in **H**.
