# Supplementary material for: Innovative mouse models for the tumor suppressor activity of Protocadherin-10 isoforms
Source: BMC Cancer. 2022 Apr 25;22:451. doi: 10.1186/s12885-022-09381-y (PMC9040349; doi:10.1186/s12885-022-09381-y)
Supplement: Supplementary file 17 — Additional file 17: Fig. S8. Immunohistochemical detection of E-cadherin and catenins in representative pinnal tumors. [file 12885_2022_9381_MOESM17_ESM.pdf]

Additional file 17 for Kleinberger, Sanders, Staes et al. (2022)

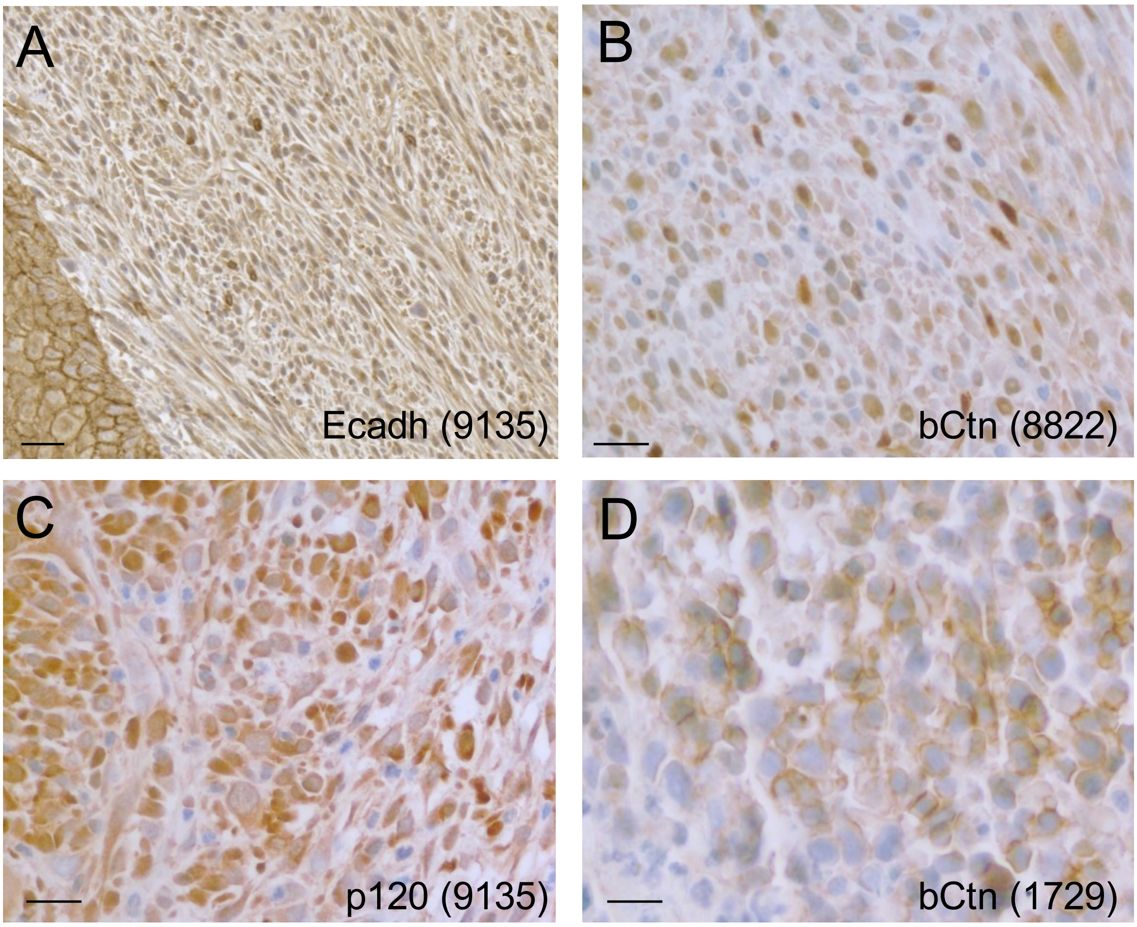

Additional Figure S8. Immunohistochemical detection of E-cadherin and catenins in representative pinnal tumors. **A** cytoplasmic E-cadherin staining (lower left corner: normal epidermis with cell contact staining). **B** Nuclear beta-catenin staining. **C** Cytoplasmic and nuclear p120 catenin staining. **D** Beta-catenin staining at cell-cell contacts. The genotype of mice in (**A-C**) is GFAP-Cre$^{tg/+}$;Pcdh10all$^{fl/fl}$;p53$^{fl/fl}$;Rb$^{+/+}$. The mouse in (**D**) has genotype GFAP-Cre$^{tg/+}$;Pcdh10all$^{fl/fl}$;p53$^{fl/fl}$;Rb$^{fl/fl}$. Mouse ear tag numbers are given between brackets. Scale bars: 25 μm.
