# Supplementary material for: Innovative mouse models for the tumor suppressor activity of Protocadherin-10 isoforms
Source: BMC Cancer. 2022 Apr 25;22:451. doi: 10.1186/s12885-022-09381-y (PMC9040349; doi:10.1186/s12885-022-09381-y)
Supplement: Supplementary file 18 — Additional file 18: Table S12. Derivation and detailed analysis of pinnal-tumor derived (PTD) cell lines and rescued derivatives. [file 12885_2022_9381_MOESM18_ESM.pdf]

Kleinberger, Sanders, Staes et al. (2022)

Additional file 18: Table S12 Derivation and detailed analysis of pinnal-tumor derived (PTD) cell lines and rescued derivatives

Mouse tag number	**Genotype of original mouse with pinnal tumor**	**Derivation**	**PTD cell line**	Achorage-independent growth in soft agar	Invasive growth (fold increase perimeter at 92h)*	Allograft from 2.5 x 10^6 cells (days to reach 1 cm^3)	Allograft from 1 x 10^6 cells (days to reach 1 cm^3)	Allograft from 1 x 10^5 cells (days to reach 1 cm^3)	Allograft from 1 x 10^4 cells (days to reach 1 cm^3)	Allograft from 1 x 10^3 cells (days to reach 1 cm^3)
9034	GFAP-$Cre^{tg/+}$;$Pcdh10all^{fl/fl}$;$p53^{fl/fl}$;$Rb^{fl/fl}$	parental population	PTD4	++	4.29	18.0	n.d.	n.d.	n.d.	n.d.
~	~	subclone	PTD8	++	4.87	23.5	n.d.	n.d.	n.d.	n.d.
~	~	subclone	**PTD7**	++	3.77	19.5	23.7-24.9	29.5-32.9	> 35	n.d.
~	**PTD7 rescued with Pcdh10 short isoform**	transduced and FACS sorted	**PTD7_RS**	neg.	**1.50**	n.d.	**29.0**	**36.6**	n.d.	n.d.
~	**PTD7 rescued with Pcdh10 long isoform**	transduced and FACS sorted	**PTD7_RL**	neg.	**1.50**	n.d.	**32.7**	**36.2**	n.d.	n.d.
8822	GFAP-$Cre^{tg/+}$;$Pcdh10all^{fl/fl}$;$p53^{fl/fl}$;$Rb^{+/+}$	subclone	PTD11	neg.	2.69	31.8	36.7	38.5	40.0	n.d.
~	~	subclone	PTD12	neg.	2.90	50.0	n.d	n.d	n.d	n.d.
~	~	PTD12 allograft derivation	PTD12AD4	n.d.	n.d.	n.d.	23.7	n.d.	n.d.	n.d.
9135	GFAP-$Cre^{tg/+}$;$Pcdh10all^{fl/fl}$;$p53^{fl/fl}$;$Rb^{+/+}$	subclone	PTD17	neg.	n.d.	22.6	n.d.	n.d.	n.d.	n.d.
~	~	subclone	PTD19	neg.	n.d.	27.0	31.0	38.4	41.2	n.d.
1729	GFAP-$Cre^{tg/+}$;$Pcdh10all^{fl/fl}$;$p53^{fl/fl}$;$Rb^{fl/fl}$	subclone	PTD24	++	n.d.	10.0	n.d.	n.d.	n.d.	n.d.
~	~	subclone	**PTD25**	+++	3.81	12.7	n.d.	17.5-20.8	24.9-28.0	27.5
~	**PTD25 rescued with Pcdh10 short isoform**	transduced and FACS sorted	**PTD25_RS**	neg.	**0.95**	n.d.	n.d.	**no tumor**	**no tumor**	n.d.
~	**PTD25 rescued with Pcdh10 long isoform**	transduced and FACS sorted	**PTD25_RL**	neg.	**0.64**	n.d.	n.d.	**34.0**	**39.4**	n.d.
9988	GFAP-$Cre^{tg/+}$;$Pcdh10all^{fl/fl}$;$p53^{fl/fl}$;$Rb^{fl/+}$	subclone	PTD26	n.d.	3.24	23.5	n.d.	n.d.	n.d.	n.d.
~	~	subclone	PTD27	n.d.	2.21	19.5	n.d.	n.d.	n.d.	n.d.
n.a.	n.a. (human osteosarcoma cell line)	Negative control cell line	HOS	neg.	n.d.	**no tumor**	n.d.	n.d.	n.d.	n.d.
n.a.	n.a. (human breast cancer cell line)	Negative control cell line	T47D	n.d.	**1.20**	n.d.	n.d.	n.d.	n.d.	n.d.
n.a.	n.a. (chemically transformed HOS cell line)	Positive control cell line	MNNG-HOS	+++	4.78	23.4	n.d.	n.d.	n.d.	n.d.

* Fold increase in perimeter containing ~ 90 % invasive cells; in vitro outgrowth at 92 h after seeding spheroids in 3-dimensional matrix of collagen (1 mg/ml).
