# Supplementary material for: Innovative mouse models for the tumor suppressor activity of Protocadherin-10 isoforms
Source: BMC Cancer. 2022 Apr 25;22:451. doi: 10.1186/s12885-022-09381-y (PMC9040349; doi:10.1186/s12885-022-09381-y)
Supplement: Supplementary file 19 — Additional file 19: Fig. S9. Allograft formation after s.c. injection of PTD single-cell suspensions into athymic nude mice. [file 12885_2022_9381_MOESM19_ESM.pdf]

Additional file 19 for Kleinberger, Sanders, Staes et al. (2022)

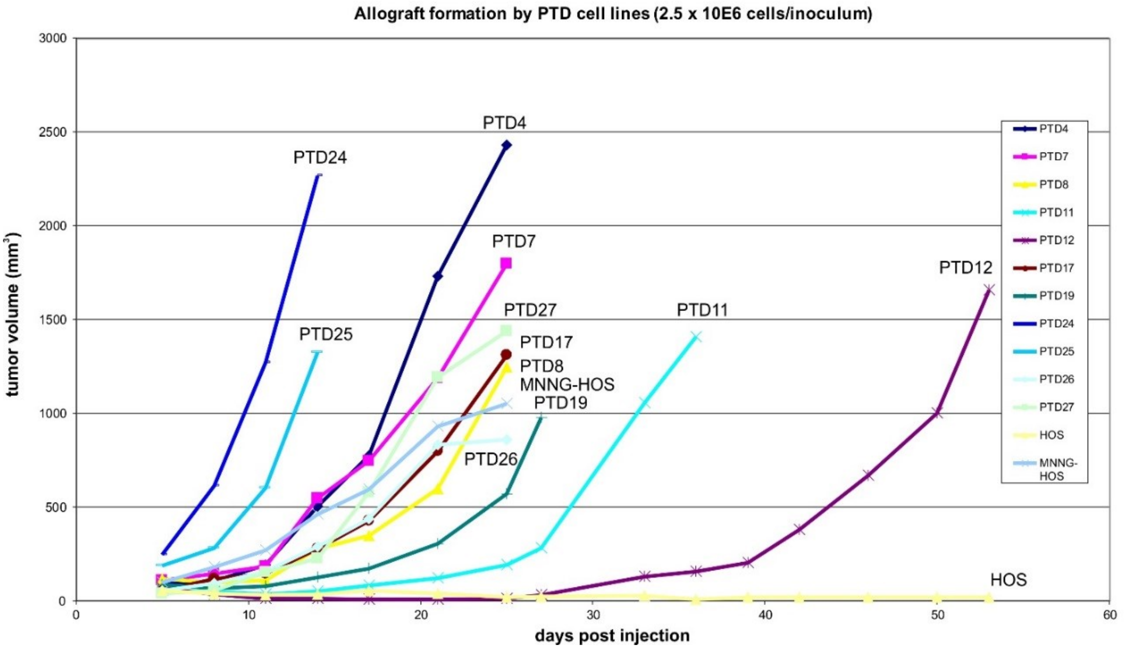

Fig. S9 Allograft formation after s.c. injection of PTD single-cell suspensions into athymic nude mice. Genotypes of the original pinnal-tumor bearing mice (Table 1) were: GFAP-$Cre^{tg/+}$;$Pcdh10all^{fl/fl}$;$p53^{fl/fl}$;$Rb^{fl/fl}$ (for PTD4, 7, 8, 24, 25), GFAP-$Cre^{tg/+}$;$Pcdh10all^{fl/fl}$;$p53^{fl/fl}$;$Rb^{fl/+}$ (for PTD26, 27) and GFAP-$Cre^{tg/+}$;$Pcdh10all^{fl/fl}$;$p53^{fl/fl}$;$Rb^{+/+}$ (for PTD11, 12, 17, 19). The human cell lines HOS and MNNG-HOS served as negative and positive controls, respectively. The number of inoculated cells per mouse was $2.5x10^6$. The cells were mixed with Matrigel before injection. Tumor volumes are the average for 5 injected mice. See Additional file 18: Table S12 for quantitation.
