# Supplementary material for: Innovative mouse models for the tumor suppressor activity of Protocadherin-10 isoforms
Source: BMC Cancer. 2022 Apr 25;22:451. doi: 10.1186/s12885-022-09381-y (PMC9040349; doi:10.1186/s12885-022-09381-y)
Supplement: Supplementary file 20 — Additional file 20. Lung colonization assay for selected PTD cell lines. Includes Table S13 and Fig. S10. [file 12885_2022_9381_MOESM20_ESM.pdf]

Additional file 20 for Kleinberger, Sanders, Staes et al. (2022)

Additional Table S13. Lung colonization assay for selected PTD cell lines

Inoculum*	Mouse	**2 wk PI**	**3 wk PI**	**4 wk PI**	**6 wk PI**	**8 wk PI**
PTD7	#1	lungs OK	n.a.	n.a.	n.a.	n.a.
	#2		2 red spots	n.a.	n.a.	n.a.
	#3			9 lung nodules	n.a.	n.a.
	#4				20 lung nodules	n.a.
	#5				14 lung nodules	n.a.
	#6				12 nodules + 2 on ribs	n.a.
	#7				14 nodules + 2 on ribs	n.a.
PTD11	#1	lungs OK			n.a.	n.a.
	#2		lungs OK		n.a.	n.a.
	#3			lungs OK	n.a.	n.a.
	#4					lungs OK
	#5					lungs OK
	#6					lungs OK
	#7					lungs OK
PTD19	#1	lungs OK			n.a.	n.a.
	#2		lungs OK		n.a.	n.a.
	#3			lungs OK	n.a.	n.a.
	#4					lungs OK
	#5					lungs OK
	#6					lungs OK
	#7					lungs OK
PTD25	#1	2 red spots		n.a.	n.a.	n.a.
	#2		5 lung nodules	n.a.	n.a.	n.a.
	#3			15 lung nodules	n.a.	n.a.
	#4			18 lung nodules	n.a.	n.a.
	#5			> 7 lung nodules#	n.a.	n.a.
	#6			5 lung nodules	n.a.	n.a.
	#7			died prematurely	n.a.	n.a.

* For each PTD cell line, $2x10^5$ viable cells were injected into the tail vein of athymic mice (6-wk old). Mice were killed at the times indicated (wk PI: weeks after i.v. injection) and lungs were examined by stereo microscropy and HE staining of serial paraffin sections. Genotypes of PTD7 and PTD25 were GFAP-$Cre^{tg/+}$;$Pcdh10all^{fl/fl}$;$p53^{fl/fl}$;$Rb^{fl/fl}$; genotypes of PTD11 and PTD19 were GFAP-$Cre^{tg/+}$;$Pcdh10all^{fl/fl}$;$p53^{fl/fl}$;$Rb^{+/+}$.

\# including a big tumor connecting lung lobes

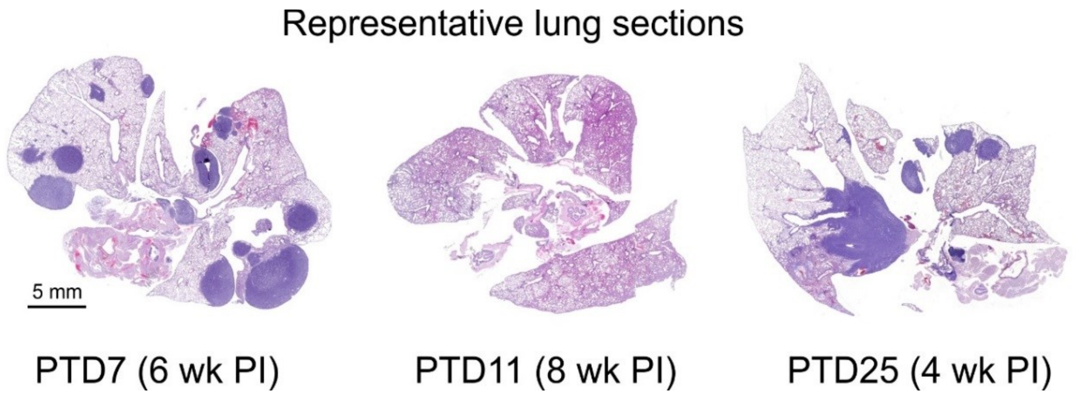

Additional Fig. S10. Representative paraffin sections of lung lobes of i.v. injected athymic mice were stained with HE. The cell lines injected and the incubation period after i.v. injection are given. Scale bar: 5 mm.
