# Supplementary material for: Innovative mouse models for the tumor suppressor activity of Protocadherin-10 isoforms
Source: BMC Cancer. 2022 Apr 25;22:451. doi: 10.1186/s12885-022-09381-y (PMC9040349; doi:10.1186/s12885-022-09381-y)
Supplement: Supplementary file 21 — Additional file 21: Fig. S11. Immunofluorescent detection of desmin in PTD cell cultures and derivatives. [file 12885_2022_9381_MOESM21_ESM.pdf]

Additional file 21 for Kleinberger, Sanders, Staes et al. (2022)

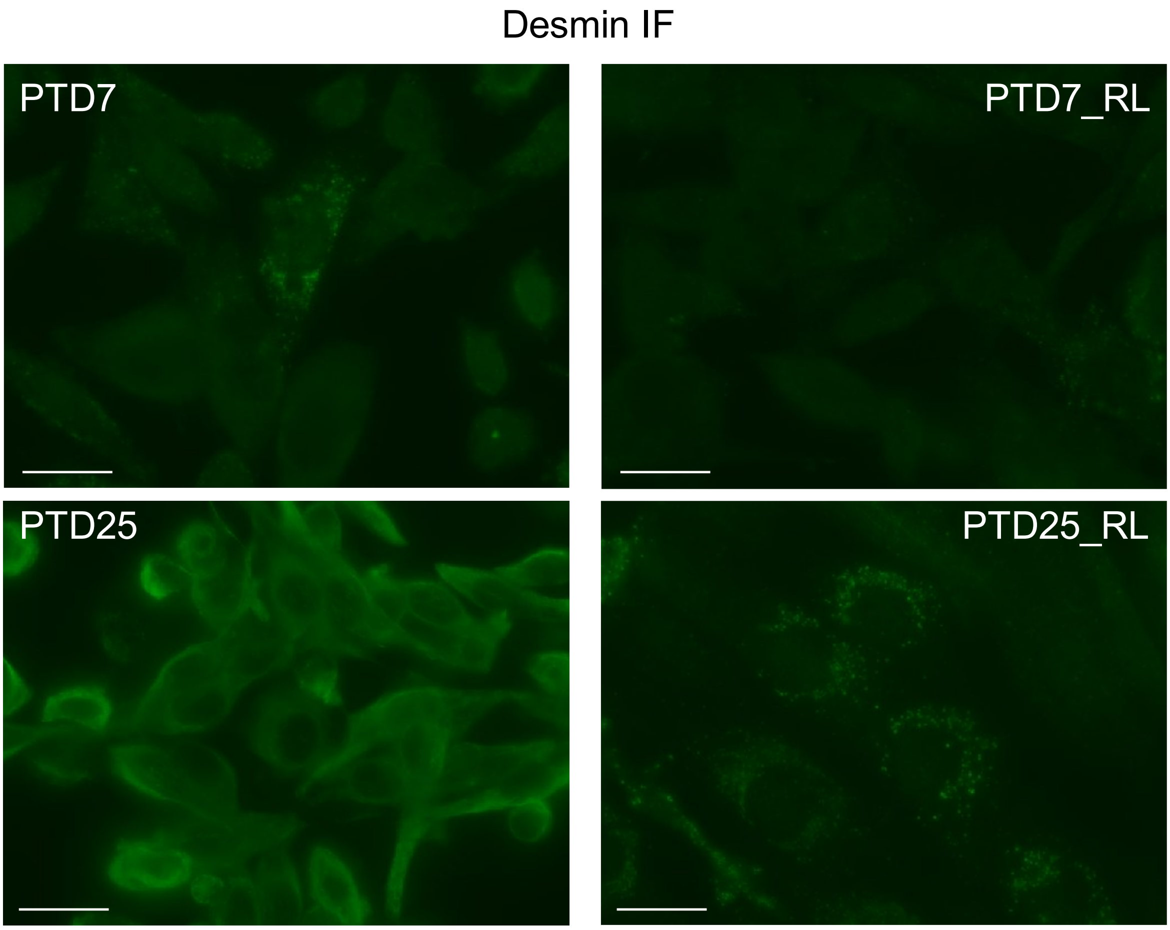

Fig. S11 Immunofluorescent detection of desmin in PTD cell cultures and derivatives. Only PTD25 is clearly positive. PTD7_RL and PTD25_RL are transduced and FACS-sorted derivative populations of, respectively, PTD7 and PTD25, rescued for Pcdh10 ablation by cDNA expression of Pcdh10_long isoform-4 (see text, Table 1 and Additional file 18: Table S12). Scale bars: 40 µm.
