# Supplementary material for: Innovative mouse models for the tumor suppressor activity of Protocadherin-10 isoforms
Source: BMC Cancer. 2022 Apr 25;22:451. doi: 10.1186/s12885-022-09381-y (PMC9040349; doi:10.1186/s12885-022-09381-y)
Supplement: Supplementary file 22 — Additional file 22: Table S14. Statistical analysis of allograft growth curves for PTD7, PTD25 and their Pcdh10-rescued derivative cell lines. [file 12885_2022_9381_MOESM22_ESM.pdf]

Kleinberger, Sanders, Staes et al. (2022)

Additional file 22: Table S14. Statistical analysis of allograft growth curves for PTD7, PTD25 and their Pcdh10-rescued derivative cell lines

Statistical p-values for pairwise comparisons using type II ANOVA							
				F test (KR)	Wald test		Conclusion
Cell line #1	Inoculum	Cell line #2	Inoculum				
PTD7	10E6	PTD7_RS	10E6	p<0.11	p<0.06		ns
PTD7	10E5	PTD7_RS	10E5	p<0.0044	p<<0.0001		**->***
PTD7	10E6	PTD7_RL	10E6	p<0.031	p<0.0048		*->**
PTD7	10E5	PTD7_RL	10E5	p<0.013	p<0.0008		*->***
PTD25	10E5	PTD25_RS	10E5	p<0.0011	p<<0.0001		**->***
PTD25	10E4	PTD25_RS	10E4	p<0.0020	p<<0.0001		**->***
PTD25	10E5	PTD25_RL	10E5	p<0.0017	p<<0.0001		**->***
PTD25	10E4	PTD25_RL	10E4	p<0.0050	p<0.0001		**->***
*: p<0.05							
**: p<0.01							
***: p<0.001							
