# Supplementary material for: Innovative mouse models for the tumor suppressor activity of Protocadherin-10 isoforms
Source: BMC Cancer. 2022 Apr 25;22:451. doi: 10.1186/s12885-022-09381-y (PMC9040349; doi:10.1186/s12885-022-09381-y)
Supplement: Supplementary file 23 — Additional file 23: Table S15. Expression of 134 selected genes in PTD7, PTD25 and derivatives [file 12885_2022_9381_MOESM23_ESM.pdf]

Kleinberger,Sanders, Staes et al. (2022)

Additional file 23: Table S15. Expression of 134 selected genes in PTD7, PTD25 and derivatives[a]

	Mean of 3	Mean of 4	log2FoldChange	padj	Mean of 4	log2FoldChange	padj		Mean of 3	Mean of 4	log2FoldChange	padj	Mean of 3	log2FoldChange	padj
	PTD7	PTD7_RS	DE PTD7_RS vs PTD7		PTD7_RL	DE PTD7_RL vs PTD7			PTD25	PTD25_RS	DE PTD25_RS vs PTD25		PTD25_RL	DE PTD25_RL vs PTD25	
**Cadherins and protocadherins**															
Cdh1 (E-cadherin)	0,8	0,9			0,9				14,2	5,8			5,7		
Cdh2 (N-cadherin)	559,5	697,6			666,0				1273,9	2281,9			1776,9		
Cdh11 (OB-cadherin)	415,8	154,6	-1,37	6,32E-25	69,5	-2,47	1,10E-69		81,6	1235,2	3,88	7,39E-103	389,7	2,23	2,13E-30
Cdh13 (T-cadherin)	383,0	535,5			874,3	1,13	1,12E-12		557,6	1006,1			499,7		
Cdh15 (M-cadherin)	0,3	1,9			0,0				13437,3	612,2	-4,34	3,24E-55	1083,3	-3,53	1,26E-32
Cdh23	10,0	11,7			22,3				202,4	45,1	-2,13	5,57E-20	59,7		
Cdh24	190,2	105,2			122,8				172,7	307,6			257,8		
Cdh26	44,7	73,2			16,7	-1,12	4,20E-05		0,0	0,2			0,7		
Cdhr1	0,5	0,0			0,0				0,0	140,9	6,71	5,98E-27	33,3	4,64	4,79E-13
Pcdh1	30,6	18,8			11,0				918,7	332,2			747,3		
Pcdh7	1248,5	686,7			619,8				623,8	659,0			680,1		
**Pcdh10**	**0,0**	**539,6**	**5,73**	**1,57E-97**	**709,2**	**6,10**	**6,18E-111**		**0,7**	**1415,2**	**8,92**	**8,84E-82**	**3327,0**	**10,14**	**8,16E-105**
Pcdh18	590,4	644,3			1053,7				227,3	853,6			1021,5	2,16	3,17E-59
Pcdh19	793,5	823,8			1135,3				105,8	1014,6	3,24	1,37E-121	1389,4	3,69	9,71E-145
**Catenins**															
Ctnna1 (α-catenin)	3478,6	5060,4			4901,1				11014,8	13519,9			9918,4		
Ctnnb1 (β-catenin)	11658,4	9901,0			10853,3				9035,9	11590,8			11177,1		
Ctnnd1	2626,2	2682,0			2896,9				3737,1	4929,7			4120,2		
Jup	1369,2	1487,5			1357,4				1311,6	250,6	-2,38	1,15E-149	201,3	-2,69	2,46E-149
**Anti-apoptotic**															
Bcl2	71,7	93,2			123,5				19,6	195,5	3,25	3,34E-43	201,6	3,29	9,66E-42
Bcl2l1 (Bcl-Xl)	967,0	808,1			607,6				1416,2	606,3			837,4		
Birc3 (cIAP2)	240,6	224,8			215,2				59,0	143,6			177,9		
Birc5 (Survivin)	2350,6	1567,0			807,2	-1,49	9,03E-37		1449,2	662,3			1037,7		
Mcl1	2512,7	2681,9			2554,4				2404,5	4105,4			4081,5		
Xiap	541,2	783,4			869,2				826,3	1135,6			908,4		
**Pro-Apoptotic**															
Bad	441,1	494,2			449,6				776,5	862,6			1160,2		
Bax	997,1	1015,3			804,6				1204,2	1002,1			1359,5		
Bcl2l13	1134,2	972,0			1031,9				1806,3	1272,4			1322,3		
Bcl6b	205,0	80,1	-1,27	1,23E-14	69,8	-1,45	1,87E-19		864,1	49,0	-4,12	4,22E-181	84,1	-3,34	1,08E-120
Bcl10	1158,4	909,2			914,2				1428,0	1361,3			1306,0		
Bex1	1,1	0,0			0,0				4606,0	2,6	-10,29	4,62E-178	36,0	-6,95	0,00E+00
Bik	1,3	1,2			0,2				89,4	5,8	-3,79	2,99E-27	9,2	-3,19	2,41E-19
Bok	559,1	271,0	-1,01	2,43E-15	304,0				640,8	851,8			652,0		
Casp3	1221,3	1314,3			1133,4				7224,2	1619,0	-2,15	1,96E-141	1332,9	-2,43	1,06E-154
Casp7	50,3	38,4			35,8				521,6	376,8			465,6		
Casp8	392,4	473,9			360,5				388,5	299,6			381,1		
Casp9	381,8	375,0			501,1				492,8	464,2			473,6		
Dapk1	573,0	75,3	-2,80	1,18E-77	19,0	-4,48	3,98E-140		32,6	0,5			0,0		
Dapk2	1,3	0,3			0,0				371,0	13,0			28,0		
Fas	18,4	34,5			41,5				24,5	303,3			390,3		
Fadd	221,0	217,9			133,0				210,9	142,3			160,2		
Fis1	1260,3	1161,4			1003,2				1887,6	1771,0			1929,2		
Lgals7	123,5	201,3			152,5				56,2	2,4			1,4		
Hrk (Harakirin)	22,3	7,1			12,0				10,3	0,0			0,3		

Mrpl41	352,9	370,4			254,3				322,7	303,1			318,9		
Parp1	3145,1	2553,4			2340,3				3582,4	1340,3			1893,9		
Plekhf1	217,6	252,5			394,4				327,9	596,4			584,1		
Plekhf2	195,9	207,5			187,3				274,5	156,2			142,4		
Tnfrsf1b	441,6	465,9			778,5				491,6	1078,5			1849,9		
Tnfrsf12a	6192,4	4147,4			2080,2	-1,53	2,07E-40		9495,0	2804,1			4027,9		
Tnfrsf21	91,8	40,3	-1,06	1,35E-06	47,9				16,7	59,7			21,1		
Tnfrsf4	139,8	135,3			45,7	-1,52	2,65E-24		9,6	4,1			12,7		
Tnfsf12	92,3	66,0			37,9	-1,19	2,41E-10		60,9	231,8			304,1		
Trp53	97,4	75,1			45,6				134,9	56,4			86,3		
**β-catenin/Wnt signaling**															
Bcl9	386,4	417,7			758,0				726,7	612,9			539,3		
Bcl9l	781,7	619,2			1089,9				474,2	1581,5			1467,9		
Ctnnb1 (β-catenin)	11658,4	9901,0			10853,3				9035,9	11590,8			11177,1		
Gsk3b	526,0	514,8			838,8				871,1	1349,8			1566,6		
Hnf1a (Tcf1)	0,8	0,0			0,2				0,3	0,4			1,6		
Lef1	56,8	64,2			131,7				626,5	395,4			113,6		
**NFκB signaling**															
Bcl3	58,3	47,8			113,6				369,9	63,0	-2,54	4,09E-73	77,1	-2,25	1,15E-50
Bcl10	1158,4	909,2			914,2				1428,0	1361,3			1306,0		
Card10	229,4	220,2			428,5				206,8	10,7	-4,18	8,75E-58	13,0	-3,90	5,46E-44
Casp12	143,2	250,1			237,8				45,7	352,2	2,90	3,42E-53	537,1	3,51	7,97E-72
Chuk (IKKalpha)	373,3	533,4			490,2				801,1	647,9			655,9		
Depdc1a	213,0	348,8			264,8				331,3	325,3			233,7		
Icam1	10,6	0,6			0,4				2,1	3,8			4,7		
Ikbkb (IKKbeta)	630,8	577,5			695,2				781,0	857,7			699,2		
Ptgs2 (Cox2)	3142,6	1903,3			1375,7	-1,13	3,11E-15		58,6	180,8			284,5	2,24	2,39E-22
Vegfa	1144,3	1529,1			1787,8				1091,4	1307,4			1412,7		
**PI3K/Akt signaling**															
Akt1	5134,9	3979,2			3677,8				7684,0	4289,3			3571,6		
Pik3ca (p110-α)	955,4	1058,5			1143,8				1089,8	1134,5			838,7		
Pik3cb (P110-β)	500,4	396,3			382,4				263,3	566,2			660,5		
Pik3ip1	299,6	541,0			963,2	1,62	1,97E-34		71,7	854,9	3,54	2,25E-102	566,0	2,95	3,04E-64
Pik3r1 (p85-α)	409,5	382,8			616,0				320,7	1160,3			970,9		
Pik3r2 (p85-β)	1793,3	1487,9			1790,8				3621,2	3699,8			3444,7		
Pik3r3 (p55-γ)	324,3	153,3	-1,05	9,35E-19	160,5				370,2	1281,5			1185,0		
Pten	822,2	1070,8			1266,6				1143,6	3123,4			2498,6		
**Growth factors and receptors**															
Fgfr1	5475,5	3367,2			4032,0				3794,4	4528,7			6347,9		
Vegfa	1144,3	1529,1			1787,8				1091,4	1307,4			1412,7		
Vegfb	558,6	697,9			749,4				1093,5	859,9			1369,7		
**Oncogenes and cell cycle related**															
Bcl6	437,5	363,5			339,0				353,2	368,3			385,9		
Bcl7a	201,3	168,9			227,0				411,7	112,7			117,9		
Ccnd1 (Cyclin D1)	7592,3	5032,9			3393,2	-1,14	8,08E-32		9872,2	4319,7			6487,2		
Ccnd2 (Cyclin D2)	1246,4	550,1	-1,17	4,59E-62	826,8				399,2	964,9			605,1		
Ccnd3 (Cyclin D3)	1497,5	1197,1			973,5				13093,6	2257,5	-2,53	8,97E-237	1972,4	-2,72	1,40E-236
Cdkn1a (p21)	712,2	913,4			873,4				22939,0	954,7			930,4		
Depdc1a	213,0	348,8			264,8				331,3	325,3			233,7		
Ect2	1924,5	2100,1			1416,4				1544,1	941,3			823,0		
Hotair	0,0	0,0			0,0				0,0	0,0			0,0		
Jun	4012,9	1883,4			1016,2	-1,89	9,00E-45		1593,3	1135,3			1899,0		

Malat1	880,1	775,3			2060,2	1,17	7,24E-16		578,1	841,6			2030,0		
Mdm2	457,7	484,9			472,9				624,3	419,7			478,6		
Myc	7442,7	7232,9			5985,6				1692,4	813,1			1404,0		
Tert	32,0	16,3			6,6				31,5	18,7			24,2		
**Tumor/invasion suppressor genes**															
Gadd45a	293,4	992,1			1650,7				1600,9	594,7			754,4		
Gadd45b	395,7	731,4			977,9				303,3	765,1			589,0		
Gadd45g	376,7	275,3			267,3				829,9	1123,4			1113,5		
Gsdmd	220,3	137,6			206,5				717,6	1030,4			793,4		
Htatip2	333,7	234,6			314,8				89,3	110,1			315,3		
Pik3ip1	299,6	541,0			963,2	1,62	1,97E-34		71,7	854,9	3,54	2,25E-102	566,0	2,95	3,04E-64
Pten	822,2	1070,8			1266,6				1143,6	3123,4			2498,6		
Rb1	37,8	63,6			73,1				55,2	43,8			36,5		
Trp53	97,4	75,1			45,6				134,9	56,4			86,3		
**Migration and invasion**															
Cyfip1	1639,9	2018,2			1945,2				2645,8	1952,1			1467,2		
Cyfip2	86,5	5,6			3,1				11,8	12,4			47,7		
Mmp7	0,0	0,0			0,0				0,0	0,0			0,0		
Mmp9	17,8	9,4			10,7				6,4	10,5			31,5		
Nckap1 (Nap1)	1635,1	2153,9			2055,5				1353,2	2853,5			1703,1		
**Epithelial-mesenchymal transition and reverse**															
Cdh1 (E-cadherin)	0,8	0,9			0,9				14,2	5,8			5,7		
Cdh2 (N-cadherin)	559,5	697,6			666,0				1273,9	2281,9			1776,9		
Cdh11 (OB-cadherin)	415,8	154,6	-1,37	6,32E-25	69,5	-2,47	1,10E-69		81,6	1235,2	3,88	7,39E-103	389,7	2,23	2,13E-30
Krt1	0,3	0,0			0,0				0,0	0,0			0,0		
Krt8	13,0	26,5			16,7				15372,0	82,4	-7,52	0,00E+00	10,1	-10,36	0,00E+00
Krt14	2,1	3,3			2,1				3,2	85,6	4,35	3,48E-23	34,9	3,07	3,29E-11
Krt18	1,8	1,6			0,7				13155,2	169,1	-6,26	0,00E+00	84,0	-7,26	0,00E+00
Snai1 (Snail)	650,5	540,3			621,0				210,1	169,2			328,1		
Snai2 (Slug)	249,7	443,5			515,0				460,9	1334,0			1036,5		
Twist1	856,7	792,5			868,4				1103,5	1437,4			1503,7		
Twist2	1888,2	1456,4			1039,0				19,2	9,0			30,9		
Vim (vimentin)	70081,6	94273,9			78654,6				32264,2	85570,7			81790,1		
Zeb1	815,2	933,9			1277,8				1372,3	2144,9			1327,7		
Zeb2	304,3	493,7			682,3	1,12	3,31E-18		853,2	1478,0			1169,2		
**Myogenesis markers**															
Cdh15 (M-cadherin)	0,3	1,9			0,0				13437,3	612,2	-4,34	3,24E-55	1083,3	-3,53	1,26E-32
Des	0,5	2,2			0,4				31936,2	379,2	-6,16	1,18E-77	624,9	-5,45	5,58E-54
Mef2c	187,5	104,0			131,3				1487,8	252,6	-2,54	4,93E-57	112,9	-3,68	2,56E-92
Myh1	0,8	0,0			0,2				2588,1	421,8	-2,48	5,79E-10	352,2	-2,71	2,10E-10
Myh3	0,0	1,6			0,0				6156,9	37,0	-6,70	1,10E-38	159,1	-4,77	1,63E-18
Myh7	0,0	0,0			0,0				502,5	13,0	-5,21	1,61E-113	7,2	-5,92	8,94E-79
Myh8	0,0	0,0			0,0				214,7	13,2	-3,96	3,54E-53	4,6	-5,25	2,71E-42
Myl1	0,0	0,3			0,0				8466,6	22,5	-8,02	4,11E-77	79,2	-6,31	5,28E-45
Myl4	13,0	11,1			12,1				5311,5	92,2	-5,42	3,81E-32	233,3	-4,16	1,72E-17
Mylk4	1,0	1,1			1,5				1324,5	23,3	-5,53	2,64E-45	28,1	-5,25	3,40E-36
Myod1	0,0	0,6			0,0				10725,3	173,0	-5,78	1,77E-85	615,0	-3,99	3,46E-37
Myog	0,3	2,2			0,2				28011,1	127,6	-7,19	3,05E-53	410,1	-5,61	1,66E-29
**Inflammation related**															
Nfatc1	269,8	275,0			497,6				70,9	484,4	2,75	5,71E-83	418,9	2,54	8,66E-65
Nfatc3	705,4	594,0			806,2				1066,6	1256,5			939,8		
Nfatc4	1306,8	1183,5			2003,0				192,7	1798,8	3,20	2,78E-138	879,8	2,18	1,42E-56

**Stemness markers**															
Bmi1	291,4	391,4			416,1				519,0	915,9			540,2		
Cd44	5587,5	3415,2			3218,9				5037,7	3382,0			4017,5		
Nanog	6,0	8,5			8,3				22,4	14,7			14,3		
Prom1 (Cd133; Prominin1	0,5	0,3			0,0				0,0	0,0			0,3		
Sox2	1159,5	1188,5			613,9				79,4	109,4			195,8		
**lncRNA**															
2610316D01Rik	0,0	0,0			0,0				0,0	0,0			0,0		
Hotair	0,0	0,0			0,0				0,0	0,0			0,0		
Malat1	880,1	775,3			2060,2	1,17	7,24E-16		578,1	841,6			2030,0		

[a] Mean of normalized counts in RNA-Seq experiment is shown. Genes have been sorted according to type or function (some genes have been listed twice). Green values correspond to high or increased values; red values correspond to low or decreased values. Log2FoldChange (with corresponding padj) are also listed for DE genes analyzed in Additional files 9 and 10: Tables S8 and S9.
