# Supplementary material for: Innovative mouse models for the tumor suppressor activity of Protocadherin-10 isoforms
Source: BMC Cancer. 2022 Apr 25;22:451. doi: 10.1186/s12885-022-09381-y (PMC9040349; doi:10.1186/s12885-022-09381-y)
Supplement: Supplementary file 24 — Additional file 24: comprising Figs. S12-S17. 24a: Fig. S12. Summary of differentially expressed (DE) genes in PTD7_RS (rescued by short isoform 1 of Pcdh10) versus malignant Pcdh10-lacking PTD7 cells. 24b: Fig. S13. Summary of DE genes in PTD25_RS (rescued by short isoform 1 of Pcdh10) versus malignant Pcdh10-lacking PTD25 cells. 24c: Fig. S14. Summary of DE genes in PTD25_RL (rescued by long isoform 4 of Pcdh10) versus malignant Pcdh10-lacking PTD25 cells. 24d: Fig. S15. Summary of DE genes shared by both PTD7_RS and PTD7_RL in comparison with malignant Pcdh10-lacking PTD7 cells. 24e: Fig. S16. Summary of DE genes shared by both PTD25_RS and PTD25_RL in comparison with malignant Pcdh10-lacking PTD25 cells. 24f: Fig. S17. Summary of DE genes in malignant Pcdh10-lacking PTD25 versus malignant Pcdh10-lacking PTD7 cells. [file 12885_2022_9381_MOESM24_ESM.pdf]

Additional file 24 for Kleinberger, Sanders, Staes et al. (2022)

Legend to additional file 24a: Fig. S12
Summary of differentially expressed (DE) genes in PTD7_RS (rescued by short isoform 1 of Pcdh10) versus malignant Pcdh10-lacking PTD7 cells. The analysis was performed by the Ingenuity Pathway Analysis software (IPA, Qiagen) (see also Table 2). Details on this analysis can be found in https://qiagen.secure.force.com/KnowledgeBase/KnowledgeIPAPage and https://digitalinsights.qiagen.com/products-overview/discovery-insights-portfolio/analysis-and-visualization/qiagen-ipa/. The top network score is based on a p-value calculation, which calculates the likelihood that the Network Eligible Molecules that are part of a network are found therein by random chance alone. The graphical summary below provides a coherent and comprehensible synopsis of the major biological themes in the IPA core analysis performed, including upstream regulators, diseases, functions, and pathways, and illustrates how those concepts relate to one another. The algorithm takes into account the magnitude of the differential expression of the corresponding gene in the dataset when deciding which regulators to include. In the left part, major DE genes are indicated in their subcellular compartment, with connecting lines indicating direct (solid lines), indirect (broken lines) or inferred (dotted lines) relationships. In the right part, molecular and cellular functions (octagons) influenced by Pcdh10 re-expression, as well as influenced canonical pathways (hour-glass symbols) and associated diseases (crosses) are listed. In an IPA core analysis, the z-score of a regulating molecule predicts the activation state of this upstream regulator, using the molecular expression patterns of the molecules downstream of an upstream regulator, being either increased for an activating gene or decreased for an inhibiting gene. Items with a positive z-score (activated) are colored orange, and those with negative z-score (inhibited) are colored blue. Network shapes, used here or in Additional file 24b-24f: Figs. S13-S17, include rectangles for cytokines, inverted triangles for kinases, horizontal ovals for transcriptional regulators, vertical rectangles for G-protein coupled receptors, and diamonds for non-kinase enzymes.

Additional file 24b: Figure S13. Summary of DE genes in PTD25_RS (rescued by short isoform 1 of Pcdh10) versus malignant Pcdh10-lacking PTD25 cells. The analysis was performed by the Ingenuity Pathway Analysis software (IPA, Qiagen) (see also Table 2). See Fig. 13 and Additional file 24a: Fig. S12 for more details.

Additional file 24c: Figure S14. Summary of DE genes in PTD25_RL (rescued by long isoform 4 of Pcdh10) versus malignant Pcdh10-lacking PTD25 cells. The analysis was performed by the Ingenuity Pathway Analysis software (IPA, Qiagen) (see also Table 2). See Fig. 13 and Additional file 24a: Fig. S12 for more details.

Additional file 24d: Figure S15. Summary of DE genes shared by both PTD7_RS and PTD7_RL in comparison with malignant Pcdh10-lacking PTD7 cells. The analysis was performed by the Ingenuity Pathway Analysis software (IPA, Qiagen) (see also Table 2). See Fig. 13 and Additional file 24a: Fig. S12 for more details.

Additional file 24e: Figure S16. Summary of DE genes shared by both PTD25_RS and PTD25_RL in comparison with malignant Pcdh10-lacking PTD25 cells. The analysis was performed by the Ingenuity Pathway Analysis software (IPA, Qiagen) (see also Table 2). See Fig. 13 and Additional file 24a: Fig. S12 for more details.

Additional file 24f: Figure S17. Summary of DE genes in malignant Pcdh10-lacking PTD25 versus malignant Pcdh10-lacking PTD7 cells. The analysis was performed by the Ingenuity Pathway Analysis software (IPA, Qiagen) (see also Table 2). See Fig. 13 and Additional file 24a: Fig. S12 for more details.

DEGenes_PTD7_RSvsPTD7_adapt - 2021-03-15 08:20 PM

Top Diseases and Bio Functions

Diseases and Disorders

Name	p-value range	# Molecules
Cancer	2,78E-04 - 1,12E-18	209
Organismal Injury and Abnormalities	2,90E-04 - 1,12E-18	288
Reproductive System Disease	9,49E-05 - 1,94E-12	143
Cardiovascular Disease	2,38E-04 - 9,46E-10	104
Skeletal and Muscular Disorders	2,63E-04 - 1,52E-09	153

Molecular and Cellular Functions

Physiological System Development and Function

Top Networks

ID	Associated Network Functions	Score
1	Tissue Development, Cancer, Endocrine System Disorders	41
2	Cancer, Organismal Injury and Abnormalities, Cardiovascular System Development and Function	39
3	Developmental Disorder, Organismal Injury and Abnormalities, Molecular Transport	37
4	Skeletal and Muscular Disorders, Tissue Morphology, Cancer	35
5	Cancer, Connective Tissue Disorders, Organismal Injury and Abnormalities	35

© 2000-2021 QIAGEN. All rights reserved.

DEGenes_PTD7_RSvsPTD7_adapt - 2021-03-15 08:20 PM Summary Graph

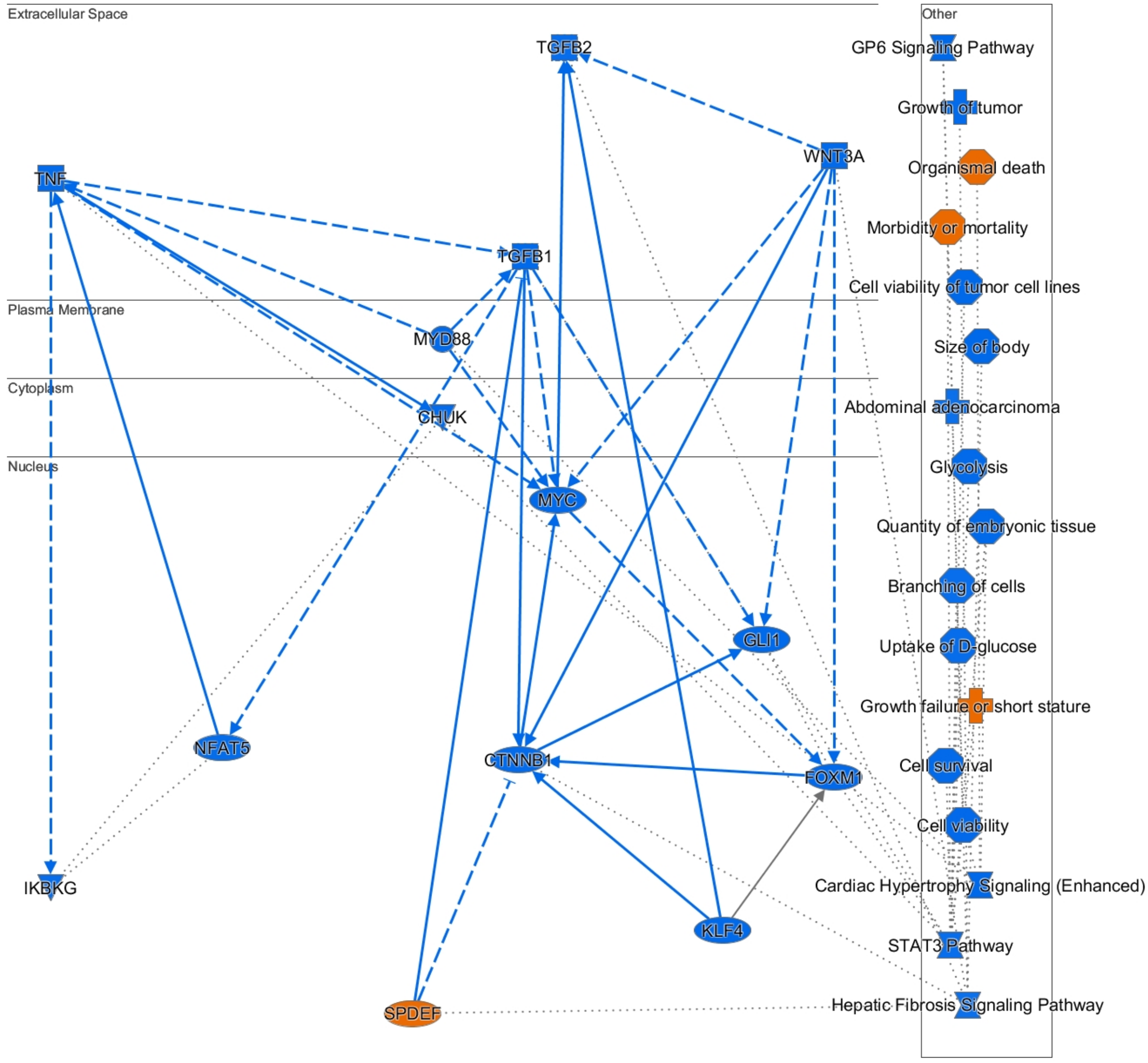

© 2000-2021 QIAGEN. All rights reserved.

Fig. S12

DEGenes_PTD25_RSvsPTD25_adapt - 2021-03-15 08:23 PM

Top Diseases and Bio Functions

Diseases and Disorders

Name	p-value range	# Molecules
Cancer	1,02E-09 - 1,22E-51	705
Organismal Injury and Abnormalities	1,02E-09 - 1,22E-51	983
Reproductive System Disease	3,74E-11 - 1,05E-31	441
Metabolic Disease	1,08E-11 - 3,03E-28	323
Endocrine System Disorders	1,25E-11 - 1,04E-27	343

Molecular and Cellular Functions

Physiological System Development and Function

Top Networks

ID	Associated Network Functions	Score
1	Cardiovascular Disease, Heart Failure, Organismal Injury and Abnormalities	36
2	Cellular Development, Connective Tissue Development and Function, Skeletal and Muscular System Development and Function	34
3	Cellular Development, Connective Tissue Development and Function, Embryonic Development	32
4	Cellular Assembly and Organization, Cellular Function and Maintenance, Hereditary Disorder	32
5	Cancer, Cell Cycle, Organismal Injury and Abnormalities	32

© 2000-2021 QIAGEN. All rights reserved.

DEGenes_PTD25_RSvsPTD25_adapt - 2021-03-15 08:23 PM Summary Graph

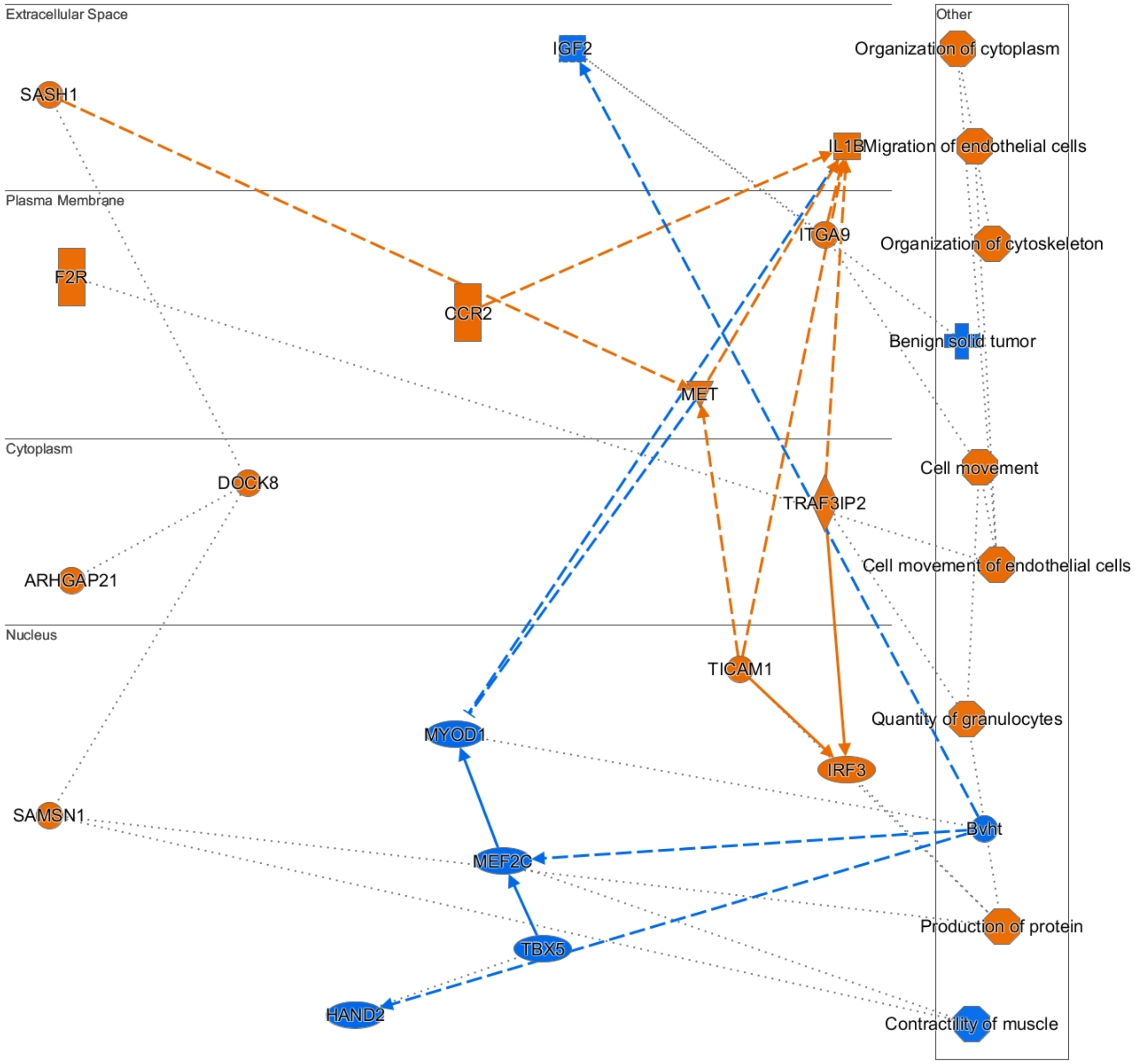

© 2000-2021 QIAGEN. All rights reserved.

Fig. S13

DEGenes_PTD25_RLvsPTD25_adapt - 2021-03-15 08:31 PM

Top Diseases and Bio Functions

Diseases and Disorders

Name	p-value range	# Molecules
Cancer	9,63E-11 - 5,32E-53	648
Organismal Injury and Abnormalities	9,67E-11 - 5,32E-53	904
Reproductive System Disease	9,63E-11 - 8,44E-34	435
Metabolic Disease	7,04E-12 - 6,75E-32	309
Endocrine System Disorders	9,63E-11 - 1,27E-30	324

Molecular and Cellular Functions

Physiological System Development and Function

Top Networks

ID	Associated Network Functions	Score
1	Embryonic Development, Organismal Development, Tissue Development	40
2	Cell Morphology, Cellular Assembly and Organization, Hair and Skin Development and Function	35
3	Lipid Metabolism, Molecular Transport, Small Molecule Biochemistry	33
4	Cancer, Organismal Injury and Abnormalities, Skeletal and Muscular System Development and Function	33
5	Organismal Injury and Abnormalities, Cardiovascular Disease, Neurological Disease	31

© 2000-2021 QIAGEN. All rights reserved.

DEGenes_PTD25_RLvsPTD25_adapt - 2021-03-15 08:31 PM Summary Graph

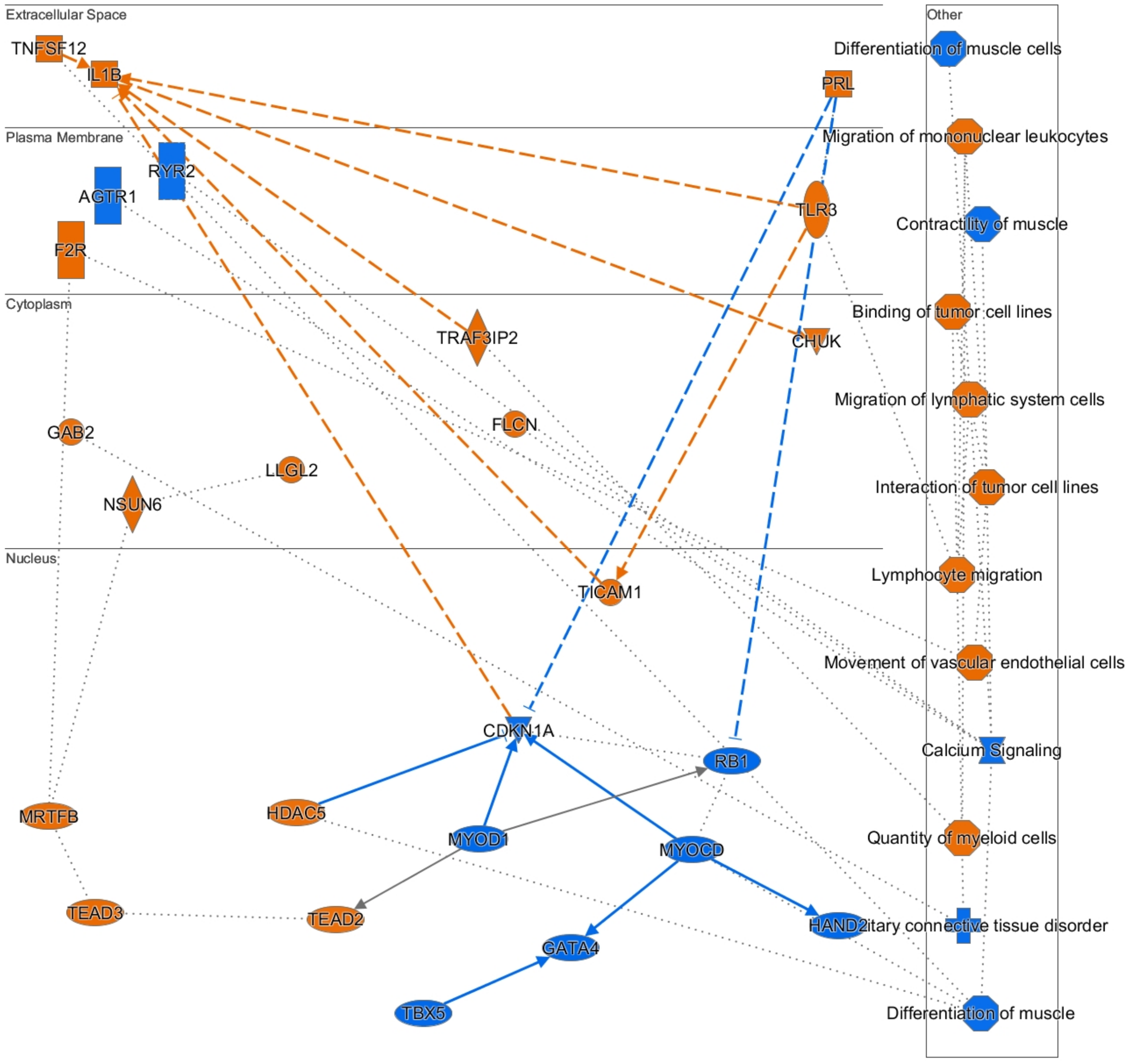

© 2000-2021 QIAGEN. All rights reserved.

Fig. S14

Shared_PTD7_RS_RLvsPTD7_adpat - 2021-03-15 08:32 PM

Top Diseases and Bio Functions

Diseases and Disorders

Name	p-value range	# Molecules
Cancer	2,74E-03 - 2,50E-08	120
Organismal Injury and Abnormalities	2,74E-03 - 2,50E-08	165
Cardiovascular Disease	2,67E-03 - 6,28E-08	69
Reproductive System Disease	2,68E-03 - 1,10E-06	79
Neurological Disease	2,34E-03 - 3,03E-06	85

Molecular and Cellular Functions

Physiological System Development and Function

Top Networks

ID	Associated Network Functions	Score
1	Lipid Metabolism, Small Molecule Biochemistry, Embryonic Development	45
2	Cardiovascular System Development and Function, Cellular Movement, Organismal Development	36
3	Cancer, Organismal Injury and Abnormalities, Neurological Disease	36
4	Respiratory System Development and Function, Organismal Development, Organismal Injury and Abnormalities	34
5	Cancer, Gastrointestinal Disease, Organismal Injury and Abnormalities	23

© 2000-2021 QIAGEN. All rights reserved.

Shared_PTD7_RS_RLvsPTD7_adpat - 2021-03-15 08:32 PM Summary Graph

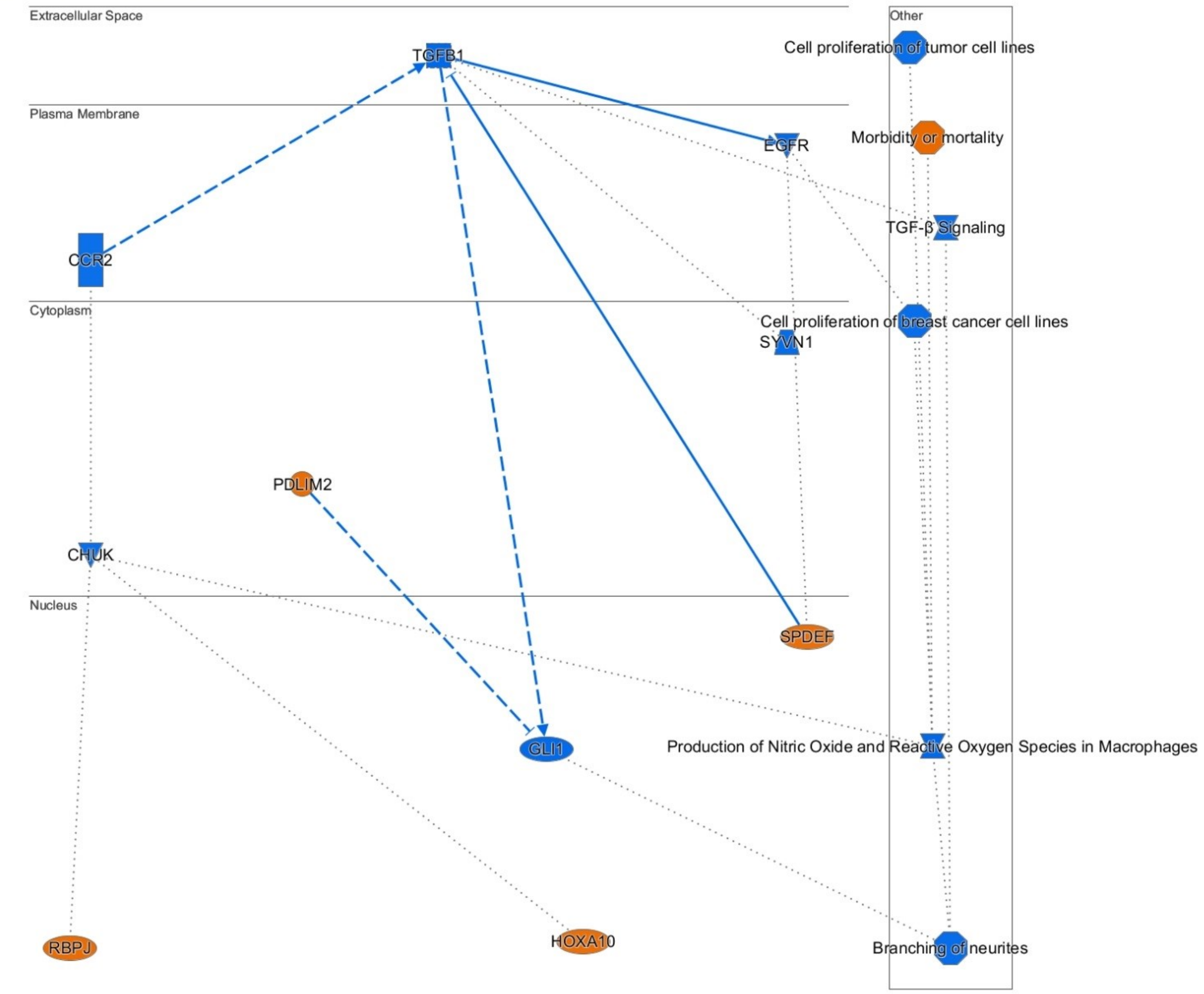

© 2000-2021 QIAGEN. All rights reserved.

Fig. S15

Shared_PTD25_RS_RLvsPTD25_adapt - 2021-03-15 08:33 PM

Top Diseases and Bio Functions

Diseases and Disorders

Name	p-value range	# Molecules
Cancer	7,95E-09 - 1,33E-43	536
Organismal Injury and Abnormalities	1,02E-08 - 1,33E-43	749
Reproductive System Disease	8,15E-09 - 3,85E-29	355
Metabolic Disease	5,06E-10 - 1,28E-28	260
Endocrine System Disorders	4,72E-09 - 8,07E-27	273

1 2 3 4 5 6 7 8 9 >

Molecular and Cellular Functions

Physiological System Development and Function

Top Networks

ID	Associated Network Functions	Score
1	Cardiovascular Disease, Heart Failure, Organismal Injury and Abnormalities	40
2	Organismal Injury and Abnormalities, Cancer, Tissue Development	36
3	Skeletal and Muscular System Development and Function, Cancer, Organismal Injury and Abnormalities	36
4	Cardiovascular System Development and Function, Embryonic Development, Organ Development	36
5	Cardiovascular System Development and Function, Cellular Development, Cellular Function and Maintenance	32

© 2000-2021 QIAGEN. All rights reserved.

Shared_PTD25_RS_RLvsPTD25_adapt - 2021-03-15 08:33 PM Summary Graph

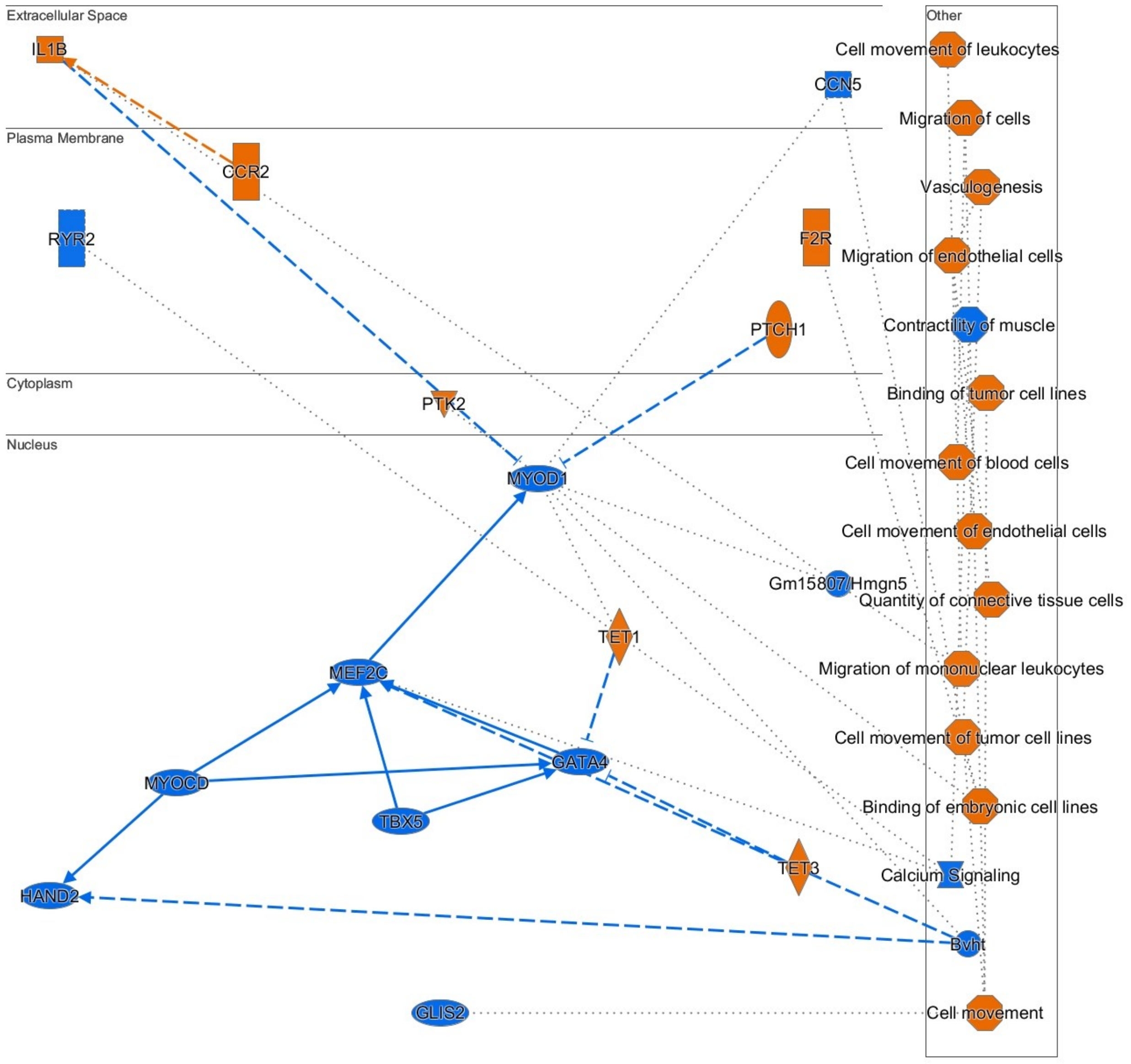

© 2000-2021 QIAGEN. All rights reserved.

Fig. S16

DEgenes_PTD25vsPTD7_adapt - 2021-03-15 06:25 PM

Top Diseases and Bio Functions

Diseases and Disorders

Name	p-value range	# Molecules
Cancer	5,28E-09 - 3,89E-40	650
Organismal Injury and Abnormalities	6,61E-09 - 3,89E-40	949
Cardiovascular Disease	6,45E-09 - 1,26E-30	388
Skeletal and Muscular Disorders	3,87E-09 - 2,04E-25	488
Metabolic Disease	4,52E-09 - 3,85E-25	303

Molecular and Cellular Functions

Physiological System Development and Function

Top Networks

ID	Associated Network Functions	Score
1	Developmental Disorder, Hereditary Disorder, Organismal Injury and Abnormalities	37
2	Cell Morphology, Connective Tissue Development and Function, Skeletal and Muscular System Development and Function	34
3	Cardiovascular System Development and Function, Organ Development, Organ Morphology	34
4	Cellular Development, Connective Tissue Development and Function, Skeletal and Muscular System Development and Function	34
5	Cellular Movement, Hematological System Development and Function, Immune Cell Trafficking	32

© 2000-2021 QIAGEN. All rights reserved.

DEgenes_PTD25vsPTD7_adapt - 2021-03-15 06:25 PM Summary Graph

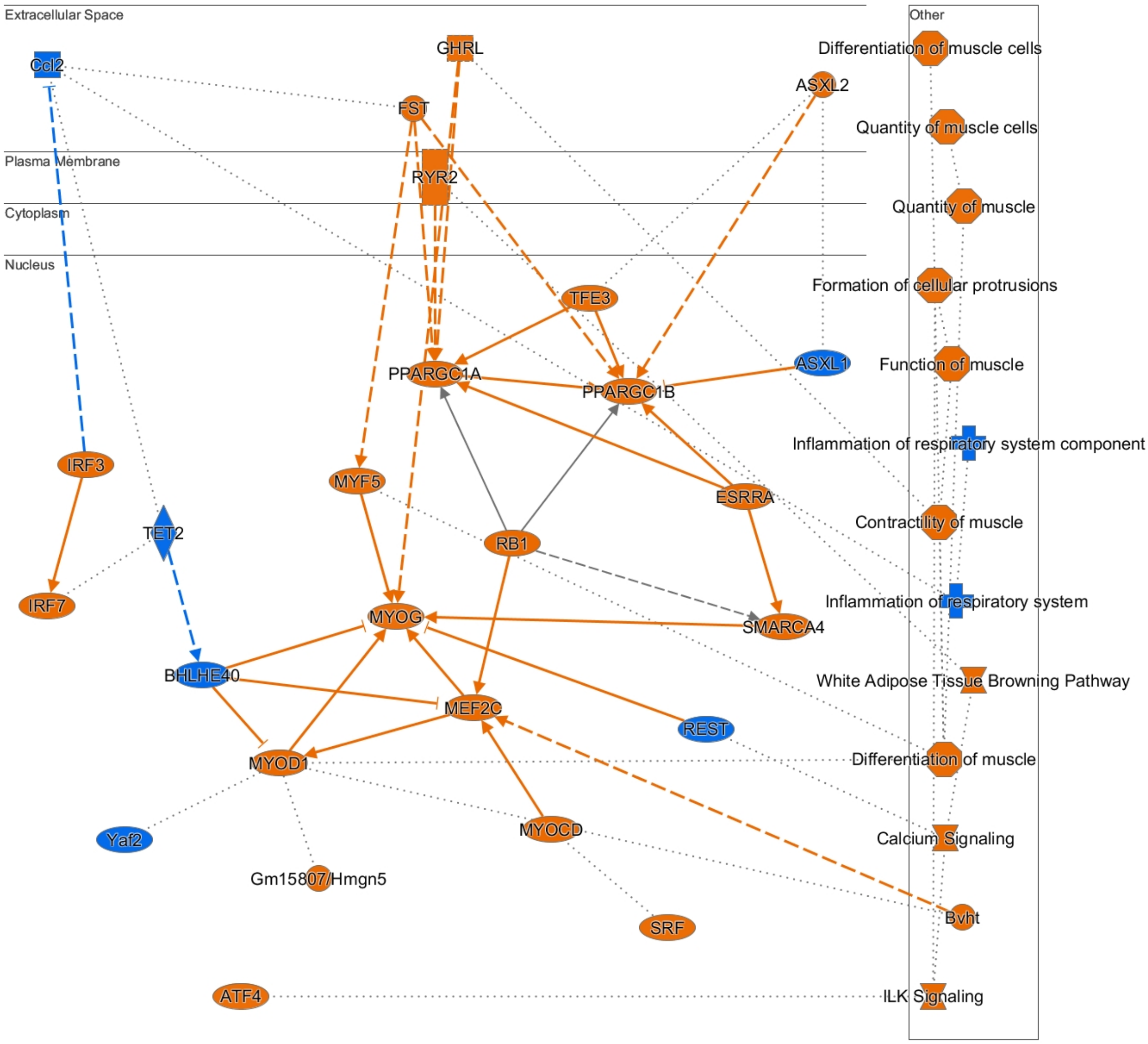

© 2000-2021 QIAGEN. All rights reserved.

Fig. S17
