# Supplementary material for: Innovative mouse models for the tumor suppressor activity of Protocadherin-10 isoforms
Source: BMC Cancer. 2022 Apr 25;22:451. doi: 10.1186/s12885-022-09381-y (PMC9040349; doi:10.1186/s12885-022-09381-y)
Supplement: Supplementary file 25 — Additional file 25: Fig. S18. IPA-generated ‘Cell death and survival’ network of genes differentially expressed in PTD25_RS (rescued by short isoform 1 of Pcdh10) versus malignant Pcdh10-lacking PTD25 cells. [file 12885_2022_9381_MOESM25_ESM.pdf]

Additional file 25 for Kleinberger, Sanders, Staes et al. (2022)

Legend to Figure S18. IPA-generated 'Cell death and survival' network of genes differentially expressed in PTD25_RS (rescued by short isoform 1 of Pcdh10) versus malignant Pcdh10-lacking PTD25 cells. The analysis was performed by the Ingenuity Pathway Analysis software (IPA, Qiagen) (see also Table 2 and legend to Fig. 14). The network graph depicts how a selection of DE genes relate to one another and to some key molecules. The genes are shown in their respective subcellular compartment, with connecting lines indicating direct (solid lines) or indirect (broken lines) relationships. In an IPA core analysis, the z-score of a regulating molecule predicts the activation state of this upstream regulator, using the molecular expression patterns of the molecules downstream of an upstream regulator, being either increased for an activating gene or decreased for an inhibiting gene. DE genes with a positive z-score (activated) are colored orange, and those with a negative z-score (inhibited) are colored blue. Underneath each of the DE genes is given: baseMean value (mean of normalized counts for all PTD25 and PTD25_RS samples), log2FoldChange (condition PTD25_RS versus PTD25), p_{adj} (Benjamin-Hochberg adjusted Wald test p-value). Network shapes, used here or in Figs. 14 and 15, include rectangles with solid rim for cytokines, rectangles with broken lines for growh factors, trapezia for transporters, vertical ovals for transmembrane receptors, vertical rectangles for G-protein coupled receptors, inverted triangles for kinases, triangles for phosphatases, vertical diamonds for non-kinase enzymes, horizontal diamonds for peptidases, horizontal ovals for transcriptional regulators, circles for other molecules, double-rimmed circles for complexes or groups.

Network 23 : DEGenes_PTD25_RSvsPTD25_adapt - 2021-03-15 08:23 PM : DEGenes_PTD25_RSvsPTD25 : DEGenes_PTD25_RSvsPTD25_adapt - 2021-03-15 08:23 PM

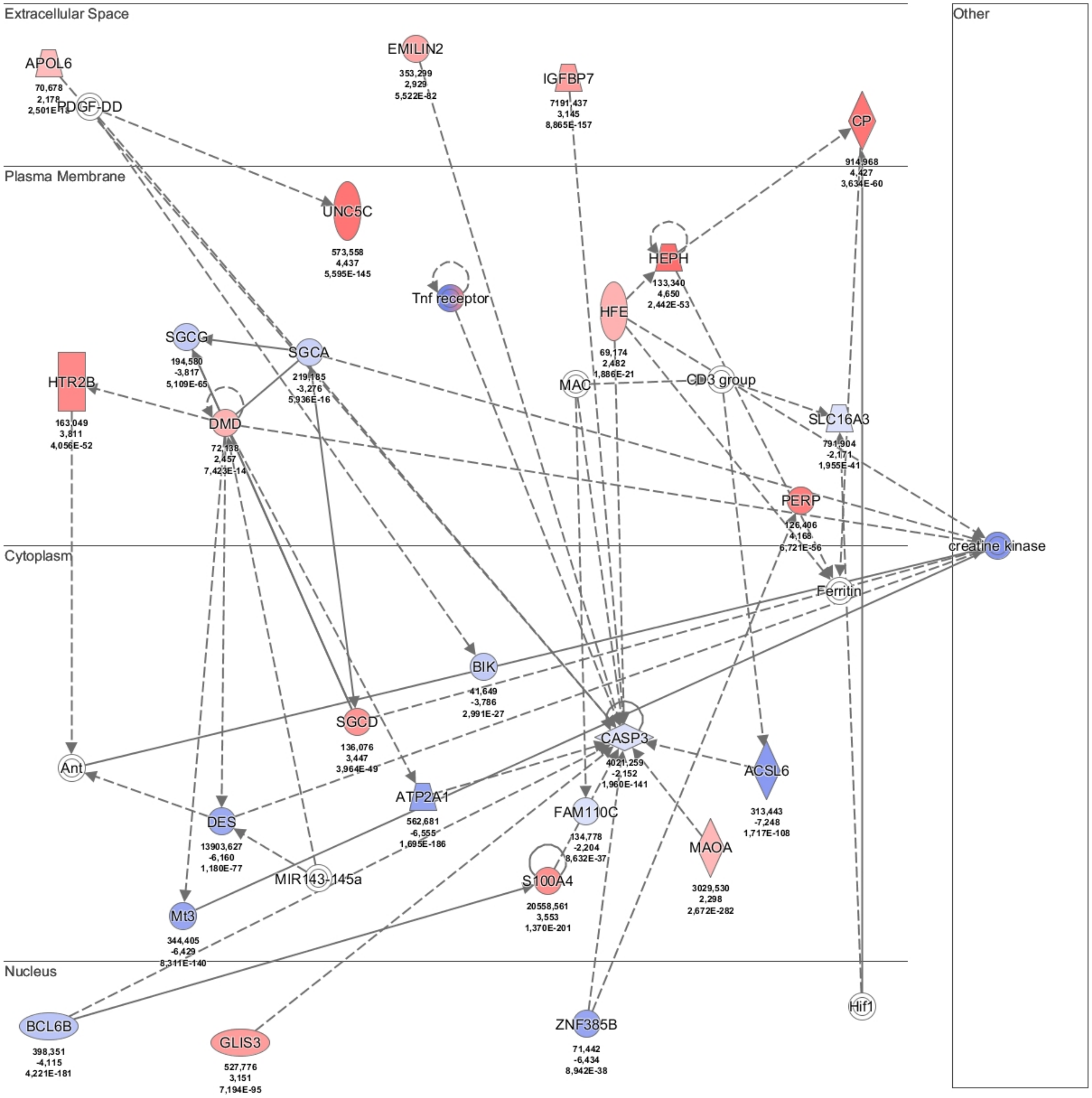

© 2000-2021 QIAGEN. All rights reserved.

Fig. S18
