# Supplementary material for: Innovative mouse models for the tumor suppressor activity of Protocadherin-10 isoforms
Source: BMC Cancer. 2022 Apr 25;22:451. doi: 10.1186/s12885-022-09381-y (PMC9040349; doi:10.1186/s12885-022-09381-y)
Supplement: Supplementary file 26 — Additional file 26. Original uncropped blots corresponding to Fig. 3, panels A-D (Pcdh10 expression levels in Pcdh10all mice by Western blot analysis). Antibodies used are indicated at the left. Exposure time was 1 min. [file 12885_2022_9381_MOESM26_ESM.pdf]

Additional file 26 for Kleinberger, Sanders, Staes et al. (2022)

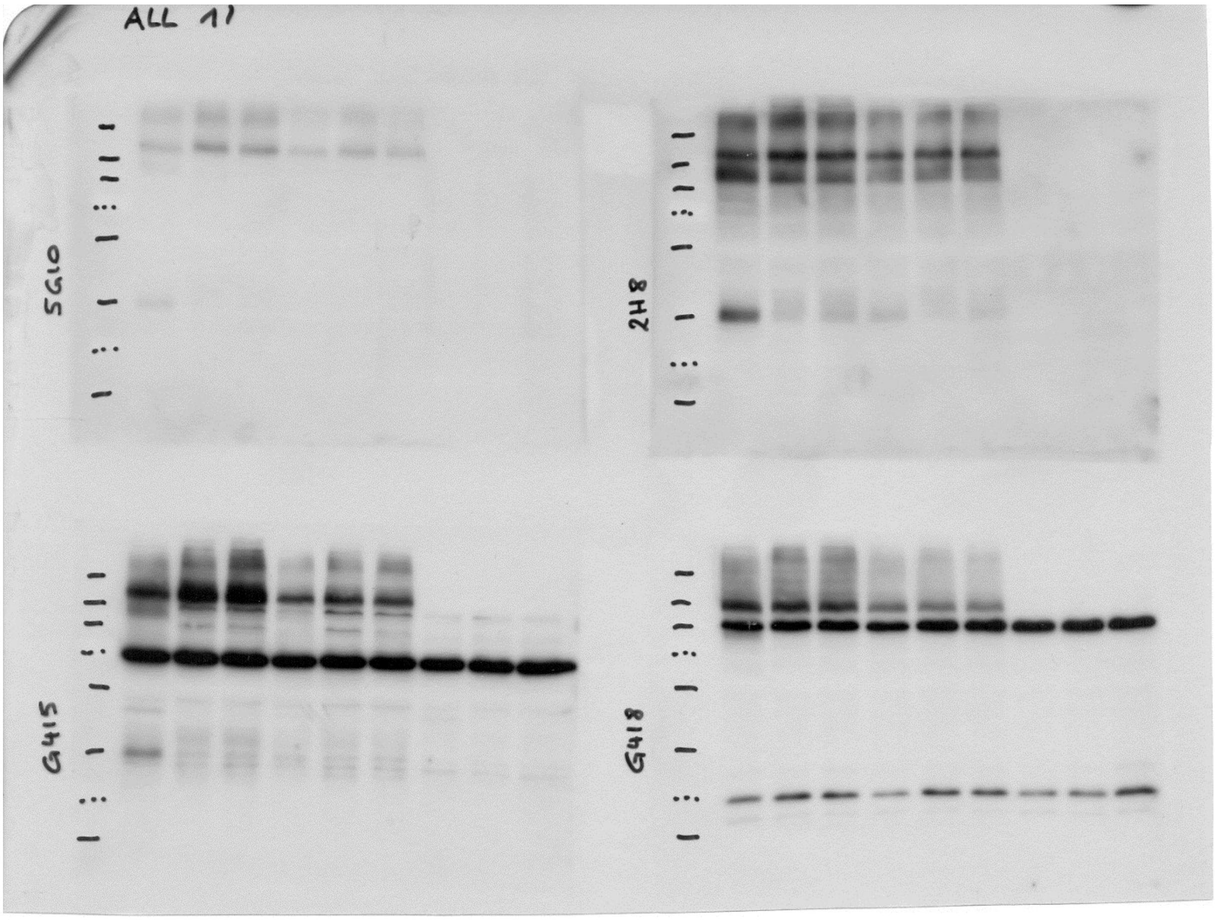

Additional file 26: Original uncropped blots corresponding to **Fig. 3, panels A-D** (Pcdh10 expression levels in Pcdh10all$^{-/-}$ mice by Western blot analysis). Antibodies used are indicated at the left. Exposure time was 1 min.
