# Supplementary material for: Innovative mouse models for the tumor suppressor activity of Protocadherin-10 isoforms
Source: BMC Cancer. 2022 Apr 25;22:451. doi: 10.1186/s12885-022-09381-y (PMC9040349; doi:10.1186/s12885-022-09381-y)
Supplement: Supplementary file 33 — Additional file 33. Original blots corresponding to Additional Fig. S3 (panel A, left side): Southern blot analysis of ES cells successfully targeted with the Pcdh10all targeting construct. [file 12885_2022_9381_MOESM33_ESM.pdf]

Additional file 33 for Kleinberger, Sanders, Staes et al. (2022)

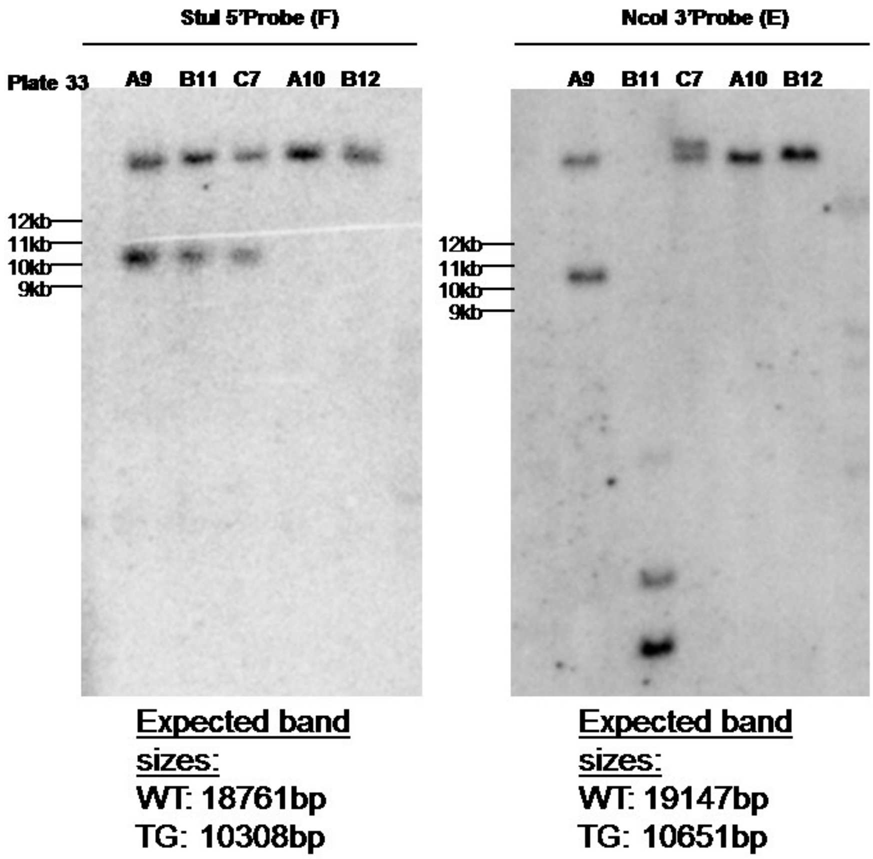

Additional file 33: Original blots corresponding to **Additional Figure S3 (panel A, left side)**: Southern blot analysis of ES cells successfully targeted with the Pcdh10all targeting construct. ES cell clone 33A9 (left lanes) shows the expected additional bands at 10.3 kb for the 5' probe (blot at the left) and at 10.6 kb for the 3' probe (blot at the right). Images of the fully uncropped versions of the blots are not anymore available due to departmental reorganizations. Cropping occurred on the image files after hybridization.
