# Supplementary material for: Innovative mouse models for the tumor suppressor activity of Protocadherin-10 isoforms
Source: BMC Cancer. 2022 Apr 25;22:451. doi: 10.1186/s12885-022-09381-y (PMC9040349; doi:10.1186/s12885-022-09381-y)
Supplement: Supplementary file 34 — Additional file 34. Original blots corresponding to Additional Fig. S3 (panel A, right side): Southern blot analysis of ES cells successfully targeted with the Pcdh10long targeting construct. [file 12885_2022_9381_MOESM34_ESM.pdf]

Additional file 34 for Kleinberger, Sanders, Staes et al. (2022)

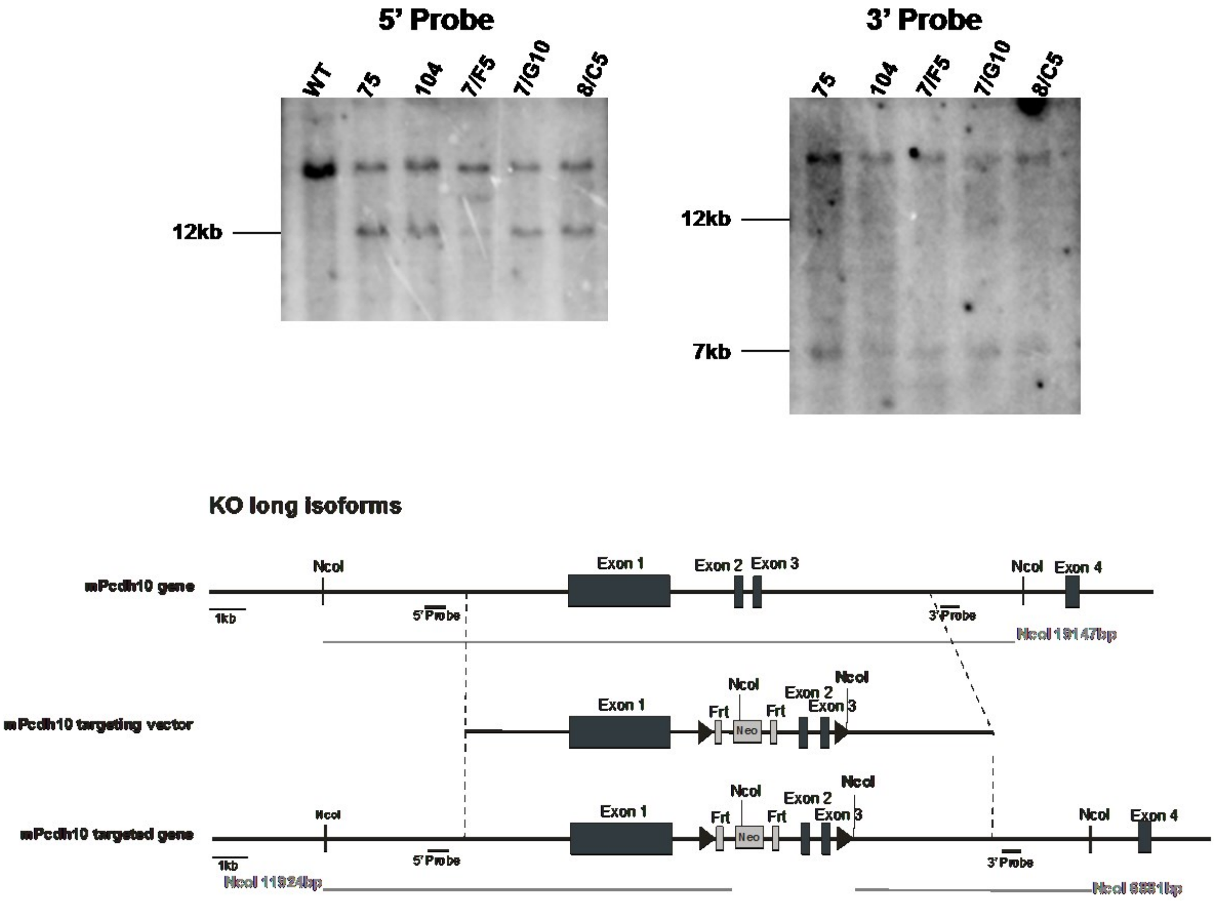

Additional file 34: Original blots corresponding to **Additional Figure S3 (panel A, right side)**: Southern blot analysis of ES cells successfully targeted with the Pcdh10long targeting construct. Several ES cell clones, including clone 7G10 show the expected additional bands (see scheme at the bottom) at 11.9 kb for the 5' probe (blot at the left) and at 6.8 kb for the 3' probe (blot at the right). Images of the fully uncropped versions of the blots are not anymore available due to departmental reorganizations. Cropping occurred on the image files after hybridization.
