# Supplementary material for: Innovative mouse models for the tumor suppressor activity of Protocadherin-10 isoforms
Source: BMC Cancer. 2022 Apr 25;22:451. doi: 10.1186/s12885-022-09381-y (PMC9040349; doi:10.1186/s12885-022-09381-y)
Supplement: Supplementary file 35 — Additional file 35. Animal Facility Procedures and Licenses of the VIB-UGent Center for Inflammation Research (IRC), Ghent, Belgium. [file 12885_2022_9381_MOESM35_ESM.pdf]

Inflammation Research Center
A VIB-UGENT DEPARTMENT

TO WHOM IT MIGHT CONCERN

FACILITY PROCEDURES AND LICENSES OVERVIEW
IRC-VIB ANIMAL HOUSE

The IRC-VIB Animal House consists of a SPF animal house, located at Technologiepark in 9052 Zwijnaarde and a conventional animal house, located at Proeftuinstraat in 9000 Ghent.

SPF Facility

In the SPF facility in Zwijnaarde, mice are housed in SPF-conditions, which requires a special housing system, the so-called Individualy Ventilated Cages (IVC's), and very strict hygienic measures. Our SPF animal house is divided in a breeding (licence number LA 24 00 526) and an experimental zone (licence number LA 14 00 091).

Breeding

The breeding rooms have separate dedicated staff. No researchers are allowed in the breeding zone, which consists of 6 separate units (rooms) with their own caretakers (2 people per room, Felasa B training). The total capacity of the breeding part is 4620 cages (type Greenline or Blueline, Tecniplast) with maximum 5 mice per cage.

The breeding area is separated from the rest the facility. People entering, change their clothes for autoclaved suits and socks, they disinfect their hands with Hibiscrub for one minute, they wear gloves, a mouth mask, hair net, and special animal house shoes that do not leave the area. A second autoclaved Tyvec is worn above the autoclaved suit.

All people enter through an air shower, all material that enters is autoclaved or H2O2 gassed. All cages, bedding, enrichment material, drinking water and food is autoclaved. All changing of cages is done in flow benches. The air coming in in the rooms is HEPA filtered. As a surface disinfectant, Virkon spray is used. People cannot enter the animal house for 48h if they were in contact with other rodents. The 48h period is chosen because people are supposed to have had two showers and changing of clothes in the meantime.

No mice can enter in the breeding zone unless by embryo transfer.

Experimental

The experimental zone consists of 4 separate animal rooms (total capacity: 2380 cages; maximum 5 mice per cage). Cages are changed by dedicated animal caretakers as described above.

Researchers can enter the experimental zone after obtaining their Felasa C degree and a custom-made introduction to the animal house given by the responsible veterinarian, who

VIB

Inflammation
Research Center

UGent

UGent- VIB Research
Building FSVM
Technologiepark 927
9052 GENT
BELGIUM
Tel: +32 9 331 36 00
Fax: +32 9 221 76 73
info@irc.ugent.be

www.vib.be
www.irc.ugent.be

is also the manager of the animal facility. People entering change their clothes for autoclaved suits and socks, they disinfect their hands with Hibiscrub for one minute, they wear gloves, a mouth mask, hair net, and special animal house shoes that do not leave the area. The same rules are applied for bringing in material and changing of the cages (see above).

BSL2

A smaller A2/L2 zone consists of 2 animal rooms and labs (total capacity of 560 IVC cages), each with an airlock and an extra changing of autoclaved clothes and special mouth masks and procedures. No material can leave these rooms without being autoclaved. All manipulations are performed in Biohazard flows.

Housing

The housing guidelines in appendix A van ETS123 June 2016 are respected. Mice are group housed and all cages have environmental enrichment (mouse houses, wood blocks, tissues). The animals have an 14/10h day/night cycle and have free access to Sniff food and water according to their metabolic stage (breeding or maintenance autoclavable food).

Blueline cages: each subgroup of mice (up to 4 mice) is housed in an IVC cage (type 1145) with a surface area of 435 cm².

Greenline cages: each subgroup of mice (up to 5 mice) is housed in an IVC cage (type GM500) with a surface area of 500 cm².

- Experiments can only be carried out after completing an ethical commission application, which is read and questioned by 12 internal and external ethical commission members (Ethical Commission University of Ghent-Faculty of Sciences/VIB). This Ethical Commission was approved by the Ministry.
- The health status of the mice is checked by complete annual health screenings and three-monthly screenings by an independent company (QM Diagnostics). Sentinels are housed on dirty bedding of the cages for at least 10 weeks.
- The washing room contains two throughput autoclaves of 900 liter (Wesa) and 1 of 565 liter (Belimed), a Rack Washer (Tecniplast) and an automated bedding dumping station connected to a vacuum container (Miltech).
- Animal welfare questions or issues are dealt with by the Animal Welfare Body, approved and controlled by the Flemish Government. This AWB consists of a veterinarian, a senior experienced postdoc researcher, biotechnicians and dedicated animal caretakers from each zone.

VIB

Inflammation Research Center

UGent

UGent- VIB Research Building FSVM
Technologiepark 927
9052 GENT
BELGIUM
Tel: +32 9 331 36 00
Fax: +32 9 221 76 73
info@irc.ugent.be

www.vib.be
www.irc.ugent.be

Conventional facility

Our conventional facility (licence number LA 14 00 091) is at a distance of 5 kilometres and does not share personnel or material. It consists of 10 separate animal rooms with a total capacity of 4600 cages (not reached yet). Part of the rooms contain IVC cages.

The washing room contains a two door autoclave of 1300 litre, a cabinet washer (Series 650 Cabinet washer, Tecniplast) and a custom made washing cabinet for racks (Tecniplast). The installation of an automated bedding dumping station is planned.

If any further information is required, please do not hesitate to contact me.

With kind regards,
Katrien Moerloose, DVM, Head of Animal Core Facility

UNIVERSITEIT GENT

IRC
A VIB-UGENT DEPARTMENT

VIB

Inflammation
Research Center

UGent

UGent- VIB Research
Building FSVM
Technologiepark 927
9052 GENT
BELGIUM
Tel: +32 9 331 36 00
Fax: +32 9 221 76 73
info@irc.ugent.be

www.vib.be
www.irc.ugent.be

Inflammation Research Center
A VIB-UGENT DEPARTMENT

The biosafety license and environmental permit numbers for the facilities:

1. UGent/VIB - Technologiepark 71, 9052 Gent-Zwijnaarde

Biosafety
Internal UGent-number: T60-1304
Biosafety license number : SBB 219.2014/0127.
Number and title of the activity: 1. “Biomedical molecular biological research”
User: Bart Lambrecht

Environmental permit
Internal UGent-number: V60-1301
Environmental permit: M03/44021/1584/1/A/1

2. Animalarium - Proeftuinstraat 86, 9000 Gent:

Biosafety
Internal UGent-number: T36-1302
Biosafety license number : SBB 219.2013/0144
Number and title of the activity: 3. "Experimental animals for molecular biomedical research”

Environmental permit
Internal UGent-number: V36-1212
Environmental permit: M03/44021/496/1/A/5

VIB

Inflammation
Research Center

UGent

UGent- VIB Research
Building FSVM
Technologiepark 927
9052 GENT
BELGIUM
Tel: +32 9 331 36 00
Fax: +32 9 221 76 73
info@irc.ugent.be

www.vib.be
www.irc.ugent.be
